# Bacterialized tumor cells as vaccine

**DOI:** 10.1038/s44321-026-00465-x

**Published:** 2026-06-19

**Authors:** Feng-Ying Huang, Ying-Ying Lin, Guang-Hong Tan

**Affiliations:** NHC Key Laboratory of Tropical Disease Control, School of Life Sciences and Medical Technology, Hainan Academy of Medical Sciences, Hainan Medical University, Haikou, China

**Keywords:** Cancer

## Abstract

Tumor vaccines represent a promising modality in cancer immunotherapy, but the limited immunogenicity of whole-tumor cell vaccines (WTCVs) restricts their clinical translation. Here, we developed a novel bacterialized tumor cell (BTC) vaccine by coating amino-functionalized bacterial lysates onto the surface of tumor cells, a process we define as “bacterialization”. In vitro and in vivo experiments demonstrated that BTCs efficiently promote dendritic cell (DC) phagocytosis, maturation, and antigen cross-presentation, enhance DC migration to draining lymph nodes (DLNs), and remodel the DLN microenvironment by expanding tumor-specific CD8⁺ T cells and memory CD8⁺ T cells, while reducing terminally exhausted CD8⁺ T cells. BTC vaccination elicited robust tumor-specific cellular and humoral immunity, which synergistically suppressed tumor growth and metastasis in five syngeneic murine tumor models with negligible adverse effects. Furthermore, BTC vaccination reversed the immunosuppressive tumor microenvironment by increasing functional CD8⁺ T cell infiltration and reducing inhibitory immune subsets, and exhibited significant synergistic anti-tumor effects when combined with anti-PD-1 immune therapy. These findings demonstrate that BTCs represent a safe and effective novel WTCV candidate with strong clinical translation potential.

## Synopsis

A novel “bacterialization” strategy was developed to engineer whole-tumor cell vaccines by coating amino-functionalized bacterial lysates onto tumor cell surfaces, resulting in bacterialized tumor cells (BTCs).

- BTCs significantly enhance DC phagocytosis, maturation, and antigen cross-presentation.
- BTC vaccination reverses the immunosuppressive tumor microenvironment in both the draining lymph nodes and tumor tissues.
- BTC vaccination elicits robust tumor-specific cellular and humoral immunity.
- BTC vaccination suppresses tumor growth and metastasis in five syngeneic murine tumor models.
- BTC vaccination exhibits strong synergistic anti-tumor effects with anti-PD-1 antibody therapy.

## Abstract (web summary)

A novel “bacterialization” strategy was developed to engineer whole-tumor cell vaccines by coating amino-functionalized bacterial lysates onto tumor cell surfaces, resulting in bacterialized tumor cells (BTCs).

## Graphical abstract

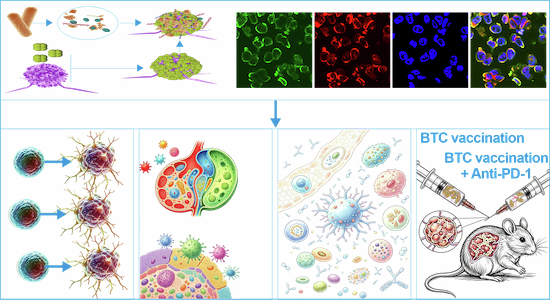

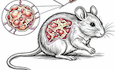

The paper explained **Problem**

Whole-tumor cell vaccines (WTCVs) with abundant broad-spectrum tumor-associated antigens effectively reduce tumor immune escape compared with conventional single-antigen personalized cancer vaccines. However, the clinical application of traditional inactivated WTCVs is severely limited by their weak intrinsic immunogenicity and insufficient dendritic cell-mediated antigen cross-presentation capacity. Most current modification strategies still fail to fully reverse the suppressive tumor microenvironment and trigger long-lasting durable anti-tumor immune responses, creating an urgent unmet need for safe, feasible, and high-performance WTCV engineering technologies for widespread clinical translation.

**Results**

We developed a facile and versatile surface bacterialization modification strategy to construct novel and effective bacterialized tumor cell vaccines (BTCs) by electrostatically coating positively charged amino-cationized mycobacterial lysates on the surface of irradiated tumor cells. Verified systematically in five different murine tumor models covering multiple common cancer types, BTCs significantly promote dendritic cell phagocytosis, maturation, and efficient antigen cross-presentation. They reshape the immunosuppressive immune microenvironments in both lymph nodes and tumor tissues, expand functional tumor-specific memory CD8^+^ T cells, and effectively relieve terminal T cell exhaustion. BTC vaccination efficiently inhibits tumor growth, distant metastasis, and postoperative recurrence with negligible side effects, eliciting potent synergistic cellular and humoral anti-tumor immunity. Moreover, BTC treatment exhibits remarkable synergistic therapeutic effects when combined with clinical anti-PD-1 immune checkpoint immunotherapy.

**Impact**

This study establishes an innovative and clinically feasible bacterialization engineering platform for targeted WTCV optimization. Distinct from traditional complicated genetic or cytokine modification strategies, this simple and robust approach endows whole tumor cells with active bacterial pathogen-associated molecular patterns to potently activate both innate and adaptive anti-tumor immune cascades in vivo. By completely overcoming the core immunogenicity bottleneck of conventional WTCVs, BTCs display universal anti-tumor efficacy and reliable clinical safety, providing a promising translatable vaccine candidate and a novel design paradigm for next-generation personalized cancer immunotherapeutic vaccines.

## Introduction

Tumor vaccines harness the host immune system to specifically target malignant cells and have emerged as a promising therapeutic modality in cancer immunotherapy. Although antigen-specific vaccines have been clinically deployed for cancer treatment, extensive inter-patient antigen heterogeneity and the high cost of identifying personalized mutational antigens greatly limit their broad clinical applicability (Diao and Liu, 2023; Fan et al, 2023; Pérez-Baños et al, 2023). Accordingly, whole-tumor cell vaccines (WTCVs) have attracted increasing research attention as an advanced subclass of tumor vaccines (Pérez-Baños et al, 2023). WTCVs contain a broad spectrum of endogenous tumor-associated antigens that can trigger comprehensive immune responses against multiple tumor-specific antigens across diverse cancer types. This polyvalent antigenic profile effectively reduces the risk of tumor immune escape (Diao and Liu, 2023). Nevertheless, inactivated whole-tumor cells generally exhibit weak intrinsic immunogenicity, restricting their therapeutic potency. To address this limitation, multiple adjuvant-based modification strategies have been developed. Representative approaches include conjugating CpG-ODNs to inactivated hepatocellular carcinoma cells and combining this formulation with near-infrared laser irradiation to enhance tumor immunogenicity (Diao and Liu, 2023; Meng et al, 2023; Pérez-Baños et al, 2023). Moreover, recent studies have validated that supplementing WTCVs with immune-stimulatory mediators, such as granulocyte-macrophage colony-stimulating factor and interleukin-2, can markedly amplify systemic anti-tumor immune reactions (Dranoff et al, 1993; Liu et al, 2013; Stagg et al, 2004; Wang et al, 2025). Despite these advances, the overall therapeutic efficacy of current WTCVs remains unsatisfactory and fails to meet clinical translation demands. Therefore, the development of novel and reliable engineering strategies to optimize WTCV performance is an urgent unmet need in this field.

Antigen cross-presentation serves as a core immunological process whereby antigen-presenting cells (APCs) internalize exogenous antigens derived from tumor vaccines and present processed antigenic peptides to CD8⁺ cytotoxic T lymphocytes, initiating tumor-specific adaptive immune responses (Bevan, 2006; Joffre et al, 2012; Luri-Rey et al, 2025). After internalization, APCs degrade tumor antigens into short peptide fragments, which bind to major histocompatibility complex class I (MHC-I) molecules. These peptide-MHC-I complexes are subsequently transported to the cell surface to activate CD8⁺ T cells and drive their differentiation into functional cytotoxic T lymphocytes (CTLs) (Colbert et al, 2020; Matheoud et al, 2010; Neefjes et al, 2011). Mounting evidence confirms that CTL-mediated cellular immunity is indispensable for efficient tumor suppression (Joffre et al, 2012). To elicit robust tumor-specific CTL responses, dendritic cells (DCs) must efficiently internalize vaccine-derived antigens, execute intracellular processing, and deliver antigenic signals to CD8⁺ T cells via the MHC-I cross-presentation pathway (Neefjes et al, 2011). Hence, enhancing the efficiency of antigen cross-presentation is a critical bottleneck for the rational design and clinical advancement of next-generation tumor vaccines.

Pathogenic bacteria express highly conserved structural molecules distinct from host-derived components, collectively termed pathogen-associated molecular patterns (PAMPs) (Matheoud et al, 2010). Immune cells recognize PAMPs through germline-encoded pattern recognition receptors (PRRs) distributed on the cell surface and within intracellular compartments. Toll-like receptors (TLRs) represent the most well-characterized PRR family and are widely expressed across multiple immune cell subsets (De Lorenzo et al, 2018). The specific recognition of bacterial PAMPs by TLRs and other PRRs enables the host immune system to detect exogenous pathogenic threats and initiate comprehensive immune activation cascades (Cao and Kagan, 2023; Chi et al, 2024; De Lorenzo et al, 2018). Activated immune cells exhibit enhanced pathogen phagocytosis, secrete pro-inflammatory cytokines and chemokines, recruit and activate APCs, and upregulate MHC molecules and co-stimulation signals. These collective alterations accelerate antigen processing and presentation, driving both innate and adaptive immune activation (Borriello et al, 2022). Notably, bacterial PAMPs and other microbial structural components can endow modified tumor cells with bacterial-like molecular characteristics. This functional modification enables APCs to recognize engineered tumor cells as exogenous pathogenic targets, thereby promoting their active engulfment and antigen processing (Demento et al, 2011; McBride et al, 2024). This unique mechanism provides a feasible strategy to potentiate APC-mediated uptake of WTCVs, optimize antigen presentation efficiency, and ultimately strengthen tumor-specific immune responses.

In the present study, we developed a facile surface modification strategy to functionalize bacterial lysates via amino cationization. The positively charged aminated bacterial lysates efficiently bind to the negatively charged plasma membrane of irradiated tumor cells, achieving stable surface coating. We define this tumor cell modification process as “bacterialization”, and the resulting engineered tumor cells are designated as bacterialized tumor cells (BTCs). Comprehensive in vitro and in vivo experiments demonstrated that BTC-based vaccines trigger potent tumor-specific cellular and humoral immune responses, exhibit robust anti-tumor activity in multiple syngeneic tumor models, and possess favorable clinical translation potential.

## Results

### Preparation and characterization of bacterialized tumor cells

**Figure 1:**
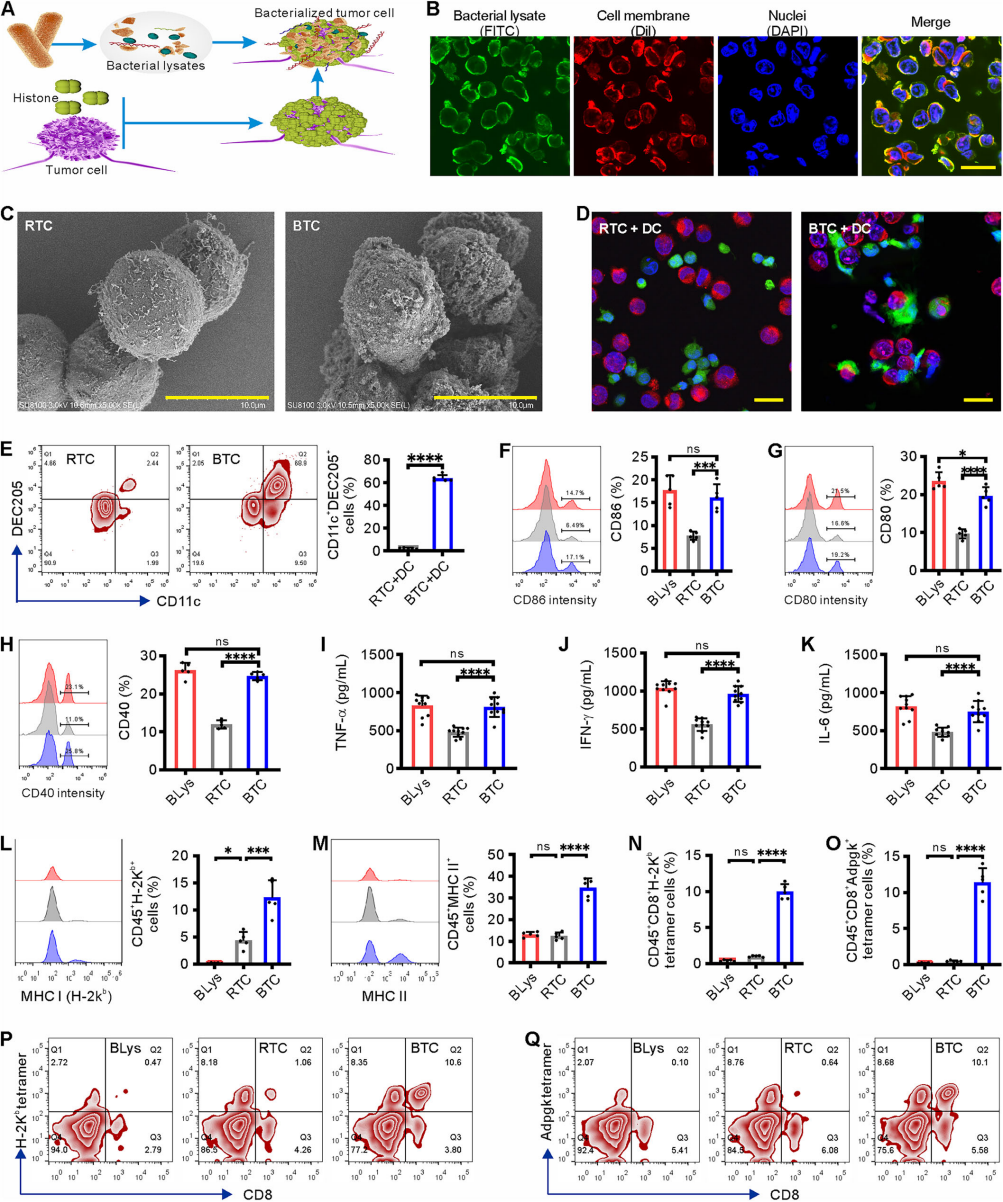
Preparation and characterization of bacterialized tumor cells and their capacity to phagocytose, mature, and cross-present antigens by dendritic cells. (**A**) Diagram illustrating the procedure for preparing bacterialized tumor cells (BTCs). (**B**, **C**) Successful encapsulation of a bacterial lysate on the surface of tumor cells was observed under a confocal microscope (**B**, scale bar, 20 μm) and a scanning electron microscope (**C**, scale bar, 10 μm). (**D**) Morphological changes of murine monocyte-derived DCs (moDCs, green) in engulfing and processing BTCs and radiation-attenuated tumor cells (RTCs, red), Scale bar, 20 μm. (**E**) Levels of moDCs engulfing BTCs and RTCs labeled in the same way in (**D**) were analyzed by flow cytometry (*n* = 5 in each group; ****P < 0.0001). (**F**–**H**) moDCs were incubated with BLyS, RTC, or BTC for 24 h (**F**–**M**). Flow cytometry was used to analyze the expression levels of moDC maturation markers CD86 (**F**; *n* = 5, ***P = 0.0002), CD80 (**G**; *n* = 5, *P = 0.0256 and ****P < 0.0001), and CD40 (**H**; *n* = 5, ****P < 0.0001). (**I**–**K**) Titers of cytokines IFN-α (**I**; *n* = 5, ****P < 0.0001), IFN-γ (**J**; *n* = 5, ****P < 0.0001), and IL-6 (**K**; *n* = 5, ****P < 0.0001) in the indicated groups were determined by ELISA. (**L**, **M**) Antigen (H-2Kb)-specific MHC I (**L**; *n* = 5, *P = 0.0171, ***P = 0.001) and MHC II (**M**; *n* = 5, ****P < 0.0001) expression on the moDCs in the indicative groups was analyzed by flow cytometry. (**N**–**Q**) Percentage of CD8 cells expressing H-2Kb-specific (**N**, **P**; *n* = 5, ****P < 0.0001) or Adpgk-specific (**O**, **Q**, *n* = 5, ****P < 0.0001) tetramers in the local lymph nodes from mice immunized with the indicated formulation. Data are presented as mean ± SD of five independent experiments. Two-way ANOVA with Tukey multiple comparisons: *P < 0.05, ***P < 0.001, ****P < 0.0001, NS: non-significant. See also Figs. EV1 and EV2; Movie EV1. Source data are available online for this figure.

**Figure EV1:**
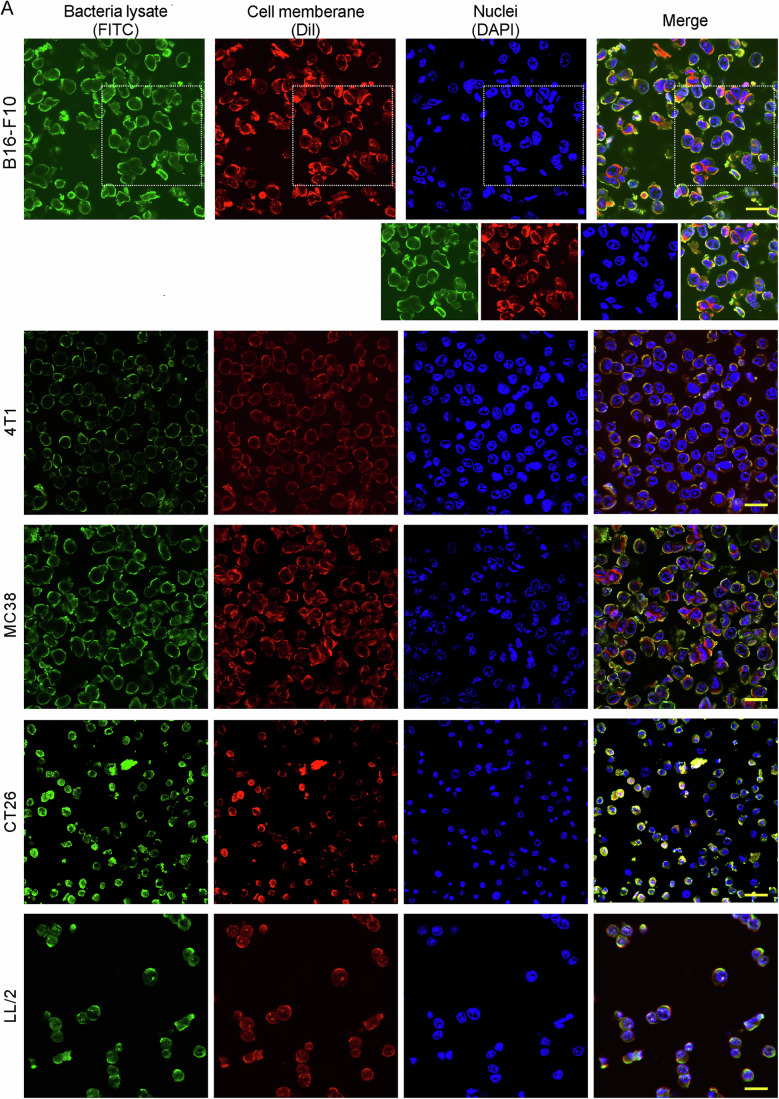
Preparation and characterization of BTCs, related to Fig. 1. (**A**) Five different types of BTCs, including B16-F10, 4T1, MC38, CT26, and LL/2 BTCs, were observed using a confocal microscope. FITC (green) was used to label bacterial lysates, Dil (red) for tumor cell membranes, and DAPI (blue) for nuclei.

BTCs were generated via amine functionalization of bacterial lysates. Briefly, negatively charged carboxyl groups within bacterial lysate proteins were chemically converted to positively charged amino groups, yielding cationized lysate components. These positively charged derivatives were then incubated with irradiated tumor cells to facilitate electrostatic binding and membrane surface coating. Additional incubation with crude bacterial lysate further enhanced surface component adsorption and coating stability (Fig. 1A). Herein, the term “bacterialization” is explicitly defined as the engineering process of decorating irradiated tumor cell surfaces with aminated bacterial lysates to generate BTCs. Confocal laser scanning microscopy verified the successful construction of BTCs across five murine tumor cell lines (B16-F10, MC38, CT26, 4T1, and LL/2). Fluorescence co-localization of FITC-labeled bacterial lysates, DiI-stained tumor cell membranes, and DAPI-labeled nuclei confirmed the uniform deposition of bacterial components on the tumor cell surface (Figs. 1B and EV1). Scanning electron microscopy further revealed distinct surface morphological differences: BTCs displayed a rough and irregular membrane microstructure, whereas radiation-attenuated tumor cells (RTCs) retained a smooth surface (Fig. 1C). These morphological results collectively validated the stable and successful preparation of BTCs. For concise nomenclature, RTCs and BTCs derived from individual cell lines are uniformly abbreviated in a cell-specific manner throughout the manuscript (e.g., 4T1 BTC, MC38 RTC).

### BTCs promote DC phagocytosis, maturation, and antigen cross-presentation

**Figure EV2:**
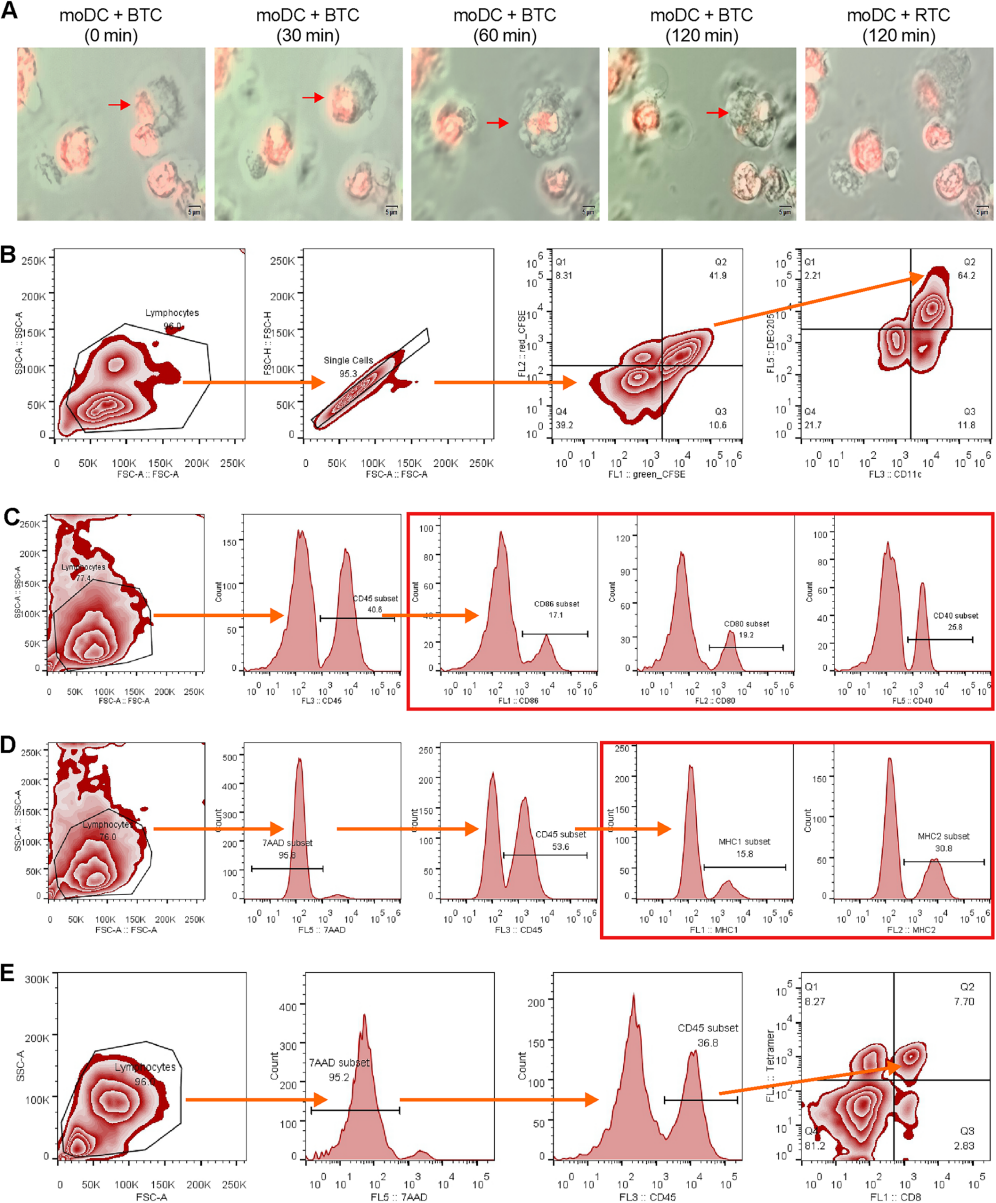
BTCs increase the ability of DCs to engulf, develop, and present antigens through cross-presentation, related to Fig. 1. (**A**) Observation of the dynamic process of DCs engulfing and processing BTC. See also the Movie EV1. (**B**) The gating strategy for flow cytometry analysis in Fig. 1E. Debris and duplicate cells were excluded first, followed by gating out the green and far red CFSE-positive cells to analyze the CD11c and DEC205 double-positive cells. (**C**) The gating strategy for flow cytometry analysis in Fig. 1F–H. Debris was excluded first, followed by gating out the CD45-positive cells to analyze the CD86, CD80, and CD40-positive cells, respectively. (**D**) The gating strategy for flow cytometry analysis in Fig. 1L,M. Debris was excluded first, followed by gating out the 7-AAD-negative and CD45-positive cells to analyze the MHC1- or MHC2-positive cells. (**E**) The gating strategy for flow cytometry analysis in Fig. 1N–Q. Debris was excluded first, followed by gating out the 7-AAD-negative and CD45-positive cells to analyze the CD8 and tetramer double-positive cells.

To assess DC-mediated phagocytosis of BTC, murine monocyte-derived DCs (moDCs) were co-cultured with B16-F10 BTC, and dynamic cellular behaviors were monitored via live-cell imaging. Time-lapse observations showed that moDCs rapidly migrated toward BTC and formed a tight cell-cell encapsulation. Within 30 min of co-culture, moDCs underwent a typical dendritic morphological transformation and initiated partial phagocytosis of BTC; complete internalization and partial degradation of BTC were observed at 60 min, with robust degradation detected at 120 min (Fig. EV2A; Movie EV1). In contrast, moDCs co-cultured with RTC exhibited no obvious migration, morphological alteration, or cell engulfment behavior (Fig. EV2A). Confocal microscopy imaging further confirmed the differential phagocytic capacity of moDCs towards BTC and RTC. When co-cultured with RTC, moDCs maintained a resting morphology with negligible cell-cell contact. However, BTC stimulation induced evident morphological activation and full encapsulation of target cells by moDCs (Fig. 1D). Flow cytometry quantitative analysis showed that the proportion of CD11c⁺DEC205⁺ active moDCs was markedly elevated in the BTC co-culture group, with an increase of more than 60-fold compared to the RTC group (Figs. 1E and EV2B). The OVA-4T1 BTC, containing the H2Kb neoantigen, was used to investigate its impact on the maturation and antigen presentation of moDCs (gating strategy shown in Fig. EV2C–E). Compared to the blank bacterial lysate (BLys) and RTC control groups, moDCs stimulated with the OVA-4T1 BTC exhibited significantly upregulated expression of the classic DC maturation markers CD80, CD86, and CD40 (Figs. 1F–H and EV2C). Meanwhile, the secretion levels of immunostimulatory cytokines IFN-α, IFN-γ, and IL-6 were substantially increased in the OVA-4T1 BTC-treated moDC supernatant (Fig. 1I–K). For antigen presentation function, OVA-4T1 BTC treatment efficiently enhanced the formation of H2Kb-OVA peptide-MHC-I complexes and upregulated surface MHC-II expression in moDCs (Figs. 1L,M and EV2D). In vivo vaccination experiments further verified the superior antigen presentation capacity of BTCs. Mice immunized with OVA-4T1 BTCs or neoantigen Adpgk-expressing MC38 BTCs presented increased frequencies of neoantigen-specific tetramer-positive CD45⁺CD8⁺ T cells in the draining lymph nodes (DLNs) (Figs. 1N–Q and EV2E). These data demonstrate that BTCs efficiently facilitate DC internalization of BTC antigens, promote robust DC maturation and activation, and enhance both MHC-I-dependent cross-presentation and MHC-II-mediated antigen presentation.

### BTCs facilitate DC migration and alter the immune microenvironment in draining lymph nodes

**Figure EV3:**
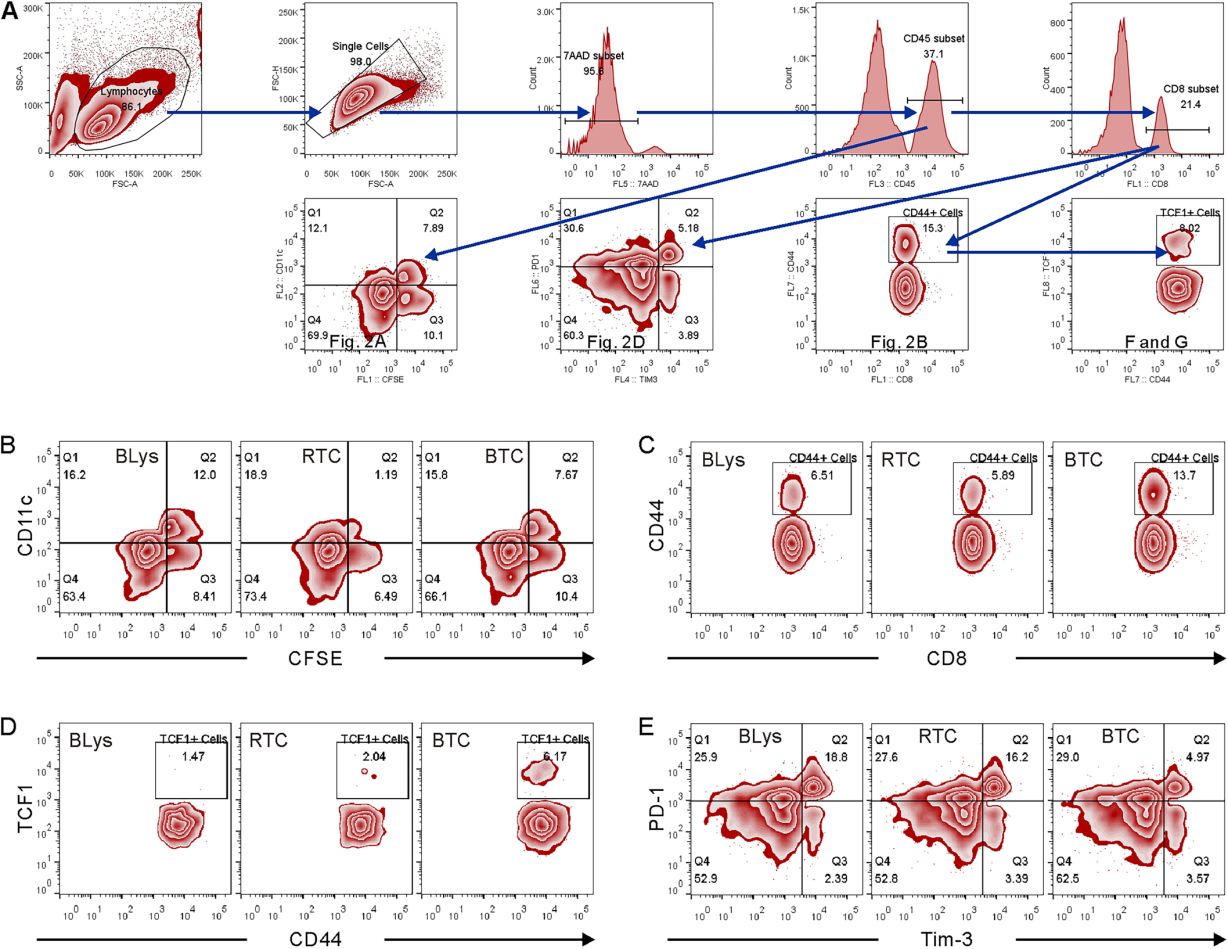
BTCs facilitate DC migration and reshape the immune microenvironment in draining lymph nodes, related to Fig. 2. (**A**) The gating strategy for flow cytometry analysis in Fig. 2. Debris was excluded first, followed by gating out the 7-AAD-negative and CD45-positive cells to analyze the CFSE and CD11c double-positive cells or CD8-positive cells. The CD8-positive cells were further analyzed for TIM3 and PD1 double-positive or CD44-positive cells, which were then further analyzed for TCF-1-positive cells. (**B**) Representative images of flow cytometry analysis (MC38) related to Fig. 2A. (**C**) Representative images of flow cytometry analysis (MC38) related to Fig. 2B. (**D**) Representative images of flow cytometry analysis (MC38) related to Fig. 2C. (**E**) Representative images of flow cytometry analysis (MC38) related to Fig. 2D.

**Figure 2:**
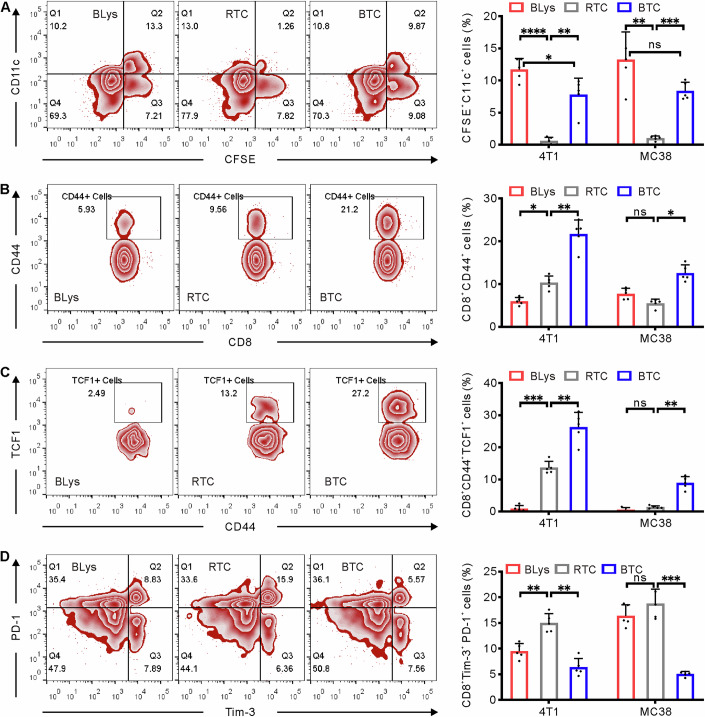
BTCs facilitate DC migration and reshape the immune microenvironment in draining lymph nodes. Mice were vaccinated with the indicated formulations labeled with CFSE (green). After 24 h, single cells from the local lymph nodes were collected for flow cytometry analysis. (**A**) Typical flow cytometry image (left) and the percentage of CD45^+^CD8^+^CFSE^+^CD11c^+^ cells, which are thought to be DCs that have taken up CFSE-labeled formulations and migrated to the lymph nodes (*n* = 5), *P = 0.0378, **P = 0.0045 (4T1), **P = 0.0079, ***P = 0.0009 and ****P < 0.0001. (**B**) Typical flow cytometry image (left) and the percentage of CD45^+^CD8^+^CD44^+^ cells (*n* = 5), *P = 0.0197 (4T1), **P = 0.0043 and *P = 0.0100. (**C**) Typical flow cytometry image (left) and the percentage of CD45^+^CD8^+^CD44^+^TCF1^+^ cells (*n* = 5), ***P = 0.0001, **P = 0.0033 (4T1) and **P = 0.0017. (**D**) Typical flow cytometry image (left) and the percentage of CD45^+^CD8^+^Tim3^+^PD^−^1^+^ cells (*n* = 5), **P = 0.0031 (BLys vs. RTC), **P = 0.0042 and ***P = 0.0010. Data are presented as mean ± SD. Two-way ANOVA with Tukey multiple comparisons: *P < 0.05, **P < 0.01, ***P < 0.001, ****P < 0.0001, NS: non-significant. See also Fig. EV3. Source data are available online for this figure.

Effective migration of antigen-loaded DCs to regional DLNs is a prerequisite for initiating systemic anti-tumor T cell immunity (Carbone et al, 2004). To explore whether BTCs enhance DC migration in vivo, CFSE-labeled 4T1 and MC38 BTCs, RTCs, and BLys were subcutaneously injected into mice, and DLN immune cell subsets were analyzed by flow cytometry using the gating strategy shown in Fig. EV3A. The frequency of CD45⁺CD11c⁺CFSE⁺ migratory DCs was significantly higher in the BTC-vaccinated groups than in the RTC groups across 4T1 and MC38 tumor models (Figs. 2A and EV3B). Of note, the group that received only BLys adjuvant showed higher counts of CD45⁺CD11c⁺CFSE⁺ DCs compared to both the BTC and RTC groups (Fig. 2A). This is because CD11c is a marker for all DCs and is not antigen-specific. BLys, a BCG lysate, is highly phagocytosed by DCs and has a strong ability to induce DC maturation. Therefore, the significant increase in CD45⁺CD11c⁺CFSE⁺ DC counts in the CFSE-labeled BLys group mainly reflects its innate capacity to induce DC maturation and its ability to migrate more effectively into DLNs. In summary, our findings confirm that BTCs, on which tumor cells were coated with BCG lysate, significantly enhance DC recognition and uptake of tumor cell-derived antigens, thereby promoting efficient DC migration to DLNs for subsequent antigen presentation.

We next investigated the regulatory effects of BTC vaccination on CD8⁺ T cell subset remodeling in DLNs using flow cytometry to analyze different immune CD8^+^ cell subtypes, as shown in the gating strategy in Fig. EV3A. Consistent with enhanced antigen presentation, BTC immunization significantly increased the abundance of tumor-specific tetramer-positive CD8⁺ T cells (Fig. 1N–Q), and CD44⁺ memory CD8⁺ T cells (Figs. 2B and EV3C). For exhausted CD8⁺ T cell subsets, BTC vaccination upregulated the proportion of TCF1⁺CD44⁺ progenitor exhausted T cells (Figs. 2C and EV3D) and markedly reduced the frequency of TIM-3⁺PD-1⁺ terminally exhausted T cells (Figs. 2D and EV3E). These findings indicate that BTC immunization remodels the DLN immune microenvironment, expands tumor-specific memory CD8⁺ T cell populations, and reverses the terminally exhausted state of anti-tumor CD8⁺ T cells.

### BTC vaccination leads to robust anti-tumor responses with few adverse reactions

**Figure EV4:**
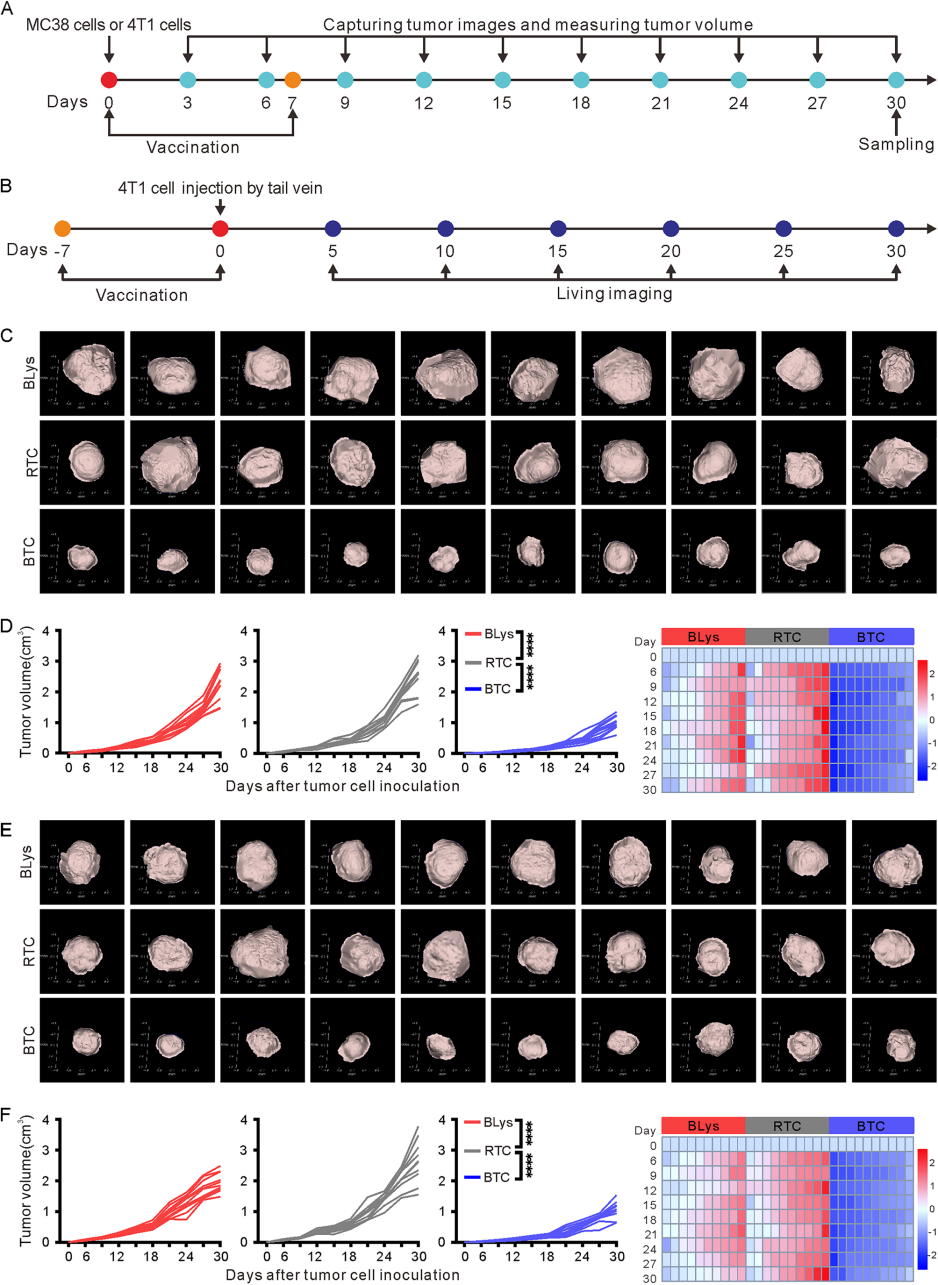
BTC vaccination results in strong anti-tumor reactions, related to Fig. 3. (**A**) Schematic presentation of the animal experimental protocol related to Fig. 3A. (**B**) Schematic presentation of the metastatic experimental protocol related to Fig. 3E. (**C**, **D**) The tumor images of the MC38 model on day 21 after tumor cell injection (**C**), and the tumor growth curve and heatmap presentation (**D**). (**E**, **F**) The tumor images of the CT26 model on day 21 after tumor cell injection (**E**), and the tumor growth curve and heatmap presentation (**F**). Data are presented as mean ± SD (*N* = 10 samples). Two-way ANOVA with Tukey multiple comparisons: ****P < 0.0001.

**Figure 3:**
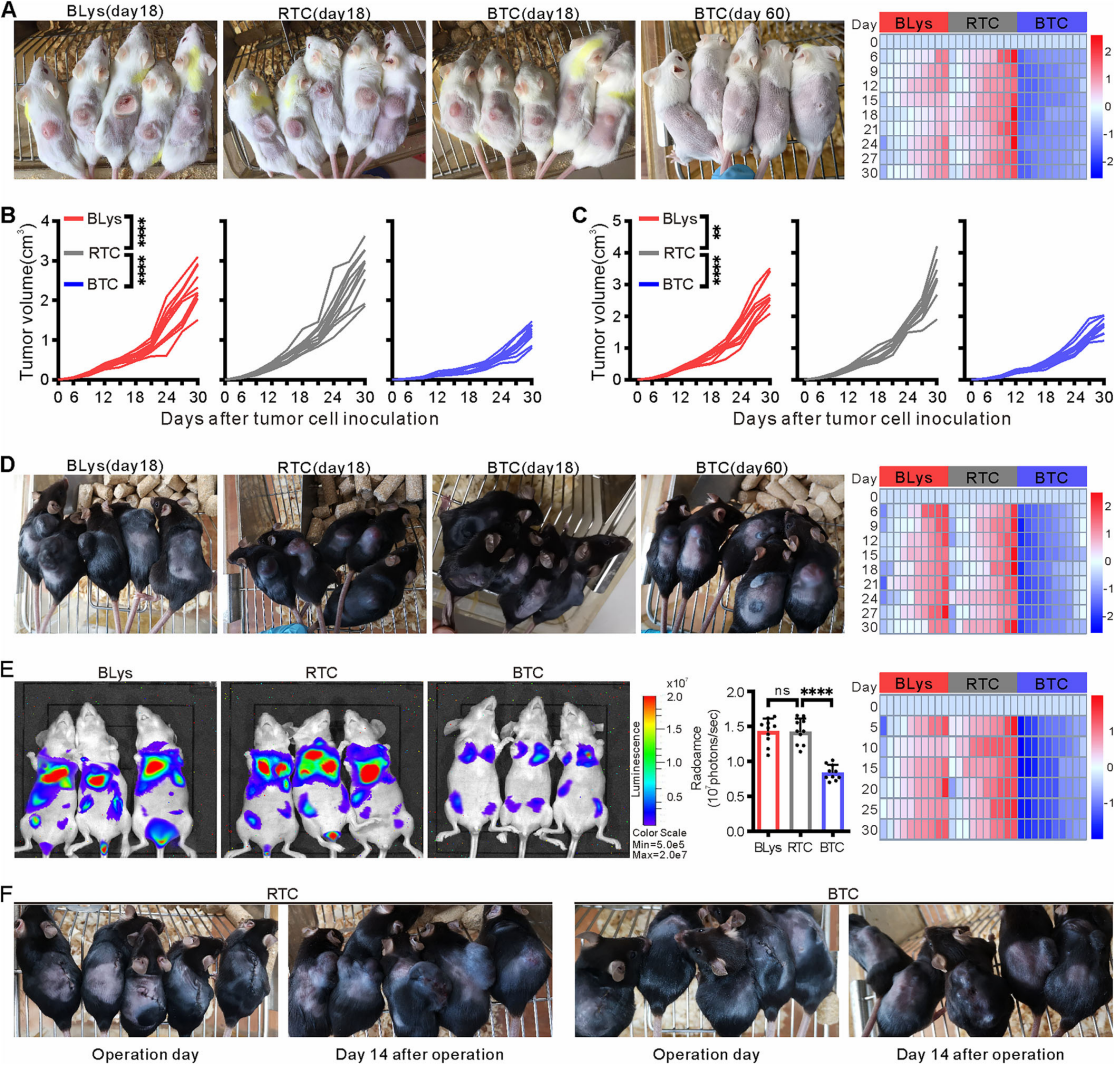
BTC vaccination results in strong anti-tumor reactions. Tumor models were established in syngeneic mice with MC38, B16-F10, CT26, and LL/2 tumor cells. A metastatic model was developed using 4T1 cells. Tumor growth was monitored every three days, and pulmonary metastasis of 4T1 cells was evaluated using small animal imaging systems every five days. (**A**, **B**) The representative tumor morphology (**A**, left) on the 18th day after tumor cell injection in the MC38 model, and the heatmap (**A**, right) and tumor curve (**B**) illustrate the relative tumor volume for each mouse at every measurement timepoint (*n* = 10), ****P < 0.0001. (**C**, **D**) The representative tumor morphology (**D**, left) on the 18th day after tumor cell injection in the B16-F10 model, and the heatmap (**D**, right) and tumor curve (**C**) illustrate the relative tumor volume for each mouse at every measurement timepoint (*n* = 10), **P = 0.0096 and ****P < 0.0001. (**E**) The representative image (left) and metastatic level on the 21st day (middle) in the 4T1 model. A heatmap (right) showing the relative metastasis for each mouse at every measurement time point (*n* = 10), ****P < 0.0001. (**F**) The tumor mass was surgically excised in the B16-F10 model to assess tumor recurrence following RTC or BTC vaccination (*n* = 5). Data are presented as mean ± SD. Two-way ANOVA with Tukey multiple comparisons: **P < 0.01, ****P < 0.0001, NS: non-significant. See also Figs. EV4 and 5. Source data are available online for this figure.

The universal anti-tumor efficacy of BTC vaccines was validated in five immunocompetent syngeneic tumor models (B16-F10, MC38, CT26, 4T1, and LL/2). The detailed schedules of tumor inoculation, vaccine administration, and sample collection are summarized in Fig. EV4A,B. In the established subcutaneous tumor models, BTC vaccination induced prominent tumor growth suppression, with markedly reduced tumor volume and burden compared to the RTC and BLys control groups (Fig. 3A,D). Mice in the RTC and BLys groups required early euthanasia due to excessive tumor growth or ulceration within 30 days, whereas BTC-vaccinated mice achieved long-term survival up to 60 days, with complete tumor regression observed in a subset of individuals (right panel in Fig. 3A,D). Tumor growth heatmaps (Figs. 3A,D and EV4D,F, right) and individual growth curves (Figs. 3B,C and EV4D,F) confirmed no significant therapeutic difference between the RTC and BLys groups, while BTC treatment conferred consistent and statistically significant tumor inhibition across all tested tumor models. In the 4T1 lung metastasis model, BTC vaccination efficiently reduced pulmonary metastatic lesion formation, whereas prominent metastatic dissemination was detected in RTC and BLys immunized mice (Fig. 3E). Furthermore, in a surgical tumor recurrence model, postoperative BTC vaccination effectively delayed tumor relapse and restricted recurrent tumor progression (Fig. 3F), highlighting its potential for adjuvant immunotherapy. Taken together, these findings suggest that BTC vaccination leads to the production of a robust anti-tumor activity.

**Figure EV5:**
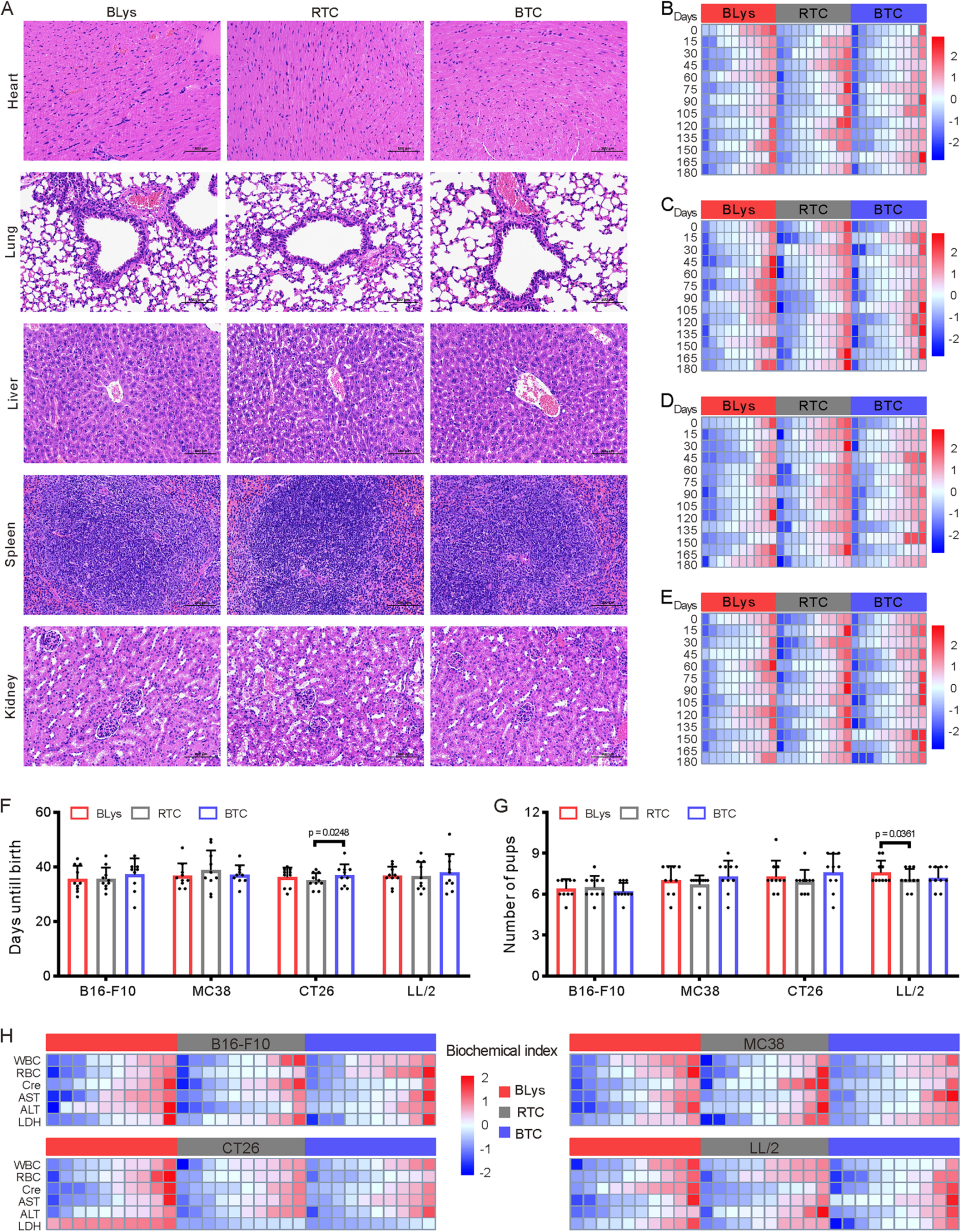
BTC vaccination does not cause significant adverse reactions, related to Fig. 3. (**A**) The heart, lung, liver, spleen, and kidney tissues of the mice that were administered vaccinations of BLys, RTC, or BTC were subjected to staining with H&E. (**B**–**E**) Heatmaps illustrating the body weight of each mouse on every observed day in syngeneic mice vaccinated with B16-F10 (**B**), MC38 (**C**), CT26 (**D**), or LL/2 (**E**). (**F**) There was no significant variation in the mean time to first parturition among the syngeneic mice vaccinated with BLys, RTC, or BTC. (**G**) The average number of pups born did not exhibit any significant variances among the syngeneic mice that received vaccinations of BLys, RTC, or BTC. (**H**) Heatmaps illustrate the relative levels of peripheral blood WBC and RBC counts, as well as the biochemical indicators Cre, AST, ALT, and LDH, in syngeneic mice that were vaccinated with BLys, RTC, or BTC. Data are presented as mean ± SD (*N* = 10 samples). Two-way ANOVA with Tukey multiple comparisons.

To systematically evaluate the biosafety of BTC vaccines, long-term toxicity assessment was performed in tumor-naive mice receiving continuous B16-F10, MC38, CT26 or LL/2 BTC immunization. No obvious abnormalities in body weight, fur condition, daily activity, or reproductive capacity were observed across all experimental groups. Histopathological H&E staining of major visceral organs (heart, liver, spleen, lung, and kidney) revealed no inflammatory infiltration or tissue damage in BTC-vaccinated mice (Fig. EV5A). Body weight comparisons also did not show any statistically significant differences between the different groups of vaccinated mice (Fig. EV5B–E). Reproductive indicators such as age at first reproduction (Fig. EV5F) and average litter size (Fig. EV5G) were similar among mice vaccinated with the four different tumor model vaccines. Blood parameters including WBCs, RBCs, Cre, AST, ALT, and LDH also did not vary significantly among the vaccinated mice (Fig. EV5H). In summary, BTC vaccination exhibits negligible systemic adverse effects and satisfactory in vivo biosafety.

### BTC vaccination elicits tumor-specific cellular immunity

**Figure 4:**
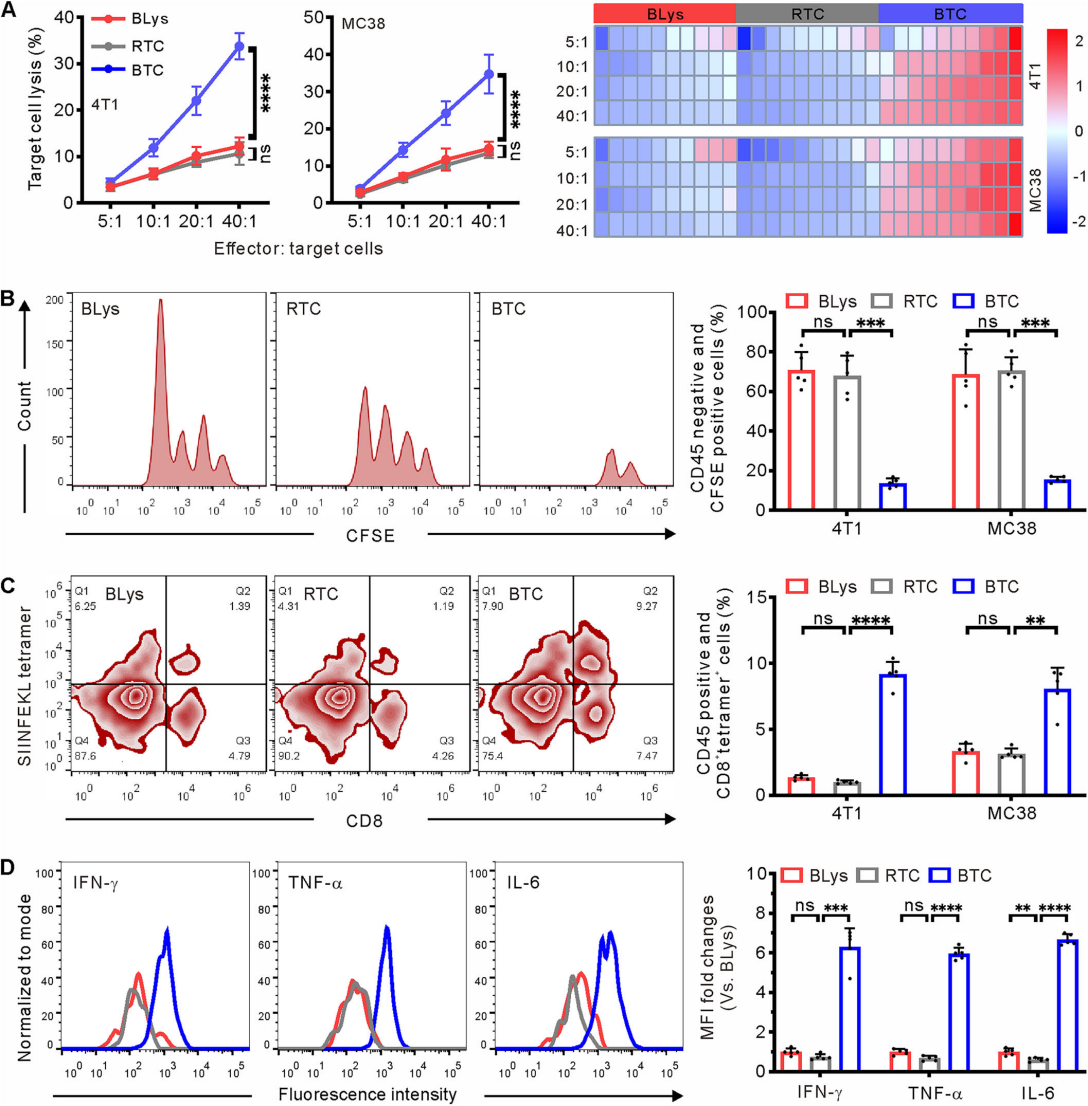
BTC vaccination results in the development of antigen-specific cellular immunity. (**A**) 4T1 or MC38 cells were used as target cells, while splenocytes from mice vaccinated with BLys, RTC, or BTC were used as effector cells. Cell lysis levels were assessed through an in vitro cytotoxicity assay. The heatmap illustrates the intensity of various effector and target ratios (*n* = 10), ****P < 0.0001. (**B**) CFSE-labeled 4T1 or MC38 cells were injected intraperitoneally, and the abdominal cells were gathered 24 h post-injection. Subsequently, they were assessed via flow cytometry to determine the proliferation rate and the proportion of CD45^-^CFSE^+^ tumor cells (*n* = 5), ***P = 0.0007 (4T1) and ***P = 0.0002. (**C**) CD45^+^ immune cells were gated out from the tumor tissues of the 4T1 or MC38 models and then examined for CD8^+^Tetramer^+^ lymphocytes using flow cytometry (*n* = 5), **P = 0.0061 and ****P < 0.0001. (**D**) CD45^+^CD8^+^Tetramer^+^ lymphocytes isolated from tumor masses of syngeneic mice (4T1) that were immunized with BLys, RTC, or BTC were subsequently assessed for their levels of secretion of IFN-γ, TNF-α, and IL-6 (*n* = 5), **P = 0.0035, ***P = 0.0004 and ****P < 0.0001. Data are presented as mean ± SD. Two-way ANOVA with Tukey multiple comparisons: **P < 0.01, ***P < 0.001, ****P < 0.0001, NS: non-significant. See also Fig. EV6. Source data are available online for this figure.

**Figure EV6:**
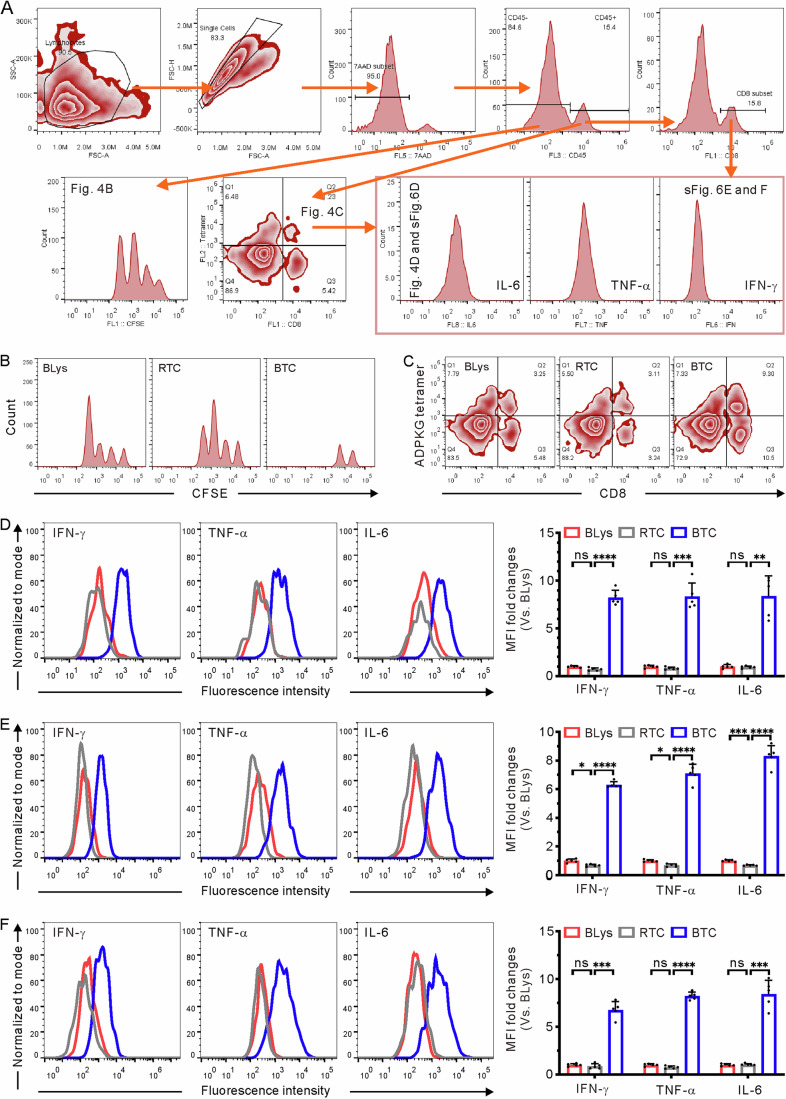
BTC vaccination results in the generation of antigen-specific cellular immunity, related to Fig. 4. (**A**) The gating strategy for flow cytometry analysis in Fig. 4. Debris and duplicate cells were excluded first, followed by gating out the 7-AAD-negative and CD45-positive cells. These cells were then used to analyze cell proliferation generations (CFSE-positive cells), CD8 and tetramer double-positive cells, which were used to analyze the IL-6, TNF-α, or IFN-γ-positive cells. The CD8-positive cells were also used to analyze the IL-6, TNF-α, or IFN-γ-positive cells. (**B**) Representative images of flow cytometry analysis (MC38) related to Fig. 4B. (**C**) Representative images of flow cytometry analysis (MC38) related to Fig. 4C. (**D**) CD45^+^CD8^+^Tetramer^+^ lymphocytes isolated from tumor masses of syngeneic mice (MC38) that were immunized with BLys, RTC, or BTC were subsequently assessed for their levels of secretion of IFN-γ, TNF-α, and IL-6. (**E**, **F**) Non-antigen-specific CD45^+^CD8^+^ cells isolated from tumor masses of syngeneic mice 4T1 (**E**) or MC38 (**F**) that were immunized with BLys, RTC, or BTC were subsequently assessed for their levels of secretion of IFN-γ, TNF-α, and IL-6. Data are presented as mean ± SD (*N* = 5 independent experiments). Two-way ANOVA with Tukey multiple comparisons: *P < 0.05, **P < 0.01, ***P < 0.001, ****P < 0.0001, NS: non-significant.

To quantify tumor-specific cellular cytotoxicity induced by BTC vaccination, an LDH release assay was performed using splenocytes as effector cells. Splenocytes isolated from BTC-immunized mice exhibited significantly enhanced cytolytic activity against 4T1 and MC38 target tumor cells, while the RTC and BLys groups displayed limited cytotoxicity with no statistical difference between controls (Fig. 4A). CFSE-labeled 4T1 or MC38 tumor cells were injected into the peritoneal cavity of immunized mice. Flow cytometry analysis of peritoneal cells (gating strategy shown in Fig. EV6A) after 24 h revealed fewer CD45^-^CFSE^+^ tumor cells in mice vaccinated with BTC compared to those vaccinated with BLys or RTC (Fig. 4B, right). Moreover, tumor cells in mice vaccinated with BLys or RTC underwent four generations of cell division, whereas those in BTC-vaccinated mice underwent only two generations of cell division (Figs. 4B and  EV6B). For functional analysis of tumor-specific CD8⁺ T cells, neoantigen tetramer staining and intracellular cytokine detection were performed. BTC vaccination significantly expanded neoantigen-specific tetramer-positive CD8⁺ T cells (Figs. 4C and EV6C) and promoted robust secretion of the anti-tumor cytokines IFN-γ, TNF-α, and IL-6 (Figs. 4D and EV6D). In addition, non-antigen-specific bystander CD8⁺ T cells from BTC-vaccinated mice also showed elevated expression of inflammatory cytokines (Fig. EV6E,F). These findings indicate that BTC vaccination not only enhances the immune killing activity of tumor-specific CD8^+^ cells, but also non-specifically boosts the immune activity of other CD8^+^ cells.

### BTC vaccination induces tumor-specific humoral immunity and synergistically inhibits tumor growth with cellular immunity

**Figure 5:**
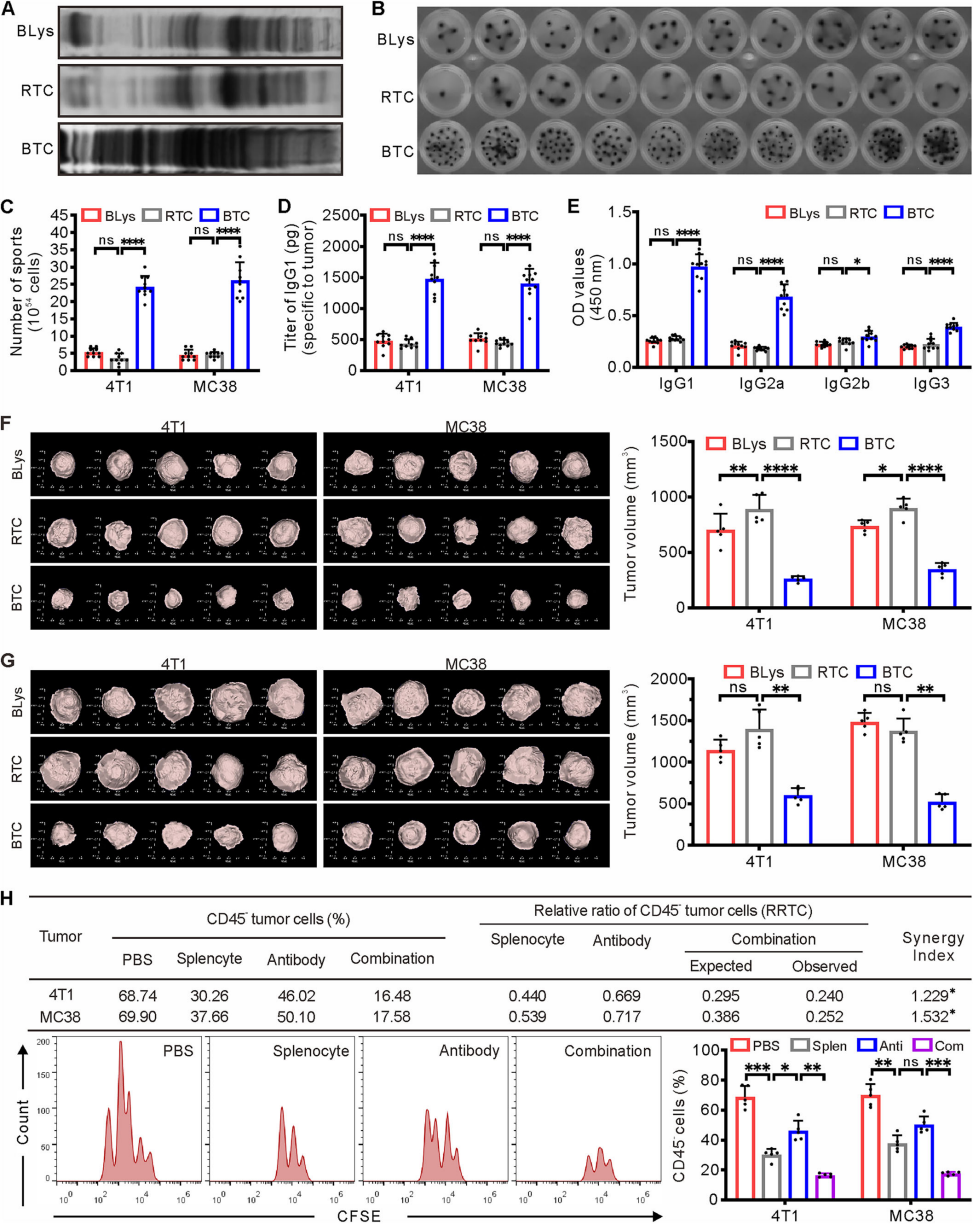
BTC vaccination elicits antigen-specific humoral immunity and promotes synergy between cellular and humoral immunity. (**A**) Tumor-specific IgG in the serum from mice vaccinated with BLys, RTC, or BTC was detected by western blot (*n* = 3). (**B**, **C**) The splenocytes produced IgG antibodies targeting 4T1 or MC38 cells (**B**), with an average of IgG-secreting cells per 10^3^ splenocytes (**C**) were detected by ELISPOT (*n* = 10), ****P < 0.0001. (**D**, **E**) The levels of IgG (**D**) and IgG subsets (**E**) in the serum of mice vaccinated with BLys, RTC, or BTC were assessed by ELISA (*n* = 5), *P = 0.0415 and ****P < 0.0001. (**F**) The images (left) show tumor masses of 4T1-bearing mice, and the tumor volume of mice vaccinated with BLys, RTC, or BTC on day 18 after tumor cell inoculation and adaptive transfer of splenocytes (right, *n* = 5), *P = 0.0174, **P = 0.0078 and ****P < 0.0001. (**G**) The images (left) show tumor masses of 4T1-bearing mice, and the tumor volume of mice vaccinated with BLys, RTC, or BTC on day 18 after tumor cell inoculation and adaptive transfer of antibodies (right, *n* = 5), **P = 0.0064 (4T1) and **P = 0.0020. (**H**) CFSE-labeled 4T1 or MC38 cells were injected into the abdominal cavity of their respective syngeneic mice. Subsequently, the cells were exposed to splenocytes and antibodies derived from mice immunized with BTC alone or in combination. Flow cytometric analysis was performed to evaluate the proliferation of CD45^−^ 4T1 or MC38 cells and establish the syngeneic index (*n* = 5), *P < 0.0169, **P < 0.0027 (4T1), **P = 0.0016, ***P = 0.0002 (4T1) and ***P = 0.0008. Data are presented as mean ± SD. Two-way ANOVA with Tukey multiple comparisons: *P < 0.05, **P < 0.01, ***P < 0.001, ****P < 0.0001, NS: non-significant. See also Fig. EV7. Source data are available online for this figure.

**Figure EV7:**
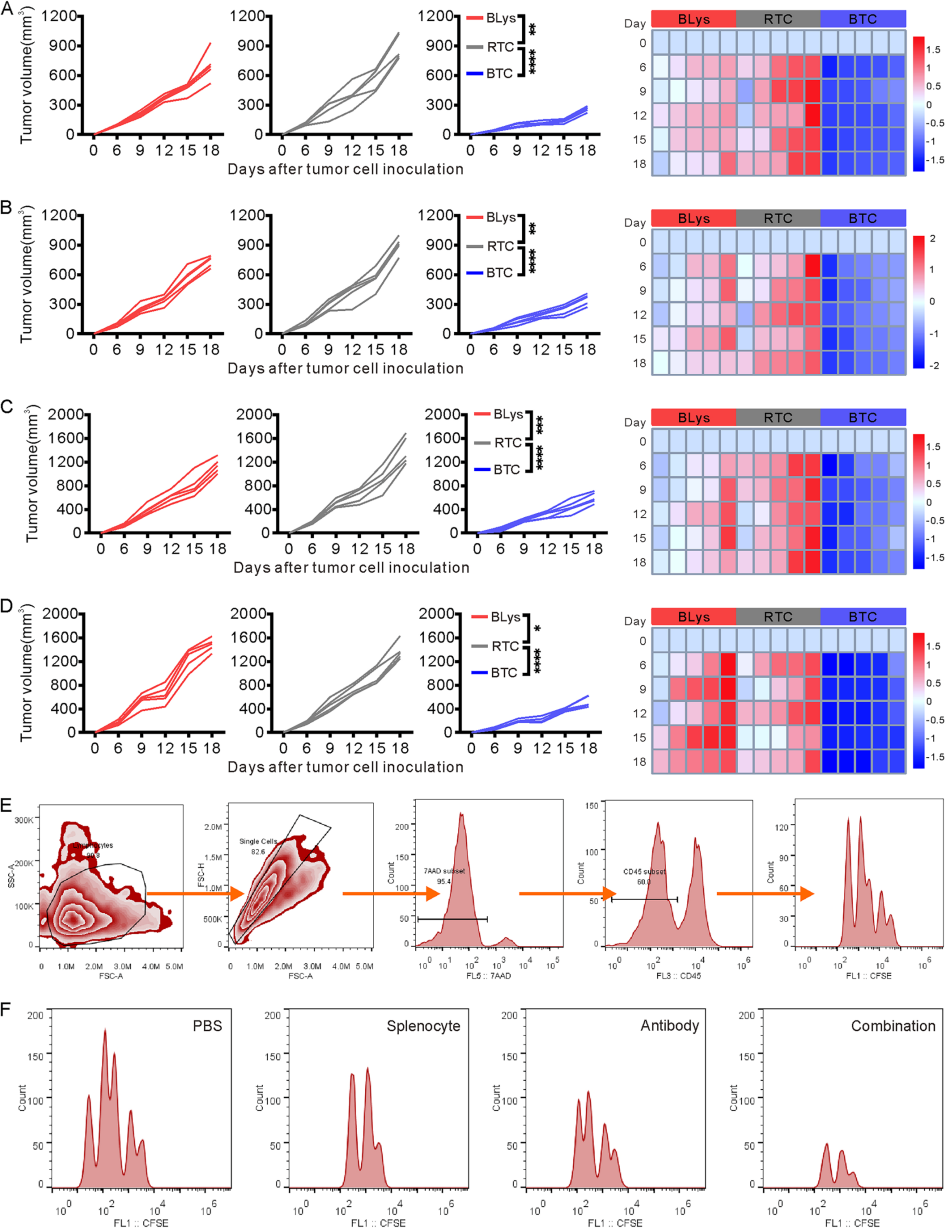
Combination of cellular and humoral immunity results in synergistic anti-tumor effects, related to Fig. 5. (**A**, **B**) The tumor growth curve and heatmap visualization of tumor volume in 4T1 (**A**) or MC38 (**B**) model mice treated with lymphocytes from syngeneic mice vaccinated with BLys, RTC, or BTC. (**C**, **D**) The tumor growth curve and heatmap visualization of tumor volume in 4T1 (**C**) or MC38 (**D**) model mice treated with antibodies from syngeneic mice vaccinated with BLys, RTC, or BTC. (**E**) The gating strategy for flow cytometry analysis in Fig. 5H. Debris and duplicate cells were excluded first, followed by gating out the 7-AAD-negative and CD45-positive cells, which were used to analyze cell proliferation generations (CFSE-positive cells). (**F**) Representative images of flow cytometry analysis (MC38) related to Fig. 5H. Data are presented as mean ± SD (*N* = 5 samples). Two-way ANOVA with Tukey multiple comparisons: *P < 0.05, **P < 0.01, ****P < 0.0001.

In addition to cellular immunity, BTC vaccination effectively activated tumor-specific humoral immune responses. Western blot and ELISA analyses revealed elevated levels of tumor-specific IgG in the serum of BTC-immunized mice (Fig. 5A,D), while ELISPOT assays confirmed increased frequencies of tumor-specific antibody-secreting lymphocytes in the spleen (Fig. 5B,C). IgG subtype analysis demonstrated significant upregulation of both IgG1 and IgG2a, indicating concurrent Th1 and Th2 immune activation (Fig. 5E). Adoptive transfer experiments dissected the individual and combined contributions of cellular and humoral immunity to BTC-mediated anti-tumor effects. Tumor-bearing mice receiving splenocytes from BTC-vaccinated donors showed remarkable tumor growth inhibition (Figs. 5F and EV7A,B), while the passive transfer of BTC-induced tumor-specific antibodies conferred moderate anti-tumor protection (Figs. 5G and EV7C,D). Notably, cellular immunity demonstrated superior therapeutic potency compared to antibody-mediated humoral immunity. The combined adoptive transfer of splenocytes and antibodies produced the strongest inhibition of in vivo tumor cell proliferation, with synergistic index values exceeding 1.0 in both 4T1 and MC38 models (Figs. 5H and EV7E,F). These findings demonstrate that BTC vaccination induces potent humoral immunity and that both cellular and humoral immune responses act synergistically to suppress tumor progression.

### BTC vaccination revitalizes the immunosuppressive tumor microenvironment

**Figure EV8:**
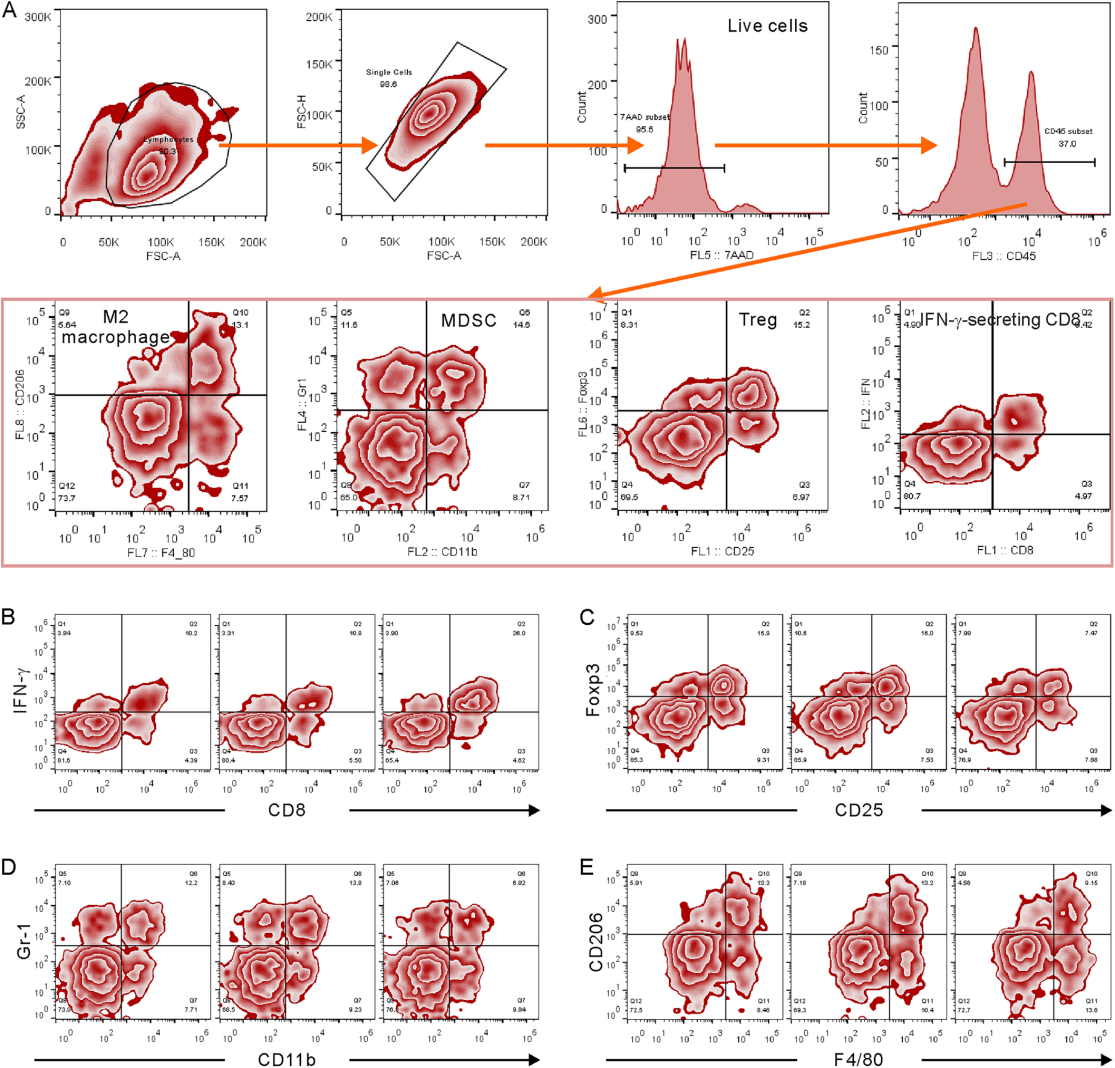
BTC vaccination does not cause significant adverse reactions, related to Fig. 6. (**A**) The gating strategy for flow cytometry analysis in Fig. 6. Debris and duplicate cells were excluded first, followed by gating out the 7-AAD-negative and CD45-positive cells. These cells were then used to analyze double-positive cells for F4-80 and CD206, CD11b and Gr1, CD25 and Foxp3, or CD8 and TNF-γ. (**B**) Representative images of flow cytometry analysis (MC38) related to Fig. 2A. (**C**) Representative images of flow cytometry analysis (MC38) related to Fig. 6B. (**D**) Representative images of flow cytometry analysis (MC38) related to Fig. 6C. (**E**) Representative images of flow cytometry analysis (MC38) related to Fig. 6D.

**Figure 6:**
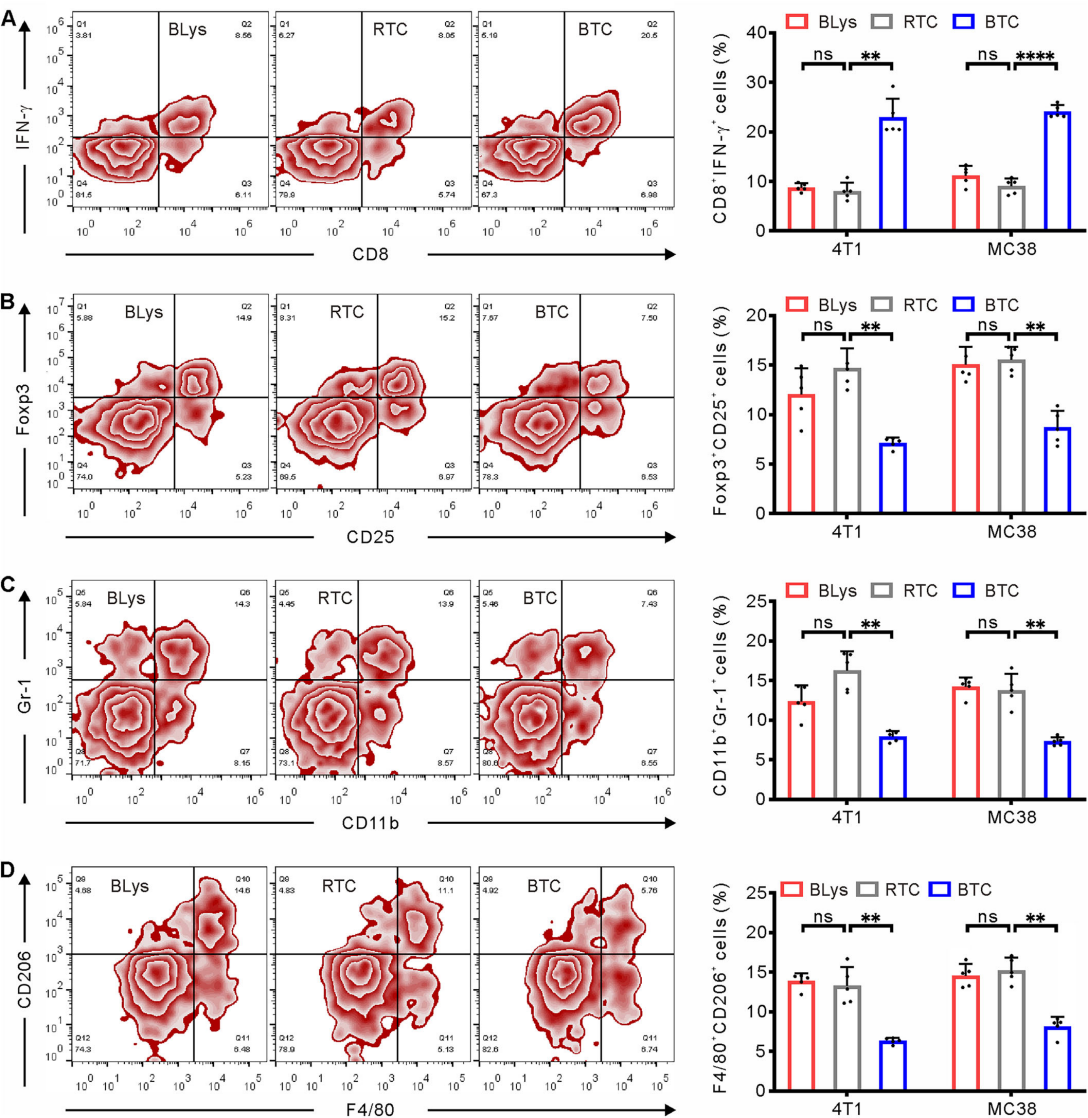
BTC vaccination mitigates immunosuppressive mechanisms within the tumor microenvironment. (**A**–**D**) CD45^+^ immune cells were gated out from the single cells obtained from tumor tissues of mice that were vaccinated with BLys, RTC, or BTC. These cells were then analyzed using flow cytometry. Subsequently, the populations of IFN-γ-secreting CD8 cells (CD45^+^ IFN-γ^+^CD8^+^), Tregs (CD45^+^CD25^+^Foxp3^+^), MDSCs (CD45^+^CD11b^+^Gr-1^+^), and M2-type TAMs (CD45^+^F4/80^+^CD206^+^) were analyzed (*n* = 5). The percentages and representative images of IFN-γ-secreting CD8 cells (**A**, **P = 0.0017 and ****P < 0.0001); Tregs (**B**, **P = 0.0012 (4T1) and **P = 0.0015); MDSCs (**C**, **P < 0.0035 (4T1) and **P = 0.0039), and M2-type TAMs (**D**, **P < 0.0040 (4T1) and **P = 0.0075) are displayed. Data are presented as mean ± SD. Two-way ANOVA with Tukey multiple comparisons: *P < 0.05, **P < 0.01, ***P < 0.001, ****P < 0.0001, NS: non-significant. See also Fig. EV8. Source data are available online for this figure.

The tumor microenvironment (TME) is characterized by extensive immunosuppression, driven by multiple inhibitory cell subsets including regulatory T cells (Tregs), myeloid-derived suppressor cells (MDSCs), and M2-polarized tumor-associated macrophages (TAMs). These immunosuppressive populations collectively impair the infiltration and function of cytotoxic CD8⁺ T cells (Baharom et al, 2022). Flow cytometry analysis of tumor-infiltrating immune cells (gating strategy shown in Fig. EV8A) demonstrated that BTC vaccination significantly increased the intratumoral proportion of functional IFN-γ⁺ CD8⁺ T cells (Figs. 6A and EV8B). In parallel, BTC immunization markedly reduced the abundance of immunosuppressive cell subsets, including Foxp3⁺CD25⁺ Tregs, CD11b⁺Gr-1⁺ MDSCs, and F4/80⁺CD206⁺ M2-type TAMs (Figs. 6B–D and EV8C–E). These results demonstrate that BTC vaccination efficiently reverses TME immunosuppression, reshapes the intratumoral immune landscape towards an anti-tumor phenotype, and provides a favorable microenvironment for sustained anti-tumor immune attack.

### Synergistic anti-tumor effects of BTC vaccination and anti-PD-1 antibody therapy

**Figure 7:**
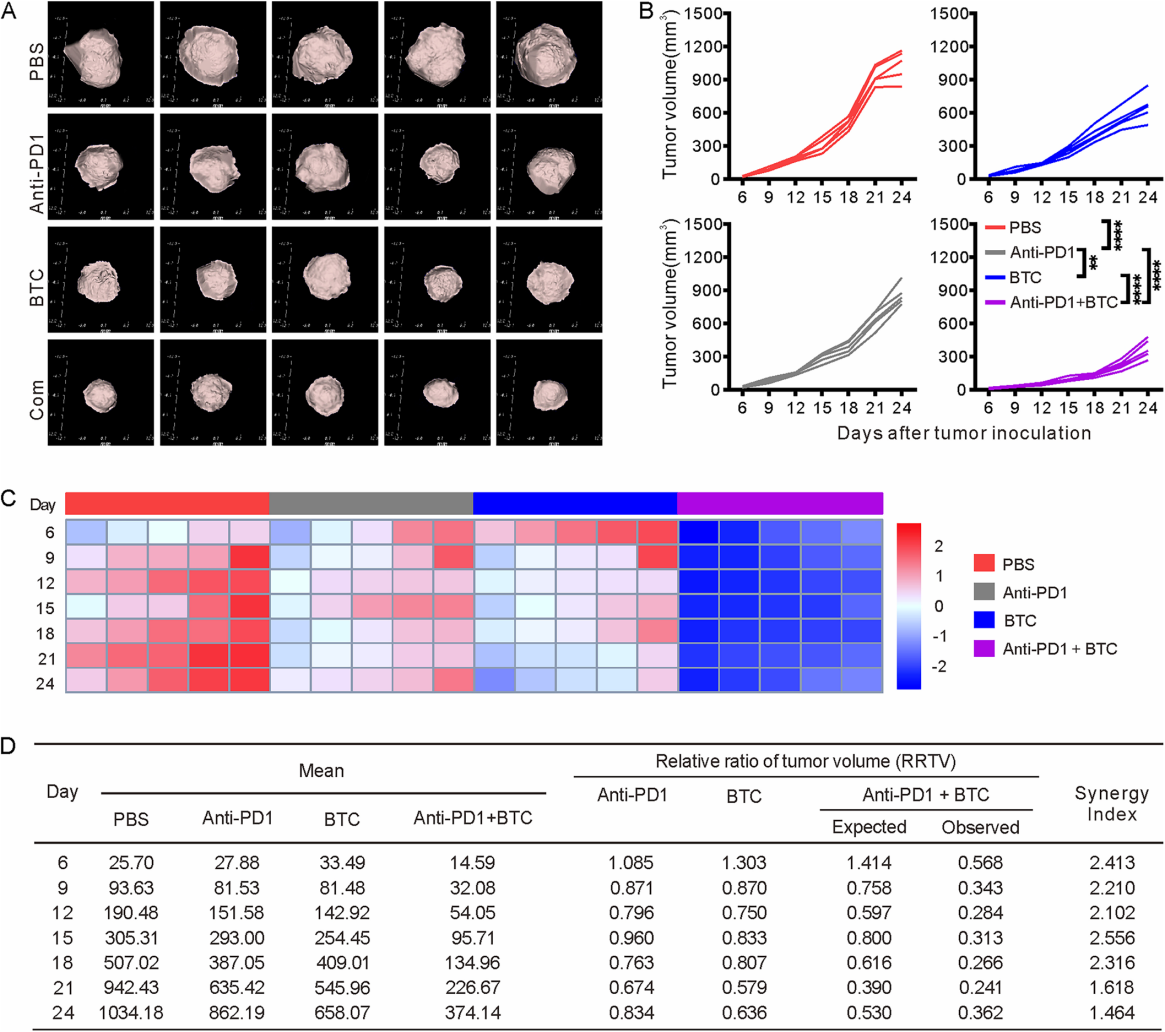
Combination of anti-PD-1 therapy with BTC vaccination results in a synergistic enhancement of the anti-tumor effects. 4T1-bearing mice were treated with PBS, anti-PD-1 antibody, BTC vaccination, or combination of anti-PD-1 antibody with BTC vaccination. (**A**) Tumor images of the mice that received the specified treatment were taken on Day 18 following tumor cell inoculation (*n* = 5). (**B**) The tumor growth curves of each mouse (*n* = 5), **P = 0.0094 and ****P < 0.0001. (**C**) The heatmap shows the relative changes in tumor volume over time for each mouse (*n* = 5). (**D**) The mean tumor volume of each group was used to calculate the synergy index. The relative ratio of tumor volume (RRTV) for the anti-PD-1 group, the BTC group, and the anti-PD-1^+^ BTC group (observed) was determined by dividing their mean values by the mean value of the PBS group. The expected RRTV for the anti-PD-1 + BTC group was calculated by multiplying the RRTV of the anti-PD-1 group by the BTC group. The synergy index was then computed by dividing the expected RRTV of the anti-PD-1 + BTC group by its observed RRTV. A synergy index value greater than 1 indicates a synergistic anti-tumor effect (*n* = 5). Data are presented as mean ± SD. Two-way ANOVA with Tukey multiple comparisons: **P < 0.01, ****P < 0.0001. Source data are available online for this figure.

Given the favorable immunomodulatory properties of BTC vaccines, we further explored their potential for combination with immune checkpoint blockade therapy in the 4T1 breast cancer model. Both single BTC vaccination and anti-PD-1 monotherapy significantly delayed tumor growth compared to the PBS control group, while the combined treatment of BTC vaccination plus anti-PD-1 antibody achieved the most potent therapeutic efficacy, with significant differences in tumor volume observed across all experimental groups (Fig. 7A–C). Calculation of the synergy index confirmed robust synergistic anti-tumor effects in the combination group, with most index values higher than 2.0 throughout the treatment period (Fig. 7D). These data collectively indicate that BTC vaccination sensitizes tumors to anti-PD-1 immunotherapy, and the dual targeting of tumor antigens and immune checkpoint signaling generates complementary and synergistic therapeutic benefits.

## Discussion

Immunotherapy, particularly tumor vaccines, is widely recognized as the fourth major therapeutic modality for cancer, following surgery, chemotherapy, and radiation therapy. Tumor immunotherapeutic approaches are highly diverse and can be categorized into passive and active immunity (Zhang et al, 2025). Passive immunity involves the administration of tumor-specific antibodies, cytokines, and adoptively transferred immunoreactive cells, whereas active immunity encompasses a range of strategies, including protein or peptide vaccines targeting tumor-specific or tumor-associated antigens, DC vaccines, allogeneic or xenogeneic tumor whole-cell vaccines, immune checkpoint inhibitors, phototherapy, and other novel methods (Meng et al, 2021). WTCVs play a pivotal role in tumor immunotherapy research owing to their unique advantages (Diao and Liu, 2023). In contrast to single tumor-specific or associated antigen vaccines, WTCVs contain a comprehensive repertoire of all known and unknown tumor-specific and tumor-associated antigen components, which can be recognized by both the innate and adaptive immune systems (Dranoff et al, 1993; Liu et al, 2013; Stagg et al, 2004; Wang et al, 2025). They are capable of preventing tumor immune escape and carry individual-specific tumor antigens as well as major histocompatibility complexes (MHC), rendering them easily recognizable by the host immune system. Moreover, WTCVs can present antigens through both MHC class I and MHC class II pathways, thereby triggering robust tumor-specific immune responses and long-lasting CD8^+^ T cell memory. Additionally, WTCVs can be modified and enhanced via genetic engineering techniques to effectively boost their immune response activity (Dranoff et al, 1993; Liu et al, 2013; Stagg et al, 2004; Wang et al, 2025), underscoring their considerable potential for clinical application. A critical step in immune recognition is the ability of the immune system to identify danger signals, which include PAMPs derived from invading pathogens and damage-associated molecular patterns released by abnormal cells. Current vaccine strategies targeting bacteria, viruses, and other pathogenic microorganisms leverage PAMP signals to effectively activate the immune system (Koch and Mizrahi, 2018). In the present study, lysates of Mycobacterium tuberculosis (MTb) were chemically modified to carry a positive charge and subsequently coated onto the surface of irradiated tumor cells, resulting in the generation of BTCs as a novel model vaccine. Our experimental results demonstrated that BTCs are more readily recognized and processed by DCs, leading to enhanced cross-presentation of tumor cell-specific antigens to CD8^+^ lymphocytes. In five distinct mouse tumor models (B16-F10, MC38, CT26, 4T1, and LL/2), BTC vaccination effectively suppressed tumor growth and metastasis while eliciting potent tumor-specific cellular and humoral immune responses. Further mechanistic investigations revealed that BTC immunization can successfully remodel the immunosuppressive microenvironment in local lymph nodes and tumor tissues, expand the population of tumor-specific memory CD8^+^ T cells, and reduce the frequency of terminally exhausted CD8^+^ T cells. Furthermore, combining BTC vaccination with anti-PD-1 antibody therapy exhibited significant synergistic anti-tumor effects. Collectively, these findings indicate that BTCs represent a novel type of tumor whole-cell vaccine with promising clinical potential, which warrants further preclinical and clinical investigations.

Over the past decade, extensive research has been conducted to explore various strategies for enhancing the induction of tumor-specific immune responses by tumor cell vaccines. In 1993, Dranoff et al first reported the development of whole-tumor cell vaccines by engineering melanoma cells to express granulocyte-macrophage colony-stimulating factor (GM-CSF) (Dranoff et al, 1993). In a mouse model, this approach effectively induced DC migration, differentiation, and maturation, facilitating tumor antigen processing and the initiation of tumor-specific immune responses. Subsequent studies have predominantly focused on genetically modified tumor cell strategies, with an emphasis on enhancing the anti-tumor activity of cytokines through membrane-bound expression (Hodi and Dranoff, 1998). The juxtacrine cytokine delivery method, which occurs during direct cell-to-cell interaction, may minimize nonspecific immune cell activation (Blagovic et al, 2013). A variety of cytokines, including GM-CSF, FMS-like tyrosine kinase 3 ligand, and interleukins (IL-2, IL-4, IL-9, and IL-12), have been expressed on the cell surface to augment anti-tumor effects (Komuczki et al, 2019; Read et al, 2016). In the current study, we propose a novel approach for constructing tumor cell vaccines by coating the surface of tumor cells with bacterial components. This approach differs from the well-established genetic engineering strategies and effectively stimulates tumor-specific immune responses by “tricking” the immune system into recognizing tumor cells as bacterial pathogens. This enhances the activity of immune cells, including antigen-presenting cells that phagocytose bacterialized tumor cells for antigen processing and cross-presentation. Importantly, our BTCs retain their original tumor specificity and can be combined with existing therapeutic approaches to develop innovative tumor cell vaccines.

The immune system effectively recognizes pathogens through pathogen-associated molecular patterns (PAMPs), such as CpG oligodeoxynucleotides and surface polysaccharide derivatives. These conserved molecular structures enable the host immune system to distinguish between “nonself” and “self,” thereby activating adaptive immunity. This understanding has facilitated the utilization of PAMPs in vaccine development to enhance immune responses. However, the complex nature of the immune system and in vivo environments can lead to unpredictable outcomes, particularly when multiple PAMP elements are combined. Currently, research has found that Mycobacterium tuberculosis (MTb) is the pathogen of human tuberculosis, a bacterium that grows intracellularly and mainly resides in mononuclear cells, primarily macrophages and dendritic cells (DC) after entering the human body (Koch and Mizrahi, 2018). In addition to pathogen-associated molecular patterns (PAMPs), MTb has evolved a complex set of adhesion molecules (Squeglia et al, 2018). These adhesion molecules on MTb can specifically bind to corresponding receptors on the surface of host immune cells, leading to the activation of host immune cells and rapid uptake of the adhered MTb (Smith, 2003; Squeglia et al, 2018). However, once inside the host immune cells, MTb utilizes these adhesion molecules to inhibit the killing activity of immune cells, allowing it to proliferate within the infected immune cells. Studies have reported that molecules on the MTb membrane, such as Erp, Mas, FadD26, FadD28, MmpL7, FbpA, MmaA4, PcaA, and OmpA, can act as ligands, specifically adhering to corresponding receptors on macrophages (Smith, 2003; Squeglia et al, 2018). Macrophages and dendritic cells primarily come from monocytes in the bone marrow, and research has shown that MTb infects dendritic cells in a similar way to how it infects macrophages (Kim and Shin, 2022; Romero et al, 2019; Tascon et al, 2000). Previous studies have indicated that modifying Mycobacterium tuberculosis into a tumor vaccine vector, while keeping PAMPs and adhesion molecules on its surface, can boost the immune response of tumor vaccines by aiding in the engulfment, processing, and cross-presentation of tumor antigens by dendritic cells. This ultimately leads to the effective generation of tumor-specific cellular and humoral immune responses, resulting in favorable anti-tumor effects in various mouse tumor models. However, in this study, the MTb lysate was lysed and aminated to acquire a positive charge before being applied to the surface of tumor cells with a negative charge. The process of amination primarily targets the protein components of the lysate, and it was not investigated in this study whether this method of preparing tumor cells can coat the corresponding PAMPs and adhesion molecules on the tumor cell surface. However, it is believed that known PAMP molecules are complex, consisting of various protein components that could potentially be coated on the surface of bacterialized tumor cells. Additionally, PAMPs also include DNA or RNA, such as CpG, which are predominantly negatively charged molecules. Therefore, in theory, they could be coated along with the positively aminated proteins on the tumor cell surface. In conclusion, the bacterialized component in this study is quite complex, and further systematic experimental design is necessary to identify the effective components that induce immune responses. Furthermore, only one type of MTb lysate was used to bacterialize tumor cells in this study, so labeling it as “Bacterialized” may not be precise enough. Future studies should involve comparing the effects of using a variety of bacterial lysates.

In summary, in our current study, BTCs were prepared by coating aminated Mycobacterium tuberculosis lysates on the tumor cell surface. Key findings showed that BTCs can promote dendritic cell maturation and antigen cross-presentation, activate tumor-specific immunity, suppress tumor growth/metastasis in five murine models, synergize with anti-PD-1 therapy, and exhibit excellent biosafety. Thus, our bacterialization strategy provides a novel, safe approach to optimize WTCVs, holding great promise for clinical translation in cancer immunotherapy.

## Methods

**Table:** Reagents and tools table

Reagent/resource	Reference or source	Identifier or catalog number
**Experimental models**		
C57BL/6J mice (SPF grade, female, 42-56 days old)	Changsha Tianqin Biotechnology Co., Ltd., Hunan, China	Certificate No. 1107261911000088
BALB/c mice (SPF grade, female, 42-56 days old)	Changsha Tianqin Biotechnology Co., Ltd., Hunan, China	Certificate No. 1107261911000088
B16-F10 murine melanoma cell line	American Type Culture Collection (ATCC)	CRL-6475
4T1 murine breast cancer cell line	American Type Culture Collection (ATCC)	CRL-2539
CT26 murine colon cancer cell line	American Type Culture Collection (ATCC)	CRL-2638
LL/2 murine Lewis lung cancer cell line	American Type Culture Collection (ATCC)	CRL-1642
MC38 murine colon cancer cell line	China Center for Type Culture Collection (CCTCC)	CL-0241
Mycobacterium tuberculosis H37Ra	BD Biosciences	231141
**Antibodies**		
APC anti-mouse SIINFEKL-MHC I	Thermo Fisher Scientific	17-5743-82
PerCP-eFluor 710 anti-mouse MHC II	Thermo Fisher Scientific	46-0463-82
PE-eFluor 610 anti-mouse CD45	Thermo Fisher Scientific	61-0451-82
eFluor 450 anti-mouse CD8a	Thermo Fisher Scientific	48-0081-82
PE anti-mouse CD86	Thermo Fisher Scientific	12-0862-82
FITC anti-mouse CD80	Thermo Fisher Scientific	11-0801-82
APC anti-mouse CD40	Thermo Fisher Scientific	17-0401-82
PE anti-mouse CD11c	Thermo Fisher Scientific	12-0114-82
FITC anti-mouse PD-1	Thermo Fisher Scientific	11-9985-82
PE anti-mouse TIM-3	Thermo Fisher Scientific	25-5870-82
FITC anti-mouse CD44	Thermo Fisher Scientific	11-0441-82
Alexa Fluor 488 anti-mouse CD11b	Thermo Fisher Scientific	53-0112-82
APC anti-mouse Gr-1 (Ly6G/Ly6C)	Thermo Fisher Scientific	17-5931-82
FITC anti-mouse CD4	Thermo Fisher Scientific	11-0041-82
eFluor 660 anti-mouse Foxp3	Thermo Fisher Scientific	50-5773-82
PE anti-mouse CD25	Thermo Fisher Scientific	12-0251-82
PE anti-mouse CD206 (MMR)	Thermo Fisher Scientific	12-2061-82
APC anti-mouse F4/80	Thermo Fisher Scientific	17-4801-82
PE anti-mouse TCF7/TCF1	BD Biosciences	564221
Adpgk Neoepitope Tetramer (anti-PE-H-2Db)	B&M Biotech	TC-5113-1
Goat anti-mouse IgG	Abcam	ab6789
Goat anti-mouse IgG2a	Abcam	ab190463
Goat anti-mouse IgG2b	Abcam	ab210823
Goat anti-mouse IgG3	Abcam	ab97258
**Chemicals, enzymes and other reagents**		
DAPI	MedChemExpress	HY-D0814
FITC	MedChemExpress	HY-66019
DiI (1,1’-Dioctadecyl-3,3,3’,3’-tetramethylindocarbocyanine perchlorate)	Sigma-Aldrich	41085-99-8
CellTrace™ CFSE (Carboxyfluorescein succinimidyl ester)	Thermo Fisher Scientific	C34554
CellTrace™ Far Red	Thermo Fisher Scientific	34564
7-AAD Viability Staining Solution	Thermo Fisher Scientific	00-6993
DNase I	Thermo Fisher Scientific	EN0521
Fetal Bovine Serum (FBS)	Thermo Fisher Scientific	A5670701
RPMI 1640 Medium	Thermo Fisher Scientific	11875093
Penicillin-Streptomycin	Thermo Fisher Scientific	15070063
Tris-Glycine-SDS Buffer	Thermo Fisher Scientific	28362
CytoTox 96 Non-Radioactive Cytotoxicity Assay	Promega	G1780
IFN-γ ELISA Kit	Thermo Fisher Scientific	KMC4021
TNF-α ELISA Kit	Thermo Fisher Scientific	BMS607-3
IL-6 ELISA Kit	Thermo Fisher Scientific	88-7064
**Software**		
FlowJo Software v10	Tree Star, Ashland, OR, USA	Version 10 https://www.flowjo.com/
GraphPad Prism v10	GraphPad Software, San Diego, CA, USA	Version 10 https://www.graphpad.com/
R with pheatmap package	CRAN	R v4.2+ https://CRAN.R-project.org/package=pheatmap
**Other**		
RS2000Pro X-ray Irradiator	Rad Source, GA, USA	RS2000Pro
FV1000 Confocal Fluorescence Microscope	Olympus Corporation, Tokyo, Japan	FV1000
TissueLyser II	Qiagen, Germany	85300
FACSAria II Flow Cytometer	BD Biosciences, NJ, USA	FACSAria II
MT20 Live Cell Imaging System	OSIS, Germany	MT20
TM900 Tumor Measuring Device	Peira, Belgium	TM900
IVIS Lumina II In Vivo Imaging System	Caliper Life Sciences, MD, USA	IVIS Lumina II
BS-240 Vet Biochemical Analyzer	Mindray, Shenzhen, China	BS-240 Vet
ELX808IU Microplate Reader	Bio-Tek, VT, USA	ELX808IU
Mini Trans-Blot Electrophoretic Transfer Cell	Bio-Rad, Hercules, CA, USA	Mini Transblot
Patents		
US Patent for BTC preparation	USPTO	US11357843B2
Chinese Patent for BTC preparation	CNIPA	ZL201910322888.3

### Methods and protocols

#### Cell culture

The B16-F10 melanoma, 4T1 breast cancer, CT26 colon cancer, and LL/2 Lewis lung cancer cell lines were maintained for up to ten generations in RPMI 1640 or DMEM medium supplemented with 10% fetal bovine serum, 100 U/mL of penicillin, and 100 μg/mL of streptomycin. The cultures were incubated at 37 °C in a humidified 5% CO_2_ atmosphere. Reagents were applied as required for specific experimental protocols.

#### Preparation and characterization of bacterialized tumor cells

The logarithmic growth phase of Mycobacterium tuberculosis was disrupted through repeated freeze-thaw cycles and ultrasonic nebulization to extract bacterial lysate. The lysate was centrifuged at 12000 rpm for 1 min and mixed with an excess of an amino compound (e.g., ethylenediamine) solution under neutral to slightly alkaline conditions. Using 3-dimethylaminopropyl carbodiimide, the lysate was aminated with a positive charge and subsequently combined with tumor cells, attaching to the negative tumor cell surface. These bacterial lysate-modified tumor cells were termed as bacterialized tumor cells (BTCs). The BTCs were then inactivated by X-ray irradiation (80-100 Gy) with professional equipment to produce a whole-cell tumor vaccine incorporating the aminated bacterial lysate. Detailed procedures are available in patent specifications (US11357843B2 and ZL201910322888.3). To characterize these bacterialized tumor cells, we labeled the lysates obtained from bacteria with FITC, the tumor cell surface with Dil, and the cell nuclei with DAPI. Subsequently, we observed the distribution of cell fluorescence staining relative to the cell nuclei under a confocal fluorescence microscope to determine the effective coating of bacterial lysates on the surface of tumor cells. Additionally, we used scanning electron microscopy to compare the external features of normal tumor cells and bacterialized tumor cells to assess the successful coating of bacterial lysates on the surface of bacterialized tumor cells.

#### Isolation of single cells from lymph nodes and tumor tissues

Cell isolation from lymph nodes or tumor tissues was performed following established protocols (Chen et al, 2025). Experimental mice were euthanized in accordance with institutional guidelines, and tissues were dissected under sterile conditions. The samples were immediately placed in cold phosphate-buffered saline (PBS, pH 7.4) to maintain cell viability. The tissues were mechanically disrupted using a TissueLyser II with pre-cooled stainless steel beads at 30 Hz for 2–3 min. The homogenized tissue was filtered through 100 µm and 70 µm cell strainers to remove debris. The filtered suspension was transferred to a digestion solution containing 0.25% pancreatin and 0.1% collagenase type I, which had been optimized for tissue-specific enzymatic breakdown. The mixture was incubated at 37 °C with gentle agitation for 4 h, with 10 µg/mL of DNase I added during the final hour to prevent cellular aggregation. Enzymatic digestion was stopped by adding ice-cold culture medium supplemented with 10% fetal bovine serum. The cell suspension was centrifuged at 12,000 rpm for 5 min at 4 °C. The resulting individual cells were further used for experiments, such as flow cytometry analysis.

#### Flow cytometry analysis

Flow cytometry was utilized to examine the growth of cancer cells, as well as the characteristics of bone marrow-derived dendritic cells (BMDCs) and various types of lymphocytes from lymph nodes or tumor tissues (Huang et al, 2021). To initiate the flow cytometry analysis, individual cells were isolated from lymph nodes or tumor tissues as mentioned in the previous section or collected from cultured BMDCs described in the following section. These cells were then labeled with specific antibodies attached to different fluorescent dyes as outlined in the Reagents and Antibodies section. The fluorescent-labeled antibodies were diluted between 1:200 and 1:800, depending on the specific antigen being targeted. Additionally, fluorescent dyes such as 7-AAD, CFSE, and Far Red were employed to mark distinct cell populations. Following labeling, the cells were incubated for 30 min at 4 °C to allow the antibodies to bind. Subsequently, the cells were filtered to eliminate any clumps or aggregates, spun at 1000 rpm, suspended in PBS, and loaded into the flow cytometer for analysis. The resulting FCS files were analyzed using FlowJo software. Initially, the flow cytometry data were gated to exclude cell debris and select specific cell types based on their forward scatter and side scatter characteristics. Dead cells were identified and excluded using 7-AAD stain. For tumor cell analysis, CD45-negative cells were chosen, while CD45-positive cells were selected for immune cell analysis. Detailed gating strategies and analysis methods for different cell types, if necessary, were further elaborated in the respective experimental procedures.

#### Observation of BMDC uptake of BTCs and characterization of BMDC maturation, activation, and antigen presentation

BMDCs were obtained from female C57BL/6 or BALB/c mice aged 8–10 weeks, following institutional animal care guidelines. The mice were euthanized by CO_2_ inhalation or cervical dislocation. The bone marrow was harvested by flushing the femur and tibia cavities with cold phosphate-buffered saline using a 25-gauge needle. The isolated cells were filtered through a 70-μm cell strainer, centrifuged at 500 × *g* for 5 min at 4 °C, and resuspended in RPMI-1640 medium supplemented with 10% heat-inactivated fetal bovine serum, 1% penicillin–streptomycin, 50 μM β-mercaptoethanol, 20 ng/mL recombinant murine interleukin-4, and 20 ng/mL granulocyte-macrophage colony-stimulating factor. The cells were cultured at 37 °C in 5% CO_2_. The non-adherent cells were removed on days 2 and 4, and the adherent cell clusters were transferred to new plates on day 6, with the medium changed every 2–3 days. After 12 days, the non-adherent cells with characteristic dendritic cell morphology were collected by gentle pipetting and centrifugation. To determine whether BMDCs are capable of engulfing BTCs or RTCs, the BTCs and RTCs were stained with green CFSE, while the BMDCs were stained with Far Red. The BMDCs were then mixed with BTCs or RTCs and cultured for 24 h. During the continuous culture process, a fully automated seven-dimensional dynamic live cell research platform was used to observe the morphological changes of both cell types. After the 24-h culture period, cell morphology was examined using a confocal microscope. At the same time, a flow cytometer was used to determine the proportion of BMDCs (defined as DCs that had engulfed BTCs or RTCs) that were positive for both CFSE and Far Red, as well as for CD45, CD11c, and DEC205. To analyze the characteristics of BMDC maturation, activation, and antigen presentation, the isolated BMDCs were exposed to BTC, RTC, or BLys for 24 h in 12-well plates. After exposure, the BMDCs were harvested, labeled with antibodies against CD86, CD80, CD40, SIINFEKL-MHC I, anti-PE-H-2Db, and MHC II, and analyzed by flow cytometry (Huang et al, 2021). The cytokines TNF-α, IFN-γ, and IL-6 secreted by BMDCs in the supernatant were measured using ELISA kits (Chen et al, 2025).

#### Analysis of distinct immune cell phenotypes present in draining lymph nodes and tumor tissues

Single cell suspensions were prepared from draining lymph nodes and tumor tissues for the experiments. CD8^+^ T lymphocytes were specifically analyzed using MHC class I tetramers loaded with neoantigens SIINFEKL and ADPGK. Detailed immunophenotyping was conducted to characterize T cell exhaustion states, focusing on terminally exhausted T cells co-expressing TIM-3 and PD-1, and progenitor exhausted T cells marked by CD44 and TCF1 co-expression. Tumor-infiltrating lymphocytes (TILs) were thoroughly evaluated, including functional and phenotypic assessments of various immune cell populations. For CD8^+^ T cells, the potential to secrete IFN-γ was determined to assess tumor-killing capabilities. Myeloid-derived suppressor cells (MDSCs) were identified by CD11b and Gr-1 surface markers. Regulatory T cells (Tregs) were characterized by concurrent expression of CD4, Foxp3, and CD25. Tumor-associated macrophages (TAMs) were distinguished by M2 polarization markers CD11b, F4/80, and CD206. Immunostaining was carried out using fluorescently labeled monoclonal antibodies, carefully diluted to optimize signal-to-noise ratio. Flow cytometry analysis utilized rigorous gating strategies: initial debris removal, live cell selection using 7-AAD viability dye, and immune cell identification via CD45 positivity. Subsequent multi-parameter gating allowed precise characterization of specific cell subsets (Chen et al, 2025).

#### Establishment of murine tumor models, vaccination, and observation

In order to investigate the anti-tumor growth and preventive metastasis effects, five murine tumor models (B16-F10, 4T1, MC38, 4T1, and LL/2) were induced in female C57BL/6 or BALB/c mice aged 6–8 weeks (Huang et al, 2023; Huang et al, 2020). In total, 5 × 10^6^ tumor cells were injected into the right flank for the therapeutic models, and 1 × 10^6^ 4T1 cells were injected via the tail vein for the metastatic model. Subsequently, the mice were divided into three groups of 10 mice each: BLys, RTC, and BTC. In the therapeutic models, each mouse received subcutaneous injections of 3 × 10^5^ to 5 × 10^5^ BTC or RTC. Approximately 5 micrograms of bacterial lysate protein, equivalent to the amount of bacterial lysate protein on the surface of 5 × 10^5^ BTC, was injected subcutaneously in the BLys group mice. The vaccine injections were administered on Days 0 and 7 following tumor cell inoculation. The monitoring of tumor images and volumes in the therapeutic tumor models was conducted every three days using a portable measuring device. In the metastatic 4T1 model, BALB/c mice received the same dose of BLys, RTC, or BTC model vaccine on Days −7 and 0. Subsequently, 1 × 10^6^ 4T1-luciferase tumor cells were injected on Day 0 to establish the metastatic tumor model. Fluorescence intensity in the mice of the 4T1 metastatic model was measured by administering an intraperitoneal injection of 300 mg/kg of D-fluorescein potassium before detection. Luminescence intensity imaging was captured using an IVIS Lumina II imaging system after the administration of D-fluorescein potassium. All mice were bred and maintained in a specific pathogen-free animal facility. The animal care and treatment protocols in this study adhere to the Guiding Principles for Animal Care and Use (China) and the Guidelines for Care and Use of Laboratory Animals of Hainan Medical University. All animal experiments were approved by the Animal Care and Use Committee of Hainan Medical University (HYLL-2014-008), ensuring that the maximum tumor burden of 3000 mm^3^ was not exceeded.

#### Observation of adverse effects

Bacterialized B16-F10, MC38, CT26, and LL/2 were used as four BTC model vaccines to investigate potential adverse effects in their syngeneic C57BL/6 or BALB/c mice aged 6–8 weeks over a 180-day period, as previously reported (Chen et al, 2025). These mice were not injected with tumor cells and were only subcutaneously injected with the corresponding BLys, RTC, and BTC model vaccines according to a protocol similar to that of preventive metastasis tumor models. Data were collected at half-month intervals to track the weight, lifespan, and well-being of mice using digital scales, continuous monitoring, and assessments of fur condition, eating habits, and behavioral changes. Fertility was evaluated by pairing females with males after two weeks of vaccination, observing mating behaviors, recording litter sizes, and noting the time from mating to birth, as well as maternal behaviors and offspring viability. Organs were harvested during tissue collection, fixed in formalin, and processed for histological analysis. Microscopic examination was conducted using an MT20 microscope with H&E staining to check for any morphological changes in major organs such as the heart, liver, spleen, lungs, and kidneys. Blood samples were taken via tail vein puncture and analyzed using a BS-240 Vet biochemical analyzer to assess bone marrow toxicity through white and red blood cell counts, and organ toxicity through measurements of creatinine, aspartate transaminase (AST), alanine transaminase (ALT), and lactate dehydrogenase (LDH) levels.

#### Detection of cellular immunity

Detection of cellular immunity was performed as previously reported (Chen et al, 2025). The killing ability of tumor cells by splenocytes from mice vaccinated with 4T1 or MC38 BTC was used as effector cells, and 4T1 or MC38 tumor cells were used as target cells. It was detected using a CytoTox 96 cytotoxicity assay kit. In brief, splenocytes and tumor cells at various ratios (5–40:1) were plated on 96-well plates in a final volume of 100 μl and then incubated in a humidified atmosphere (5% CO_2_ at 37 °C) for 6 h. Aliquots and reconstituted substrates (each 50 μl) were transferred to another flat-bottom 96-well plate and incubated in the dark at room temperature for 50 min. The experiment was terminated by adding 50 μl of stop solution, and the optical density (OD) values at 492 nm were obtained using an ELX808IU microplate reader. The percentage of target cell death at each Effector:Target ratio was calculated as previously reported. In this study, we used a technique from a previous study to identify specific cellular immunity. 4T1 or MC38 cells were labeled with CFSE (7 mM) for 20 min, and then 5 × 10^6^ cells were injected into the abdominal cavity of syngeneic BALB/c or C57BL/6 mice that had been vaccinated with BLys, RTC, or BTC. After 24 h, the mice were euthanized, and the cells in the abdominal cavity were collected. These cells were further labeled with Cy5-anti-CD45 antibody, and the live CD45-negative cells were sorted out to analyze cell proliferation generations and the percentage of the CFSE-labeled tumor cells using flow cytometry. Additionally, the live CD45^+^ and CD8+ cells in the abdominal cavity were sorted out to examine the expression of neoantigen-specific SIINFEKL or ADPGK tetramers on their cell surface. Furthermore, the live CD45^+^ and CD8^+^ cells producing IFN-γ, TNF-α, and IL-12 were isolated from the abdominal cavity of mice vaccinated with BLys, RTC, or BTC. These cells (1 ×  10^6^) were labeled with corresponding monoclonal antibodies conjugated with fluorescent-labeled antibodies for 30 min at 4 °C. The labeled cells were analyzed using flow cytometry, and the resulting FCS files were assessed with FlowJo Software.

#### Detection of humoral immunity

Tumor-specific IgG and its subtypes in the serum of mice vaccinated with BLys, RTC, or BTC in both 4T1 and MC38 tumor models were detected by western blotting (Huang et al, 2021). In brief, the proteins of the 4T1 or MC38 cell lysates were separated on 12% sodium dodecyl sulfate polyacrylamide gel electrophoresis (SDS-PAGE) gels and transferred onto polyvinylidene difluoride membranes using a Mini Transblot device. Thereafter, the membranes were then probed with anti-mouse IgG antibodies at a 1:300 dilution after blocking with 10% non-fat milk at 4 °C for 2 h. Subsequently, the membranes were incubated with chemiluminescence substrates A and B (1:1) for 30 min, and images were captured using an ECL system. In addition, the titers of IgG and its subtypes IgG1, IgG2a, IgG2b, and IgG3 were detected by ELISA kits (Wuhan Boster Biological Technology, China), according to the manufacturer’s protocol (Huang et al, 2021). Furthermore, the levels of splenic monocytes producing tumor-specific antibodies were measured using an ELISPOT assay. Specifically, 4T1 or MC38 tumor lysate (5 μg/well) was fixed onto the polyvinylidene difluoride membrane on the bottom of ELISPOT plates from Millipore. Splenic monocytes from mice vaccinated with BLys, RTC, or BTC were then incubated in the plates for 6 h, with the IgG antibody secreted by splenocytes acting as the primary antibody. Subsequently, a secondary anti-mouse IgG antibody was used to stain the ELISPOT membrane, following a procedure similar to the Western blotting assay described earlier, but with results appearing as spots rather than bands (Huang et al, 2021).

#### Adoptive transfer of splenocytes and antibodies

Adoptive transfer of splenocytes from mice vaccinated with BLys, RTC, or BTC to newly 4T1-bearing or MC38-bearing mice was performed as previously reported (Huang et al, 2021). In brief, splenocytes from mice that received vaccinations with BLys, RTC, or BTC were collected on day 14 post-vaccination. New recipient mice (n = 5) were then injected with 4T1 or MC38 tumor cells to establish the tumor models, following the same procedure as described in the related section above. Subsequently, 5 × 10^6^ splenocytes per mouse were transferred into the recipient mice by injecting them into the abdomen on day 0 after tumor model establishment. This transfer process was repeated two more times on days 5 and 10. On day 18, tumor images and volumes were captured using a portable TM900 system. To transfer antibodies adoptively, purified immunoglobulins were obtained by passing pooled sera from mice vaccinated with BLys, RTC, or BTC through a chromatography column. These antibodies, at a dose of 50 mg/kg in each mouse, were then administered to tumor-bearing recipient mice three times, following the same time points as the mice that received splenocytes. Subsequently, tumor images and volumes were recorded using a TM900 portable measuring device, as detailed in the related section above.

#### Calculation of synergistic indexes

The Synergistic Index (SI) was used to assess whether cellular and antibody immunities worked together to fight tumor growth and the effectiveness of BTC vaccination and anti-PD-1 combination therapy in inhibiting tumor growth (Chen et al, 2025; Huang et al, 2021). To evaluate the synergy of cellular and antibody immunities, splenocytes and antibodies obtained through adoptive transfer protocols described in the above section were used. Twenty BALB/c mice were injected intraperitoneally with CFSE-labeled 4T1 or MC38 cells and divided into four groups. The mice were then injected intraperitoneally with PBS, splenocytes (5 × 10^6^), antibodies (50 mg/kg), or a combination of both. Cells from the abdominal cavity were collected, stained, and analyzed using flow cytometry as described in the above section. The SI value was determined based on the count of CD45-negative tumor cells. The relative ratio of tumor cells (RRTC) in each group was calculated by comparing the cell count to the PBS group. The expected RRTC of the combination group was obtained by multiplying the RRTC of the splenocyte group by the RRTC of the antibody group. The SI value was then calculated by dividing the expected RRTC of the combination group by its observed RRTC. To assess the effectiveness of BTC vaccination and anti-PD-1 combination therapy in slowing tumor growth, tumor volume data from different treatment groups were analyzed. The relative ratio of tumor volume (RRTV) for each treatment group was calculated by comparing their mean values to the mean value of the control group treated with PBS. The expected RRTV for the combination therapy group was estimated by multiplying the RRTV of the individual anti-PD-1 and BTC groups. The SI value was then calculated by dividing the expected RRTV of the combination therapy group by its observed RRTV. An SI value above 1 indicates a synergistic anti-tumor effect.

#### Evaluation of the anti-tumor efficacy of combining BTC vaccination with anti-PD-1 antibody therapy

In this study, the 4T1 model was utilized to assess the efficacy of combining BTC vaccination and anti-PD-1 antibody treatment in inhibiting tumor growth (Chen et al, 2025). Female BALB/c mice were divided into four groups: PBS, anti-PD-1, BTC, and anti-PD-1 + BTC. The PBS group received injections of normal saline. The BTC group received three doses of BTC on days −7, 0, and 7. The anti-PD-1 group was administered the anti-PD-1 antibody six times at four-day intervals starting on day 2. The anti-PD-1 + BTC group received both BTC vaccination and anti-PD-1 antibody treatment. 4T1 cells were injected into all four groups via subcutaneous injection of 5 × 10^6^ cells, with five mice in each group on day 1. Tumor volumes were measured using a TM900 device as per the specified protocol described in the above relative section.

#### Heatmap construction

The heatmap graphs in this study were created using the “pheatmap” function from the R package of the same name, using the tumor volume and adverse effects data collected from our current study (Chen et al, 2025). The data was structured in an Excel file, with each sample data in a column and the data for each parameter or different observation time points in a row. The “read.csv(file)” function was used to import the Excel file into the R program as heatmap data, and the sample column-location information was provided as input to the pheatmap function for each parameter or observation time point. When the pheatmap function was executed, the corresponding heatmap was generated.

#### Statistical analysis

Statistical analyses were performed using GraphPad Prism software. Data are presented as mean ± standard deviation (SD). Unpaired t-tests were used for comparisons between two groups, while one- or two-way ANOVA with Tukey’s post hoc test was applied for multiple group comparisons. Statistical significance was set at *P* < 0.05. *, **, ***, and **** shown in the Figures indicate *P *< 0.05, *P* < 0.01, *P* < 0.001, and *P* < 0.0001, respectively.

## Supplementary information

Peer Review File

Movie EV1

Source data Fig. 1

Source data Fig. 2

Source data Fig. 3

Source data Fig. 4

Source data Fig. 5

Source data Fig. 6

Source data Fig. 7

Expanded View Figures

## Data Availability

This study includes no data deposited in external repositories. The source data of this paper are collected in the following database record: biostudies:S-SCDT-10_1038-S44321-026-00465-x.
