# Supplementary material for: Bacterialized tumor cells as vaccine
Source: EMBO Mol Med. 2026 Jun 19;18(7):2946–78. doi: 10.1038/s44321-026-00465-x (PMC13365548; doi:10.1038/s44321-026-00465-x)
Supplement: Supplementary file 2 — Movie EV1 [file 44321_2026_465_MOESM2_ESM.zip › Expanded_View_Movie_Legend.docx]

**Expanded View Movie Legends**

**Movie EV1. BTC promote DC phagocytosis and degradation.** Time-lapse imaging revealed rapid migration of moDCs towards BTC, resulting in tight cell-cell encapsulation. Within 30 minutes of co-culture, moDCs adopted a dendritic morphology and initiated partial phagocytosis of BTCs. Complete internalization and partial degradation of BTCs occurred within 60 minutes, with robust degradation evident at 120 minutes.
