# Supplementary material for: Bacterialized tumor cells as vaccine
Source: EMBO Mol Med. 2026 Jun 19;18(7):2946–78. doi: 10.1038/s44321-026-00465-x (PMC13365548; doi:10.1038/s44321-026-00465-x)
Supplement: Supplementary file 10 — Expanded View Figures [file 44321_2026_465_MOESM10_ESM.pdf]

Expanded View Figures

Figure EV1. Preparation and characterization of BTCs, related to Fig. 1.

(**A**) Five different types of BTCs, including B16-F10, 4T1, MC38, CT26, and LL/2 BTCs, were observed using a confocal microscope. FITC (green) was used to label bacterial lysates, DiI (red) for tumor cell membranes, and DAPI (blue) for nuclei.

 © The Author(s)

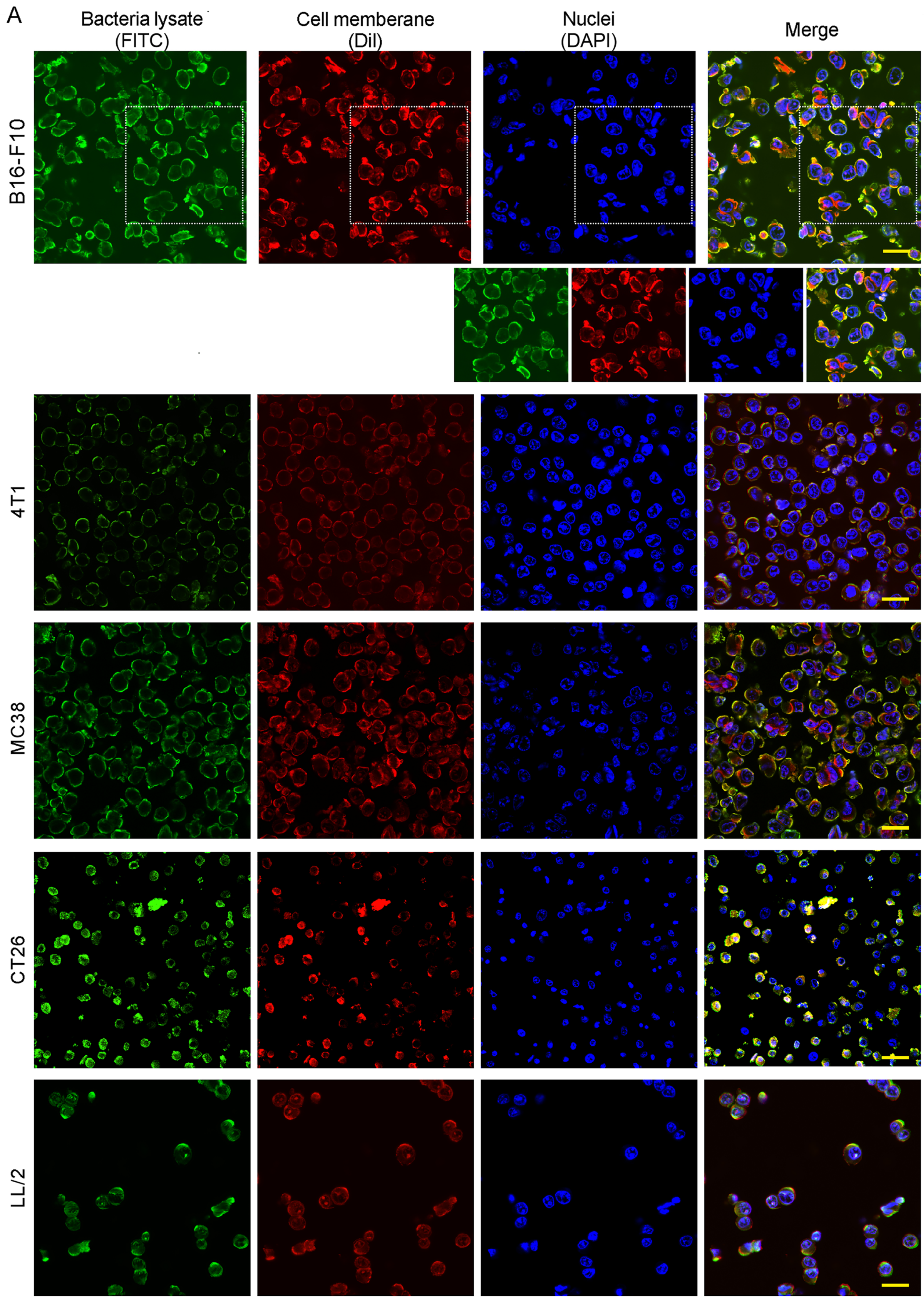
A
Bacteria lysate (FITC)
Cell memberane (DiI)
Nuclei (DAPI)
Merge
B16-F10
4T1
MC38
CT26
LL/2

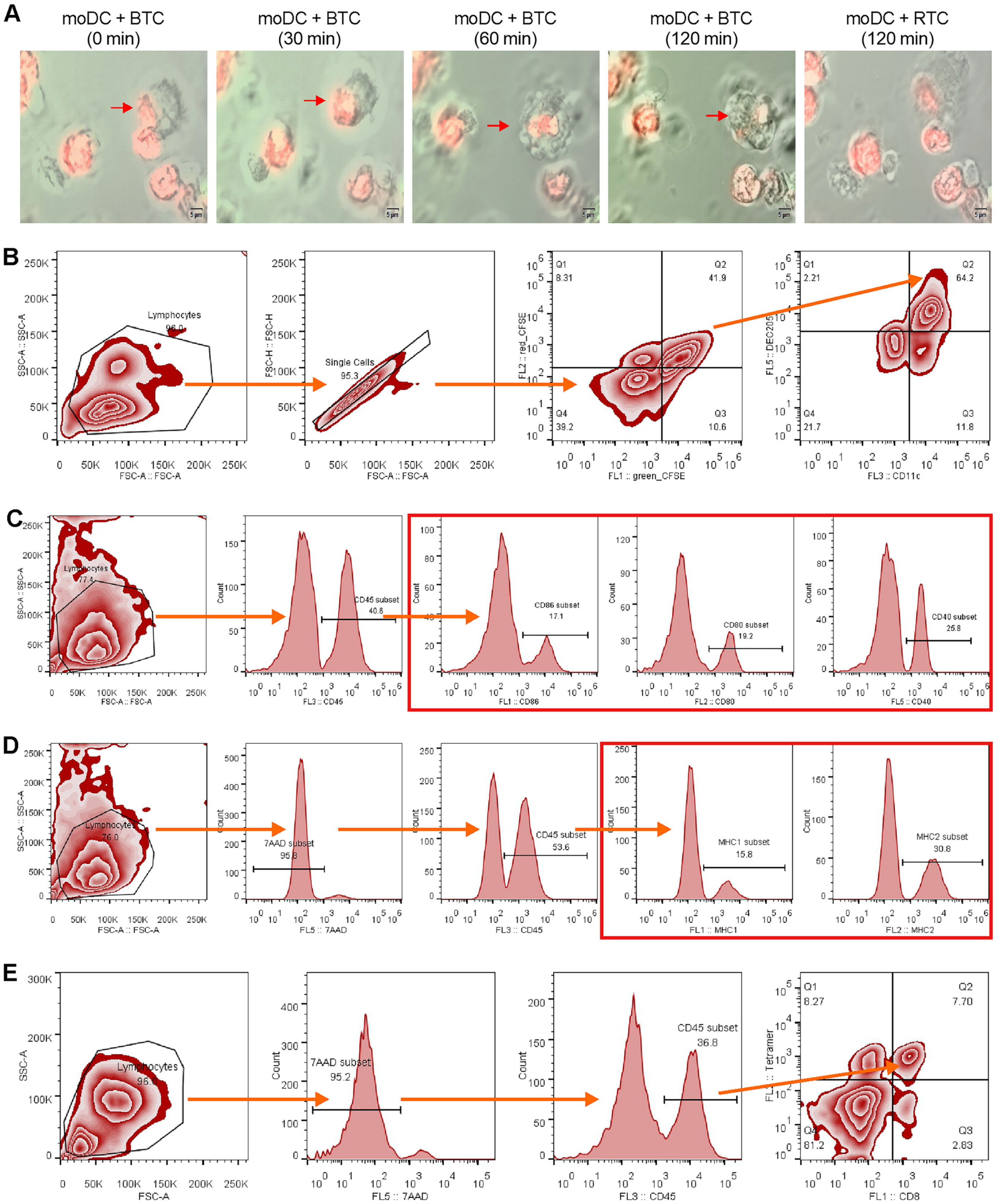
A
moDC + BTC (0 min)
moDC + BTC (30 min)
moDC + BTC (60 min)
moDC + BTC (120 min)
moDC + RTC (120 min)
5 μm
B
Lymphocytes
96.0
SSC-A :: SSC-A
FSC-A :: FSC-A
Single Cells
95.3
FSC-H :: FSC-H
Q1 8.31
Q2 41.9
Q4 39.2
Q3 10.6
FL2 :: red_CFSE
FL1 :: green_CFSE
Q1 2.21
Q2 64.2
Q4 21.7
Q3 11.8
FL5 :: DEC205
FL3 :: CD11c
C
Lymphocytes
77.4
CD45 subset
40.6
FL3 :: CD45
CD86 subset
17.1
FL1 :: CD86
CD80 subset
19.2
FL2 :: CD80
CD40 subset
25.8
FL5 :: CD40
Count
D
Lymphocytes
76.0
7AAD subset
95.8
FL5 :: 7AAD
CD45 subset
53.6
FL3 :: CD45
MHC1 subset
15.8
FL1 :: MHC1
MHC2 subset
30.8
FL2 :: MHC2
E
Lymphocytes
96.0
SSC-A
FSC-A
7AAD subset
95.2
FL5 :: 7AAD
CD45 subset
36.8
FL3 :: CD45
Q1 8.27
Q2 7.70
Q4 81.2
Q3 2.83
FL1 :: CD8
FL :: Tetramer

 © The Author(s)

Figure EV2. BTCs increase the ability of DCs to engulf, develop, and present antigens through cross-presentation, related to Fig. 1.

(**A**) Observation of the dynamic process of DCs engulfing and processing BTC. See also the Movie EV1. (**B**) The gating strategy for flow cytometry analysis in Fig. 1E. Debris and duplicate cells were excluded first, followed by gating out the green and far red CFSE-positive cells to analyze the CD11c and DEC205 double-positive cells. (**C**) The gating strategy for flow cytometry analysis in Fig. 1F–H. Debris was excluded first, followed by gating out the CD45-positive cells to analyze the CD86, CD80, and CD40-positive cells, respectively. (**D**) The gating strategy for flow cytometry analysis in Fig. 1L,M. Debris was excluded first, followed by gating out the 7-AAD-negative and CD45-positive cells to analyze the MHC1- or MHC2-positive cells. (**E**) The gating strategy for flow cytometry analysis in Fig. 1N–Q. Debris was excluded first, followed by gating out the 7-AAD-negative and CD45-positive cells to analyze the CD8 and tetramer double-positive cells.

© The Author(s)

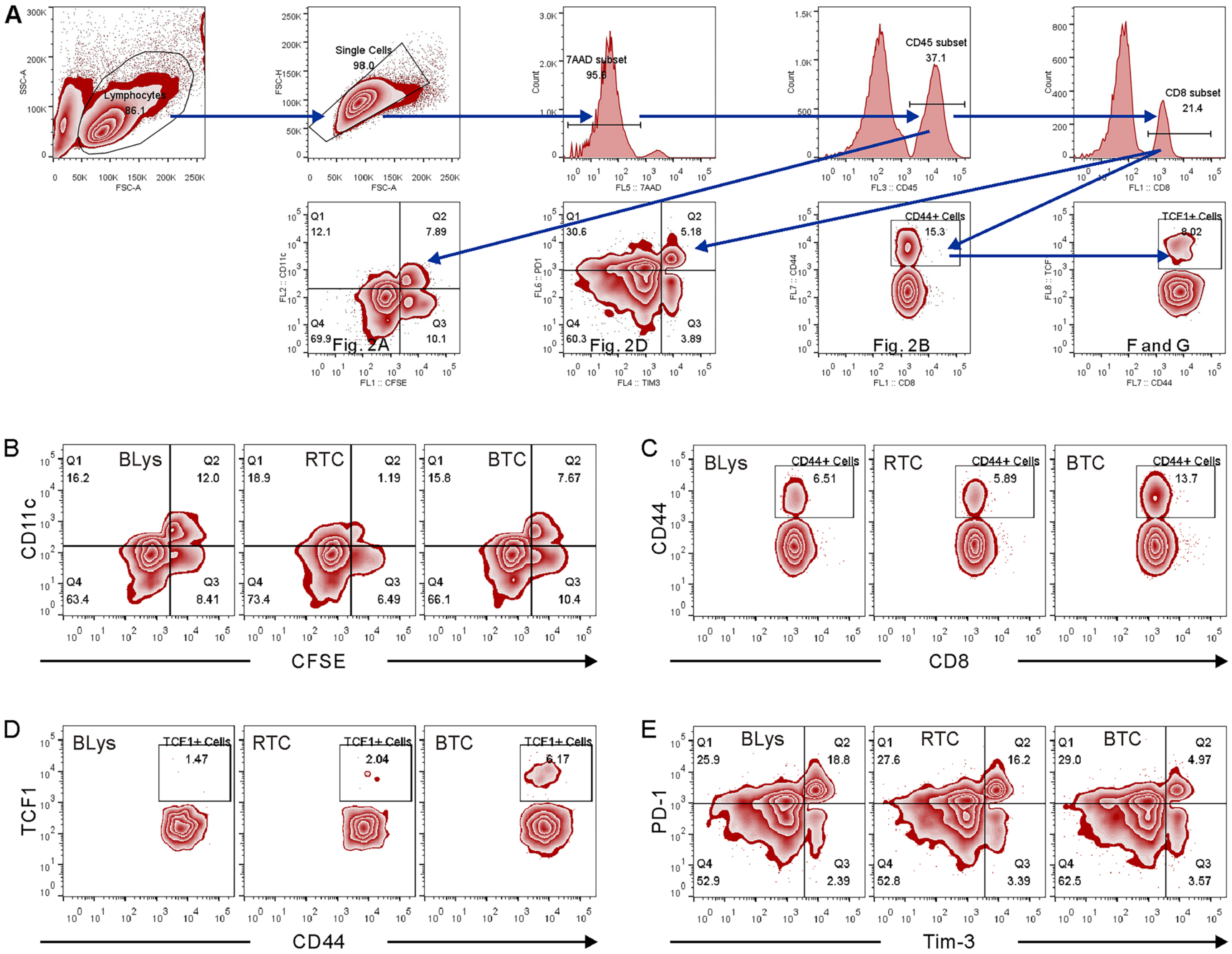

Figure EV3. BTCs facilitate DC migration and reshape the immune microenvironment in draining lymph nodes, related to Fig. 2.

(**A**) The gating strategy for flow cytometry analysis in Fig. 2. Debris was excluded first, followed by gating out the 7-AAD-negative and CD45-positive cells to analyze the CFSE and CD11c double-positive cells or CD8-positive cells. The CD8-positive cells were further analyzed for TIM3 and PD1 double-positive or CD44-positive cells, which were then further analyzed for TCF-1-positive cells. (**B**) Representative images of flow cytometry analysis (MC38) related to Fig. 2A. (**C**) Representative images of flow cytometry analysis (MC38) related to Fig. 2B. (**D**) Representative images of flow cytometry analysis (MC38) related to Fig. 2C. (**E**) Representative images of flow cytometry analysis (MC38) related to Fig. 2D.

 © The Author(s)

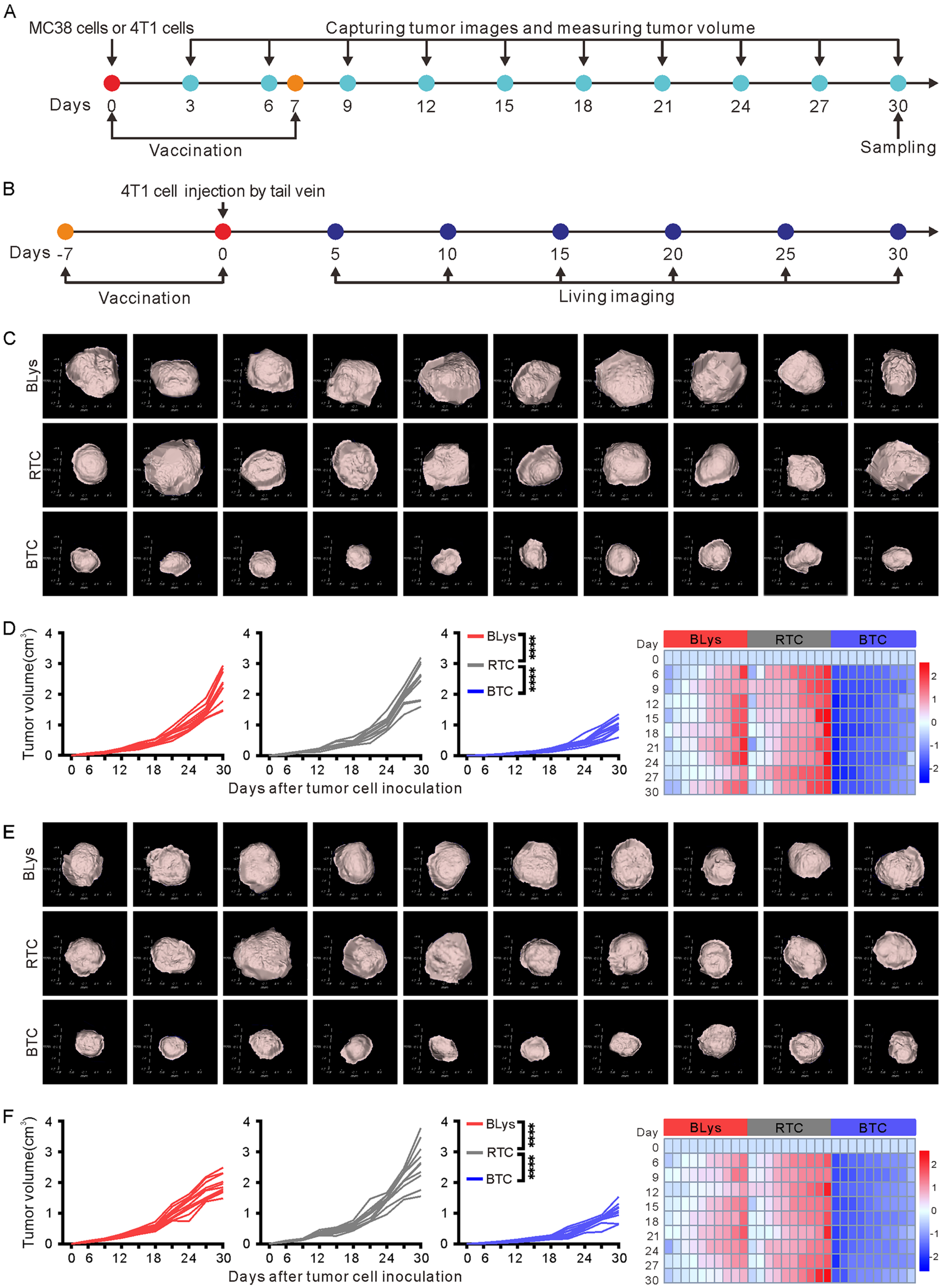
A
MC38 cells or 4T1 cells
Capturing tumor images and measuring tumor volume
Days 0 3 6 7 9 12 15 18 21 24 27 30
Vaccination
Sampling
B
4T1 cell injection by tail vein
Days -7 0 5 10 15 20 25 30
Vaccination
Living imaging
C
BLys
RTC
BTC
D
Tumor volume(cm³)
Days after tumor cell inoculation
BLys
RTC
BTC
****
****
Day
E
BLys
RTC
BTC
F
Tumor volume(cm³)
Days after tumor cell inoculation
BLys
RTC
BTC
****
****
Day

Figure EV4. BTC vaccination results in strong anti-tumor reactions, related to Fig. 3.

(**A**) Schematic presentation of the animal experimental protocol related to Fig. 3A. (**B**) Schematic presentation of the metastatic experimental protocol related to Fig. 3E. (**C**, **D**) The tumor images of the MC38 model on day 21 after tumor cell injection (**C**), and the tumor growth curve and heatmap presentation (**D**). (**E**, **F**) The tumor images of the CT26 model on day 21 after tumor cell injection (**E**), and the tumor growth curve and heatmap presentation (**F**). Data are presented as mean ± SD ($N = 10$ samples). Two-way ANOVA with Tukey multiple comparisons: ****$P < 0.0001$.

 © The Author(s)

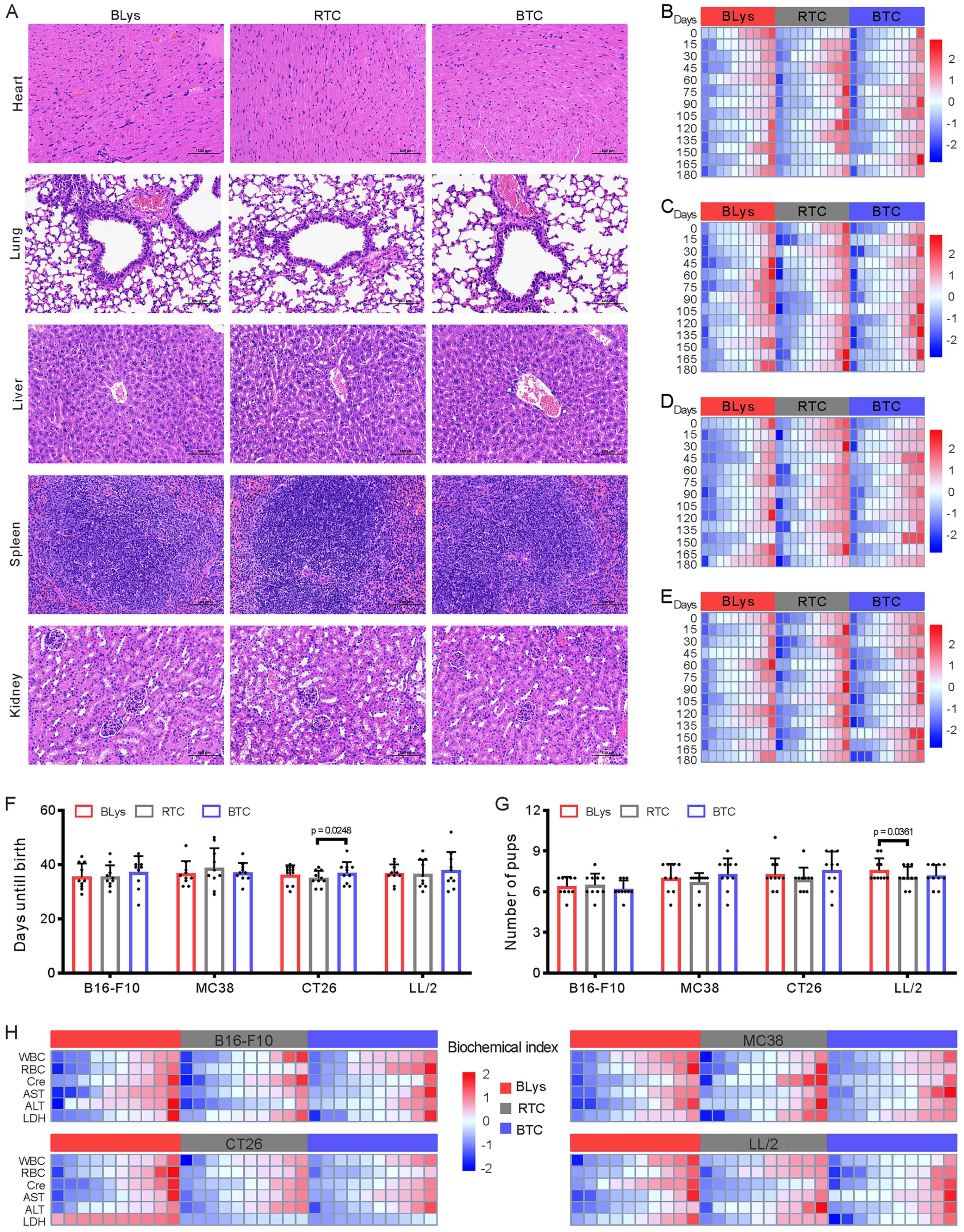
A
BLys
RTC
BTC
Heart
Lung
Liver
Spleen
Kidney
B
C
D
E
Days
0 15 30 45 60 75 90 105 120 135 150 165 180
2 1 0 -1 -2
F
Days untill birth
p = 0.0248
B16-F10
MC38
CT26
LL/2
G
Number of pups
p = 0.0361
H
WBC
RBC
Cre
AST
ALT
LDH
Biochemical index

Figure EV5. BTC vaccination does not cause significant adverse reactions, related to Fig. 3.

(**A**) The heart, lung, liver, spleen, and kidney tissues of the mice that were administered vaccinations of BLys, RTC, or BTC were subjected to staining with H&E. (**B–E**) Heatmaps illustrating the body weight of each mouse on every observed day in syngeneic mice vaccinated with B16-F10 (**B**), MC38 (**C**), CT26 (**D**), or LL/2 (**E**). (**F**) There was no significant variation in the mean time to first parturition among the syngeneic mice vaccinated with BLys, RTC, or BTC. (**G**) The average number of pups born did not exhibit any significant variances among the syngeneic mice that received vaccinations of BLys, RTC, or BTC. (**H**) Heatmaps illustrate the relative levels of peripheral blood WBC and RBC counts, as well as the biochemical indicators Cre, AST, ALT, and LDH, in syngeneic mice that were vaccinated with BLys, RTC, or BTC. Data are presented as mean ± SD ($N = 10$ samples). Two-way ANOVA with Tukey multiple comparisons.

 © The Author(s)

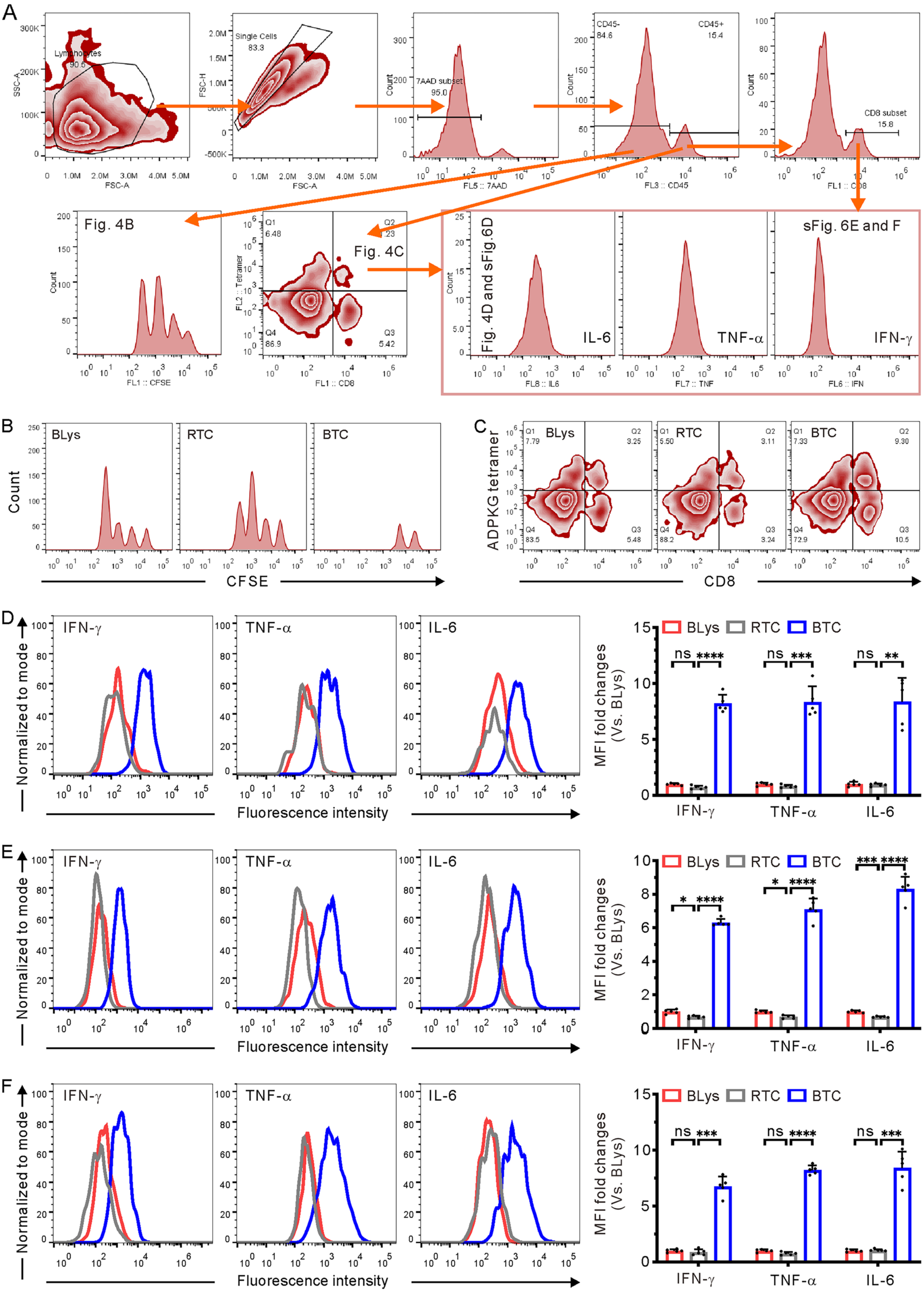

A
Fig. 4B
Fig. 4C
Fig. 4D and sFig.6D
sFig. 6E and F
IL-6
TNF-α
IFN-γ
B
BLys
RTC
BTC
Count
CFSE
C
ADPKG tetramer
CD8
D
E
F
Normalized to mode
Fluorescence intensity
MFI fold changes (Vs. BLys)

Figure EV6. BTC vaccination results in the generation of antigen-specific cellular immunity, related to Fig. 4.

(**A**) The gating strategy for flow cytometry analysis in Fig. 4. Debris and duplicate cells were excluded first, followed by gating out the 7-AAD-negative and CD45-positive cells. These cells were then used to analyze cell proliferation generations (CFSE-positive cells), CD8 and tetramer double-positive cells, which were used to analyze the IL-6, TNF-α, or IFN-γ-positive cells. The CD8-positive cells were also used to analyze the IL-6, TNF-α, or IFN-γ-positive cells. (**B**) Representative images of flow cytometry analysis (MC38) related to Fig. 4B. (**C**) Representative images of flow cytometry analysis (MC38) related to Fig. 4C. (**D**) $CD45^+CD8^+Tetramer^+$ lymphocytes isolated from tumor masses of syngeneic mice (MC38) that were immunized with BLys, RTC, or BTC were subsequently assessed for their levels of secretion of IFN-γ, TNF-α, and IL-6. (**E**, **F**) Non-antigen-specific $CD45^+CD8^+$ cells isolated from tumor masses of syngeneic mice 4T1 (**E**) or MC38 (**F**) that were immunized with BLys, RTC, or BTC were subsequently assessed for their levels of secretion of IFN-γ, TNF-α, and IL-6. Data are presented as mean ± SD ($N = 5$ independent experiments). Two-way ANOVA with Tukey multiple comparisons: *$P < 0.05$, **$P < 0.01$, ***$P < 0.001$, ****$P < 0.0001$, NS: non-significant.

© The Author(s)

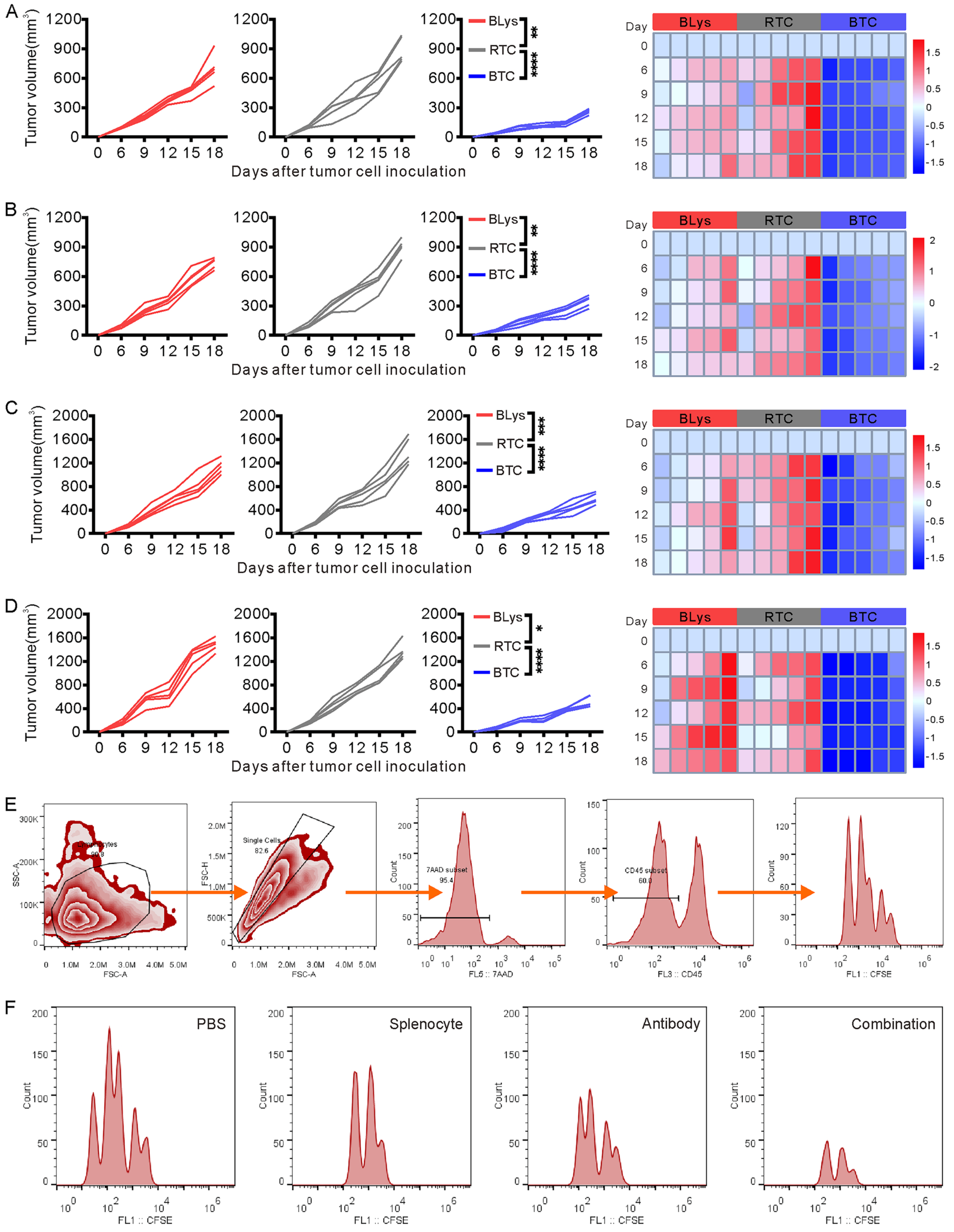
A
B
C
D
E
F
Tumor volume(mm3)
Days after tumor cell inoculation
BLys
RTC
BTC
Day
Lymphocytes
Single Cells
82.6
7AAD subset
95.4
CD45 subset
60.0
SSC-A
FSC-A
FSC-H
Count
FL5 :: 7AAD
FL3 :: CD45
FL1 :: CFSE
PBS
Splenocyte
Antibody
Combination

Figure EV7. Combination of cellular and humoral immunity results in synergistic anti-tumor effects, related to Fig. 5.

(**A**, **B**) The tumor growth curve and heatmap visualization of tumor volume in 4T1 (**A**) or MC38 (**B**) model mice treated with lymphocytes from syngeneic mice vaccinated with BLys, RTC, or BTC. (**C**, **D**) The tumor growth curve and heatmap visualization of tumor volume in 4T1 (**C**) or MC38 (**D**) model mice treated with antibodies from syngeneic mice vaccinated with BLys, RTC, or BTC. (**E**) The gating strategy for flow cytometry analysis in Fig. 5H. Debris and duplicate cells were excluded first, followed by gating out the 7-AAD-negative and CD45-positive cells, which were used to analyze cell proliferation generations (CFSE-positive cells). (**F**) Representative images of flow cytometry analysis (MC38) related to Fig. 5H. Data are presented as mean ± SD ($N = 5$ samples). Two-way ANOVA with Tukey multiple comparisons: *$P < 0.05$, **$P < 0.01$, ****$P < 0.0001$.

 © The Author(s)

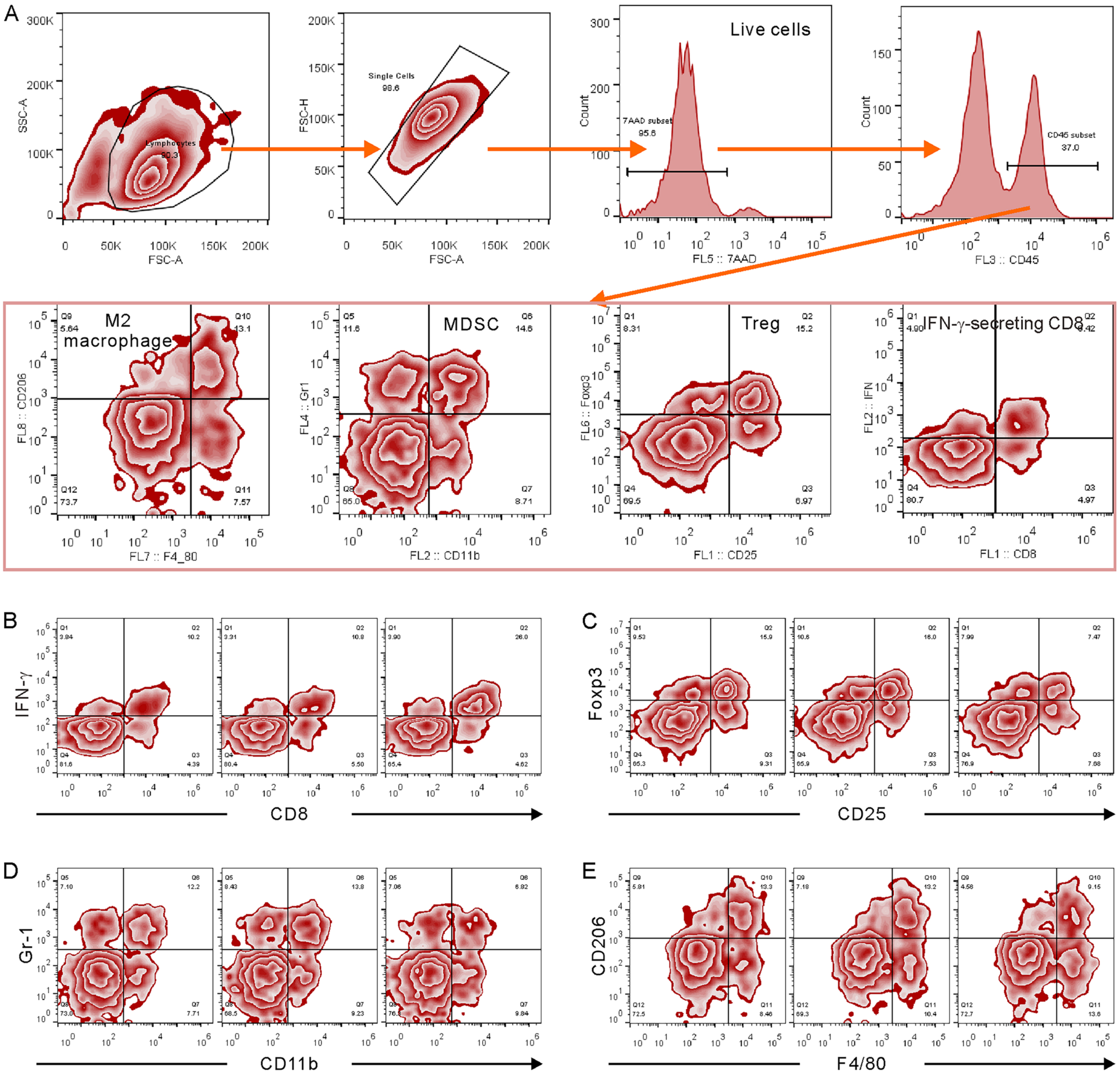

Figure EV8. BTC vaccination does not cause significant adverse reactions, related to Fig. 6.

(**A**) The gating strategy for flow cytometry analysis in Fig. 6. Debris and duplicate cells were excluded first, followed by gating out the 7-AAD-negative and CD45-positive cells. These cells were then used to analyze double-positive cells for F4-80 and CD206, CD11b and Gr1, CD25 and Foxp3, or CD8 and TNF-γ. (**B**) Representative images of flow cytometry analysis (MC38) related to Fig. 2A. (**C**) Representative images of flow cytometry analysis (MC38) related to Fig. 6B. (**D**) Representative images of flow cytometry analysis (MC38) related to Fig. 6C. (**E**) Representative images of flow cytometry analysis (MC38) related to Fig. 6D.
