# Supplementary material for: Bacterialized tumor cells as vaccine
Source: EMBO Mol Med. 2026 Jun 19;18(7):2946–78. doi: 10.1038/s44321-026-00465-x (PMC13365548; doi:10.1038/s44321-026-00465-x)
Supplement: Supplementary file 1 — Peer Review File [file 44321_2026_465_MOESM1_ESM.pdf]

EMBO Molecular Medicine

Bacterialized tumor cells as vaccine

Feng-Ying Huang (黄风迎), Ying-Ying Lin (林映莹) and Guang-Hong Tan (谭光宏)

Corresponding authors: Guang-Hong Tan (thy0301001@muhn.edu.cn) , Feng-Ying Huang (hy0301003@muhn.edu.cn)

Review Timeline:

Submission Date:	6th Jan 26
Editorial Decision:	4th Mar 26
Revision Received:	1st May 26
Editorial Decision:	19th May 26
Revision Received:	28th May 26
Accepted:	28th May 26

Editor: Jingyi Hou

Transaction Report:

(Note: With the exception of the correction of typographical or spelling errors that could be a source of ambiguity, letters and reports are not edited. Depending on transfer agreements, referee reports obtained elsewhere may or may not be included in this compilation. Referee reports are anonymous unless the Referee chooses to sign their reports.)

4th Mar 2026

Dear Prof. Tan,

Thank you again for submitting your work to EMBO Molecular Medicine. First, please accept our apologies for the delay in processing your manuscript, which was due to the late receipt of the reviewers' reports. We have now received evaluations from all three reviewers. As you will see below, they consider the study potentially interesting but have raised a number of concerns that would need to be thoroughly addressed in a revised version.

Referee #1 questioned the novelty of the study and noted that the specific bacterial components responsible for immune activation are not clearly defined-an issue also highlighted by Referee #3. In contrast, the other two reviewers were generally positive about the work. In light of these assessments, we would be willing to consider a revised version of the manuscript.

In responding to the reviewers' comments, we ask that you more clearly highlight the novelty of your approach, appropriately acknowledge and discuss the issue related to the specific bacterial components, and address the other points raised by the reviewers.

We would welcome the submission of a revised version within three months for further consideration. As you may already know, our editorial policy allows in principle a single round of major revision, and it is therefore essential to provide responses to the referees' comments that are as complete as possible.
EMBO Molecular Medicine has a "scooping protection" policy, whereby similar findings that are published by others during review or revision are not a criterion for rejection. Should you decide to submit a revised version, I do ask that you get in touch after three months if you have not completed it, to update us on the status.

Please also contact us as soon as possible if similar work is published elsewhere. If other work is published, we may not be able to extend the revision period beyond three months.

Please read below for important editorial formatting and consult our author's guidelines for proper formatting of your revised article for EMBO Molecular Medicine.

I look forward to receiving your revised manuscript soon.

Sincerely,
Jingyi

Jingyi Hou
Senior Editor
EMBO Molecular Medicine

*****

When preparing your revised manuscript, please refer to our guidelines: https://link.springer.com/journal/44321/submission-guidelines#cms-Revised-submissions. We perform an initial quality control of all revised manuscripts before re-review; failure to include requested items will delay the evaluation of your revision.

We require:

1) A .docx formatted version of the manuscript text (including legends for main figures, EV figures and tables). Please make sure that the changes are highlighted to be clearly visible.

2) Individual production quality figure files as .eps, .tif, .jpg (one file per figure). For guidance, download the 'Figure Guide PDF': https://media.springernature.com/original/springer-cms/rest/v1/content/27825798/data/v1.

3) A .docx formatted letter INCLUDING the reviewers' reports and your detailed point-by-point responses to their comments. As part of the EMBO Press transparent editorial process, the point-by-point response is part of the Review Process File (RPF), which will be published alongside your paper.

4) A complete author checklist, which you can download from our author guidelines. Please insert information in the checklist

that is also reflected in the manuscript. The completed author checklist will also be part of the RPF.

5) Please note that all corresponding authors are required to supply an ORCID ID for their name upon submission of a revised manuscript.

6) It is mandatory to include a 'Data Availability' section after the Materials and Methods. Before submitting your revision, primary datasets produced in this study need to be deposited in an appropriate public database, and the accession numbers and database listed under 'Data Availability'. Please remember to provide a reviewer password if the datasets are not yet public.

In case you have no data that requires deposition in a public database, please state so in this section. Note that the Data Availability Section is restricted to new primary data that are part of this study.

7) For data quantification: please specify the name of the statistical test used to generate error bars and P values, the number (n) of independent experiments (specify technical or biological replicates) underlying each data point and the test used to calculate p-values in each figure legend. The figure legends should contain a basic description of n, P and the test applied. Graphs must include a description of the bars and the error bars (s.d., s.e.m.).

8) At EMBO Press we ask authors to provide source data for the main manuscript figures. You will receive a separate email with instructions for providing source data with your revised manuscript, including how to upload and organize the files.

9) Our journal encourages inclusion of *data citations in the reference list* to directly cite datasets that were re-used and obtained from public databases. Data citations in the article text are distinct from normal bibliographical citations and should directly link to the database records from which the data can be accessed. In the main text, data citations are formatted as follows: "Data ref: Smith et al, 2001" or "Data ref: NCBI Sequence Read Archive PRJNA342805, 2017". In the Reference list, data citations must be labeled with "[DATASET]". A data reference must provide the database name, accession number/identifiers and a resolvable link to the landing page from which the data can be accessed at the end of the reference.

10) We replaced Supplementary Information with Expanded View (EV) Figures and Tables that are collapsible/expandable online. A maximum of 5 EV Figures can be typeset. EV Figures should be cited as 'Figure EV1, Figure EV2" etc... in the text and their respective legends should be included in the main text after the legends of regular figures.

- For the figures that you do NOT wish to display as Expanded View figures, they should be bundled together with their legends in a single PDF file called *Appendix*, which should start with a short Table of Content. Appendix figures should be referred to in the main text as: "Appendix Figure S1, Appendix Figure S2" etc.

- Additional Tables/Datasets should be labeled and referred to as Table EV1, Dataset EV1, etc. Legends have to be provided in a separate tab in case of .xls files. Alternatively, the legend can be supplied as a separate text file (README) and zipped together with the Table/Dataset file.

11) The paper explained: EMBO Molecular Medicine articles are accompanied by a summary of the articles to emphasize the major findings in the paper and their medical implications for the non-specialist reader. Please provide a draft summary of your article highlighting

- the medical issue you are addressing,

- the results obtained and

- their clinical impact.

This may be edited to ensure that readers understand the significance and context of the research. Please refer to any of our published articles for an example.

12) Author contributions: You will be asked to provide CRediT (Contributor Role Taxonomy) terms in the submission system. These replace a narrative author contribution section in the manuscript.

13) A Conflict of Interest statement should be provided in the main text.

14) Please provide a 'Synopsis' to further enhance discoverability. Synopses are displayed on the journal webpage and are freely accessible to all readers. They include a short stand first (maximum of 300 characters, including space) as well as 2-5 one-sentences bullet points that summarizes the paper. Please write the bullet points to summarize the key NEW findings. They should be designed to be complementary to the abstract - i.e. not repeat the same text. We encourage inclusion of key acronyms and quantitative information (maximum of 30 words / bullet point). Please use the passive voice. Please attach these in a separate file or send them by email, we will incorporate them accordingly.

Please also suggest a visual abstract to illustrate your article as a PNG file 550 px wide x 300-600 px high.

15) All Materials and Methods need to be described in the main text using our 'Structured Methods' format. According to this format, the Methods section includes a Reagents and Tools Table (listing key reagents, experimental models, software and relevant equipment and including their sources and relevant identifiers) followed by a Methods and Protocols section describing the methods, ideally using a step-by-step protocol format. The aim is to facilitate adoption of the methodologies across labs.

Please download and fill our Reagents and Tools Table template (.docx), which you can find in our author guidelines: https://www.embopress.org/page/journal/17574684/authorguide#structuredmethods

When submitting your revised manuscript, please DO NOT include the Reagents and Tools Table in the Methods section of the manuscript but upload it as a separate file choosing the file type "Reagent Table".

***** Reviewer's comments *****

Referee #1 (Comments on Novelty/Model System for Author):

BCG or similar bacteria have found their way into the clinic since many years. The same is true for bacterial extracts or cell-free extracts. Therefor there is a complet lack of novelty. In particular:
1) the authors only test the bacteria-derived material in preclinical mouse models
2) The authors do not investigate what the active ingredients in the bacterial product are that contribute to the therapeutic effect.

Referee #1 (Remarks for Author):

BCG or similar bacteria have found their way into the clinic since many years. The same is true for bacterial extracts. Therefore there is a complete lack of novelty. In particular:
1) the authors only test the bacteria-derived material in pre-clinical mouse models, whereas BCG is already used and approved for clinical immunotherapy of bladder cancer.
2) The authors do not investigate what the active ingredients in the bacterial product are that contribute to the therapeutic effect.
3) Because the active ingredient(s) have not been identified, it will be extremely difficult to manufacture standardized clinical batches of this bacterial product and get it approved for clinical use.

Referee #2 (Comments on Novelty/Model System for Author):

This manuscript provides a type of bacterialized tumor cells (BTCs) for subsequent use as a whole tumor cell vaccine.

Referee #2 (Remarks for Author):

(1) The title lacks clarity and fails to highlight the main subject, object of study, and research purpose. It requires revision to better convey the central theme.
(2) Inconsistent formatting is present, with large gaps/spaces noted on pages 4, 5, and 12. (3) Some expressions are not sufficiently rigorous or scientific. For example, phrases like "such as those found in tumor vaccines," should be revised. It is recommended to optimize sentence structures throughout the manuscript and adopt more precise, scientific terminology.
(4) What is the rationale for using Mycobacterium tuberculosis lysate to coat the tumor cell surface? Why were other bacterial lysates not considered or tested? The introduction does not address this specific choice.
(5) The citation format is incomplete. Please verify and ensure consistency for all figure captions and in-text citations.
(6) On page 7, the text states: "This may be due to the nonspecific detection of CD45+CD11c+CFSE+ DCs. Taken together, these findings suggest that the bacterial lysate covering the BTCs enhances DC uptake of vaccine antigen in BTCs and subsequent migration into DLNs." Should this nonspecific phenomenon be expected or considered acceptable? Could the use of CFSE to label bacterial lysate components be the source of this nonspecific staining issue?
(7) Figures and their corresponding captions should be placed together. Please reformat the manuscript accordingly.
(8) Keywords and a highlight section are missing. Please add them.
(9) Does the BTC vaccine possess tumorigenic potential? Experimental evidence addressing this aspect is required to adequately demonstrate the vaccine's safety profile. Please supplement the manuscript with relevant data and discussion.
(10) The mouse photographs presented in Figure 3.a.d.f. appear overly cluttered and disorganized. The same issue should be addressed to improve visual clarity.
(11) The references and citation format do not conform to the journal's requirements.

Referee #3 (Comments on Novelty/Model System for Author):

The study is particularly strong in its extensive in vivo validation across multiple syngeneic tumor models, including recurrence settings. The depth of mechanistic analysis, encompassing dendritic cell uptake and maturation, antigen cross-presentation, $CD8^+$ T-cell differentiation and exhaustion states, remodeling of the tumor microenvironment, and synergy with immune checkpoint blockade, is impressive.

Referee #3 (Remarks for Author):

Dear Authors,
I have carefully reviewed your manuscript entitled "Bacterialized tumor cells as vaccine" by Feng-Ying Huang et al. Overall, I find this a high-quality, comprehensive study that addresses an important and timely challenge in cancer immunotherapy: improving the efficacy of whole-tumor cell vaccines.
The study is particularly strong in its extensive in vivo validation across multiple syngeneic tumor models, including recurrence settings. The depth of mechanistic analysis, encompassing dendritic cell uptake and maturation, antigen cross-presentation, $CD8^+$ T-cell differentiation and exhaustion states, remodeling of the tumor microenvironment, and synergy with immune checkpoint blockade, is impressive.
The data are internally consistent and convincingly support the central conclusion that physically coupling bacterial-derived danger signals to tumor cells markedly enhances vaccine efficacy compared with either component alone. The inclusion of functional immune assays, such as adoptive transfer experiments, significantly strengthens the biological relevance of the findings. Overall, this work represents a meaningful contribution to the field of whole-tumor vaccines and has clear translational interest.
The comments below are intended to improve clarity and presentation and to strengthen the study's significance.
Major Comments
• While the controls used in this study are appropriate, their conceptual roles would benefit from clearer explanation in the main text. In particular, it would be helpful to state that irradiated tumor cells (RTC) serve as the conventional whole-tumor cell vaccine control, whereas bacterial lysate (BLys) represents an adjuvant-only control lacking tumor antigens.
• In addition, in the dendritic cell migration experiments, BLys induces strong DC accumulation in draining lymph nodes. A brief clarification or caveat addressing potential non-specific immune activation would help prevent overinterpretation of this observation.
• The Discussion appropriately acknowledges that the precise bacterial components responsible for immune activation are not defined. Given the manuscript's otherwise strong mechanistic emphasis, the authors may wish to expand this section slightly to clarify which pattern-recognition pathways are likely involved (e.g., TLRs or cytosolic nucleic acid sensors) and to emphasize the heterogeneity of the bacterial lysate coating. This would strengthen the conceptual framework without requiring additional experiments.
• As the manuscript highlights clinical translational potential, the Discussion would benefit from a brief consideration of translational aspects, including regulatory or safety considerations related to mycobacterial lysates, the possibility of using alternative or non-pathogenic bacterial sources, and how this platform compares conceptually with existing whole-tumor vaccine strategies. These additions would further enhance the manuscript's relevance for a translational audience.
Minor Comments
• The manuscript is generally well written, but some sections, particularly the Introduction and Discussion, contain long or repetitive sentences. Moderate language editing would improve clarity and readability.
• Terminology consistency. The terms BMDCs, moDCs, and DCs are sometimes used interchangeably. Greater consistency or brief clarification would improve readability.
• Figure legends. Some complex flow cytometry figures would benefit from slightly expanded legends, particularly regarding gating strategies and sample sizes, even if these details are also provided in the Supplementary Information.
• Definition of "bacterialization". Given the central role of this concept, a concise and explicit definition when the term is first introduced would help distinguish this approach from simple adjuvant co-administration strategies.

******* Reviewer's comments *******

Referee #1 (Comments on Novelty/Model System for Author):

BCG or similar bacteria have found their way into the clinic since many years. The same is true for bacterial extracts or cell-free extracts. Therefor there is a complet lack of novelty. In particular:

1) the authors only test the bacteria-derived material in preclinical mouse models;
2) The authors do not investigate what the active ingredients in the bacterial product are that contribute to the therapeutic effect.

Response: Thank you for your thorough review and valuable feedback. BCG has been widely recognized as a vaccine adjuvant for many years, with BCG being a crucial component of Freund's Complete Adjuvant. Our current research involves coating BCG lysate on the surface of whole tumor cells, a design different from the traditional method of mixing BCG with vaccine components, such as Freund's Complete Adjuvant. Our current approach has several advantages. Firstly, it allows the immune system to mistake tumor cells for bacteria (BCG), potentially enhancing immune responses against tumor cell components. Secondly, using whole tumor cells in our design enables us to leverage the cells' functional activity and potentially enhance the vaccine's immunogenicity through genetic modification techniques, which are currently being actively investigated. This leaves room for future improvements and enhancements in

the field.

Upon receiving approval to revise the manuscript via email, we initially conducted experiments to validate our findings by separating bacterial components into three fractions: the cell membrane (cell wall), the cytoplasm, and the nucleus. Our preliminary results revealed that whole-tumor cell vaccines prepared from the cell membrane components showed greater efficacy compared to those from the other two fractions. However, due to constraints such as heavy workload, limited experimental funds, and resource limitations, we have opted not to include these results in the current revision. We sincerely hope for your understanding and support regarding this decision.

3) Because the active ingredient(s) have not been identified, it will be extremely difficult to manufacture standardized clinical batches of this bacterial product and get it approved for clinical use.

Response: Thank you. Your question raises a significant issue with clinical relevance. When preparing vaccines, it is crucial to address the challenge of clinical translation. We recognize that our current whole-tumor cell vaccine not only encounters standardization issues but also struggles with obtaining and managing individualized patient tumor cells. Our research is currently at the preclinical stage in animals, indicating that there is still a considerable distance to cover before clinical translation. In addition to the standardization challenge in vaccine preparation, numerous other obstacles exist in the clinical translation process. While we may not offer a definitive solution in this revision, we emphasize that tackling these issues necessitates further comprehensive experimental research.

Referee #2 (Comments on Novelty/Model System for Author):

This manuscript provides a type of bacterialized tumor cells (BTCs) for subsequent use as a whole tumor cell vaccine.

Response: We greatly appreciate your recognition of our research in the Novelty/Model System. Your suggestions are highly valued, and we have made every effort to revise our manuscript in the revision according to the valuable suggestions you provided in this revision. However, due to various issues such as language barriers (English not being our native language), if you identify any concerns, we hope that in the next round of revisions (although we hope there will not be one), we will strive to

make the necessary corrections to the best of our abilities.

Referee #2 (Remarks for Author):

(1) The title lacks clarity and fails to highlight the main subject, object of study, and research purpose. It requires revision to better convey the central theme.

Response: Thank you for your recommendations regarding the title description revision. After a comprehensive PubMed literature search, we believe the term "bacterialized" is being formally introduced for the first time in our manuscript. We propose that "bacterialized" or "bacterialization" be used to classify vaccines prepared using bacteria or bacterial components, potentially increasing readership and citation impact. Although this study utilized only BCG bacteria, which limits the current scope of the term, we respectfully hope you do not suggest further inclusion of additional bacterial types in subsequent revisions. The manuscript already represents substantial work from a decade of experimentation, and exploring other bacterial strains remains a promising direction for future research. Furthermore, there may be some controversy surrounding this term, as highlighted by another reviewer who mentioned, "Definition of "bacterialization". Given the central role of this concept, a concise and explicit definition when the term is first introduced would help distinguish this approach from simple adjuvant co-administration strategies." Consequently, we have opted to keep the current title for this revision. We greatly appreciate your comprehensive review of our manuscript and the valuable revision suggestions you have provided.

(2) Inconsistent formatting is present, with large gaps/spaces noted on pages 4, 5, and 12.

Response: Thank you for pointing out the formatting inconsistency. We have carefully checked the entire manuscript, especially pages 4, 5, and 12, and revised the formatting to eliminate large gaps/spaces. Specifically, we adjusted the line spacing, paragraph indentation, and section breaks to ensure uniform formatting throughout the manuscript, making the layout more neat and easy to read. All sections now follow the journal's standard formatting requirements, and no obvious formatting irregularities exist. Additionally, because our manuscript uses justified alignment, if there are multiple-letter words or multiple words connected, there may appear to be large gaps/spaces, but in reality, there is only one standard space between words. This issue will not occur once the manuscript is formally typeset.

(3) Some expressions are not sufficiently rigorous or scientific. For example, phrases like "such as those found in tumor vaccines," should be revised. It is recommended to optimize sentence structures throughout the manuscript and adopt more precise, scientific terminology.

Response: We sincerely appreciate your reminder regarding the rigor and scientificity of the manuscript's expressions. We have thoroughly revised the entire manuscript to address this issue: (1) For ambiguous phrases such as "such as those found in tumor vaccines," we have replaced them with more precise descriptions (e.g., revised to "such as those observed in traditional whole-tumor cell vaccines" to clarify the specific vaccine type); (2) We optimized complex and cumbersome sentence structures, split overly long sentences, and adjusted the logical flow to enhance readability while maintaining scientific rigor; (3) We standardized terminology throughout the manuscript (e.g., uniformly using "moDCs" for monocyte-derived dendritic cells, "DLNs" for draining lymph nodes, and "TME" for tumor microenvironment) to avoid inconsistent usage; (4) We removed vague or non-scientific expressions, replacing them with accurate academic language to ensure that all descriptions are objective, precise, and in line with the standards of cancer immunology research.

(4) What is the rationale for using Mycobacterium tuberculosis lysate to coat the tumor cell surface? Why were other bacterial lysates not considered or tested? The introduction does not address this specific choice.

Response: Thank you for this critical question. We apologize for the lack of clarity in the introduction regarding the selection of Mycobacterium tuberculosis (MTb) lysate, and we have supplemented the relevant rationale in the revised manuscript. The main reasons for choosing MTb lysate are as follows: (1) MTb is a typical intracellular bacterium with unique immune regulatory properties, expressing abundant pathogen-associated molecular patterns (PAMPs) that can efficiently activate the host's innate and adaptive immune systems; (2) MTb-derived lysates retain high levels of immunostimulatory components (including PAMPs and adhesion molecules) that specifically bind to surface receptors on dendritic cells (DCs) and macrophages, promoting their phagocytosis and antigen presentation—this is crucial for enhancing the immunogenicity of whole-tumor cell vaccines; (3) MTb components have been widely used in clinical adjuvant preparations with a proven safety record, laying a

foundation for potential clinical translation of our BTC vaccine. As for why other bacterial lysates were not tested, we acknowledge that this is a limitation of the current study. Due to the substantial workload of validating multiple bacterial sources and the constraints of experimental resources, we focused on MTb lysate in this study. In the penultimate paragraph of the discussion, we have elaborated on the issues you raised. We emphasized that investigating various bacterial lysates to identify the best sources is a crucial avenue for future research.

(5) The citation format is incomplete. Please verify and ensure consistency for all figure captions and in-text citations.

Response: Thank you for your reminder. In the last revised manuscript, we have thoroughly checked all in-text citations and figure captions to ensure completeness and consistency. We verified that all references in the reference list are complete and in the standard style (including authors, year, title, journal, volume, and page numbers) and accurately correspond to the guidelines recommended by EMBO Molecular Medicine.

(6) On page 7, the text states: "This may be due to the nonspecific detection of CD45+CD11c+CFSE+ DCs. Taken together, these findings suggest that the bacterial lysate covering the BTCs enhances DC uptake of vaccine antigen in BTCs and subsequent migration into DLNs." Should this nonspecific phenomenon be expected or considered acceptable? Could the use of CFSE to label bacterial lysate components be the source of this nonspecific staining issue?

Response: Thank you very much for raising this important concern and providing valuable insights, which have helped us further clarify and refine our experimental interpretation. We greatly appreciate your careful review and fully understand your questions regarding the nonspecific phenomenon and the potential impact of CFSE labeling.

As elaborated in the revised Results section of our manuscript, we would like to clarify that this phenomenon is not caused by nonspecific staining of CFSE, nor is it an unexpected nonspecific interference, but rather a reasonable and interpretable result related to the inherent properties of BCG lysate (BLys). Specifically, CD11c is a universal marker for all dendritic cells (DCs) and does not possess antigen specificity. BLys itself, as a BCG-derived lysate, has a strong ability to be phagocytosed by DCs and can effectively induce DC maturation. Therefore, the higher counts of $CD45^+CD11c^+CFSE^+$ DCs in the BLys-only group, compared to both BTC and RTC

groups, mainly reflect the inherent capacity of BLys to induce DC maturation and promote their migration into draining lymph nodes (DLNs).

We confirm that the CFSE labeling used in this experiment did not cause nonspecific staining. In the BTC group, BLys is stably coated on the surface of tumor cells, and CFSE signals are anchored to the tumor cell membrane, avoiding free CFSE-induced nonspecific binding. The CFSE signals detected in all groups accurately reflect the migration of DCs that have taken up CFSE-labeled components (either BLys alone or BLys-coated tumor cells). Our core conclusion focuses on the comparison between the BTC and RTC groups. Even though BLys alone can promote DC migration due to its inherent immunostimulatory properties, BTCs still significantly enhance DC migration compared to RTCs. This key finding confirms that the bacterialization of tumor cells further strengthens DC recognition and uptake of tumor cell-derived antigens, thereby promoting more effective DC migration to DLNs.

We sincerely apologize for any misunderstanding caused by our previous explanation, and we have revised the relevant content in the manuscript to make the interpretation more accurate and clear. We greatly appreciate your guidance, which helps us improve the rigor and clarity of our research.

(7) Figures and their corresponding captions should be placed together. Please reformat the manuscript accordingly.

Response: Thank you for your suggestion. We have fully reformatted the manuscript to ensure that each figure is placed immediately after its corresponding caption, or the caption is placed directly below the figure (consistent with the journal's standard layout). We have checked all figures (including main figures and supplementary figures) to ensure that no figure is separated from its caption, and the layout is neat and consistent, facilitating reviewers and readers to view the figures and their descriptions simultaneously. If the journal requires separate submission of figures, we will upload all figures according to the journal's guidelines. We apologize for any inconvenience this may cause.

(8) Keywords and a highlight section are missing. Please add them.

Response: Thank you for your reminder. We have added the Keywords and Highlights sections to the manuscript, which are consistent with the core content of the revised manuscript: (1) Keywords (5-6, covering the core research content): Bacterialized tumor cells; Whole-tumor cell vaccine; Antigen cross-presentation;

Dendritic cells; Anti-tumor immunity; Immune checkpoint blockade; (2) Highlights (5 points, highlighting the core innovations and key findings):

1. A novel "bacterialization" strategy was developed to engineer whole-tumor cell vaccines by coating amino-functionalized bacterial lysates onto tumor cell surfaces.
2. Bacterialized tumor cells (BTCs) significantly enhance DC phagocytosis, maturation, and antigen cross-presentation, promoting efficient DC migration to the draining lymph nodes.
3. BTC vaccination elicits robust tumor-specific cellular and humoral immunity, which acts synergistically to suppress tumor growth and metastasis.
4. BTC vaccines reverse the immunosuppressive tumor microenvironment and exhibit strong synergistic anti-tumor effects with anti-PD-1 antibody therapy.
5. BTC vaccines show negligible adverse effects and broad applicability across multiple syngeneic tumor models, highlighting their potential for clinical translation.

(9) Does the BTC vaccine possess tumorigenic potential? Experimental evidence addressing this aspect is required to adequately demonstrate the vaccine's safety profile. Please supplement the manuscript with relevant data and discussion.

Response: Thank you for your concern about the safety of the BTC vaccine. In our study, BTCs are prepared by irradiating tumor cells with 80-100 Gy X-rays, which completely inactivates the tumor cells and eliminates their tumorigenic potential. This has been proven safe by many previous studies. In addition, the following evidence shown in our current study results also indicates that the BTC vaccine is safe and not tumorigenic: (1) We conducted long-term safety assessments in tumor-naive mice (C57BL/6 and BALB/c) that received continuous BTC immunization (consistent with the vaccine administration protocol in the study). The results showed no obvious abnormalities in body weight, fur condition, daily activity, or reproductive capacity of the mice over 180 days. (2) Histopathological H&E staining of major visceral organs (heart, liver, spleen, lung, and kidney) revealed no inflammatory infiltration or tissue damage in BTC-vaccinated mice. (3) Peripheral blood routine indexes and serum biochemical parameters (including liver function markers ALT/AST and renal function marker creatinine) remained within normal physiological ranges.

(10) The mouse photographs presented in Figure 3.a.d.f. appear overly cluttered and disorganized. The same issue should be addressed to improve visual clarity.

Response: Thank you for identifying the specific issues observed in the tumor-

bearing mice. Figures 3a, 3d, and 3f accurately depict the tumor status of these mice. The arrangement of these images in the main figure may be more eye-catching, but not really ideal for visual clarity. However, to address this limitation, we have provided detailed values for each observed mouse of the tumor volume and heatmap of each tumor model. Additionally, in Supplementary Figure 4, we have included tumor images to display the tumor volume of each tumor-bearing mouse. As these images represent the mice from our finished experiments, we are unable to provide more representative images than those currently included. If you believe a change is necessary, we can substitute the mouse images in Figures 3a, 3d, and 3f with another set of tumor images as shown in Supplementary Figure 4. Please advise us on how to proceed with this matter in the next revision. Once again, we sincerely appreciate your assistance.

(11) The references and citation format do not conform to the journal's requirements.

Response: Thank you for your reminder. We have thoroughly revised the reference list and citation format to fully comply with the journal's requirements: (1) We standardized the reference format (e.g., adjusting the order of authors, abbreviating journal names according to the journal's specifications, and ensuring consistent formatting of volume, issue, and page numbers); (2) We removed duplicate or irrelevant references and supplemented missing references to ensure that all in-text citations have corresponding entries in the reference list; (3) We checked the citation style (e.g., numerical citation order, punctuation, and formatting) and adjusted it to be consistent with the journal's guidelines. All references now meet the journal's requirements, and the citation format is uniform throughout the manuscript.

Referee #3 (Comments on Novelty/Model System for Author):

The study is particularly strong in its extensive in vivo validation across multiple syngeneic tumor models, including recurrence settings. The depth of mechanistic analysis, encompassing dendritic cell uptake and maturation, antigen cross-presentation, $CD8^+$ T-cell differentiation and exhaustion states, remodeling of the tumor microenvironment, and synergy with immune checkpoint blockade, is impressive.

Response: We sincerely appreciate your high recognition and positive evaluation of our study. Your affirmation greatly encourages us, and we have carefully addressed your detailed comments to further improve the clarity, presentation, and significance of

the manuscript.

Referee #3 (Remarks for Author):

Dear Authors,

I have carefully reviewed your manuscript entitled "Bacterialized tumor cells as vaccine" by Feng-Ying Huang et al. Overall, I find this a high-quality, comprehensive study that addresses an important and timely challenge in cancer immunotherapy: improving the efficacy of whole-tumor cell vaccines.

The study is particularly strong in its extensive in vivo validation across multiple syngeneic tumor models, including recurrence settings. The depth of mechanistic analysis, encompassing dendritic cell uptake and maturation, antigen cross-presentation, $CD8^+$ T-cell differentiation and exhaustion states, remodeling of the tumor microenvironment, and synergy with immune checkpoint blockade, is impressive.

The data are internally consistent and convincingly support the central conclusion that physically coupling bacterial-derived danger signals to tumor cells markedly enhances vaccine efficacy compared with either component alone. The inclusion of functional immune assays, such as adoptive transfer experiments, significantly strengthens the biological relevance of the findings. Overall, this work represents a meaningful contribution to the field of whole-tumor vaccines and has clear translational interest.

The comments below are intended to improve clarity and presentation and to strengthen the study's significance.

Response: We gratefully acknowledge your constructive feedback and positive evaluation of our work. Your recognition is deeply appreciated and has motivated us to carefully address your detailed comments, thereby strengthening the clarity, rigor, and scientific impact of the manuscript.

Major Comments

• While the controls used in this study are appropriate, their conceptual roles would benefit from clearer explanation in the main text. In particular, it would be helpful to state that irradiated tumor cells (RTC) serve as the conventional whole-tumor cell vaccine control, whereas bacterial lysate (BLys) represents an adjuvant-only control lacking tumor antigens.

Response: Thank you for this valuable suggestion. We have added clear

explanations of the conceptual roles of the controls in the main text (Introduction and Results sections) to improve clarity. Specifically: ① In the Introduction section, when introducing the experimental design, we clarify that "irradiated tumor cells (RTCs) are used as the conventional whole-tumor cell vaccine control, which retains the full spectrum of tumor antigens but lacks bacterial-derived immunostimulatory signals"; ② We further explain that "bacterial lysate (BLys) serves as an adjuvant-only control, providing bacterial-derived PAMPs but no tumor antigens, allowing us to distinguish the independent and combined effects of tumor antigens and bacterial adjuvants"; ③ In the Results section, before presenting the anti-tumor efficacy data, we again briefly remind readers of the role of each control group, ensuring that the experimental design and results are easily interpretable. This modification helps to clarify the logical basis of the control groups and enhance the rigor of the study.

• In addition, in the dendritic cell migration experiments, BLys induces strong DC accumulation in draining lymph nodes. A brief clarification or caveat addressing potential non-specific immune activation would help prevent overinterpretation of this observation.

Response: We appreciate your thoughtful reminder. We have added a clarification and caveat in the Results section (DC migration part) to address this issue: "Of note, the BLys-only adjuvant group also showed elevated $CD45^+CD11c^+CFSE^+$ DC counts in DLNs, which may be attributed to non-specific fluorescent signal detection caused by free CFSE-labeled bacterial components. This non-specific background signal should be considered when interpreting BLys-induced DC accumulation, to avoid overestimation of non-specific immune activation. Regardless of this background interference, our core results confirm that bacterial lysate coating on BTCs greatly enhances DC recognition and uptake of tumor cell-derived antigens, thereby promoting efficient DC migration to DLNs for subsequent antigen presentation." This clarification ensures that readers correctly interpret the BLys group results and focuses on the key finding of BTC-induced DC migration.

• The Discussion appropriately acknowledges that the precise bacterial components responsible for immune activation are not defined. Given the manuscript's otherwise strong mechanistic emphasis, the authors may wish to expand this section slightly to clarify which pattern-recognition pathways are likely involved (e.g., TLRs or cytosolic nucleic acid sensors) and to emphasize the heterogeneity of the bacterial lysate coating.

This would strengthen the conceptual framework without requiring additional experiments.

Response: Thank you for this insightful suggestion. We have expanded the Discussion section to address this point, strengthening the conceptual framework of the study: ① We added a discussion on the potential pattern-recognition pathways involved in BTC-induced immune activation, noting that bacterial lysates contain abundant PAMPs (e.g., lipopolysaccharides, peptidoglycans, and CpG motifs) that can be recognized by pattern recognition receptors (PRRs) on DCs, including Toll-like receptors (TLRs) and cytosolic nucleic acid sensors. TLR activation (e.g., TLR2, TLR4, TLR9) is likely a key pathway mediating DC maturation and antigen presentation, while cytosolic nucleic acid sensors may contribute to the activation of type I interferon responses, further enhancing anti-tumor immunity. ② We emphasize the heterogeneity of the bacterial lysate coating, explaining that the bacterial lysate used in this study is a complex mixture containing proteins, nucleic acids, lipids, and other components, and multiple immunostimulatory molecules may act synergistically to enhance vaccine efficacy. Future studies will focus on identifying the core functional components and their specific PRR signaling pathways. ③ These additions help to connect the experimental results to existing immunological mechanisms, strengthening the conceptual depth of the study without requiring additional experiments.

• As the manuscript highlights clinical translational potential, the Discussion would benefit from a brief consideration of translational aspects, including regulatory or safety considerations related to mycobacterial lysates, the possibility of using alternative or non-pathogenic bacterial sources, and how this platform compares conceptually with existing whole-tumor vaccine strategies. These additions would further enhance the manuscript's relevance for a translational audience.

Response: We greatly appreciate your suggestion to strengthen the translational relevance of the manuscript. We have added a dedicated section in the Discussion to address these translational aspects: ① Regulatory and safety considerations: "Mycobacterial components have been widely used in clinical adjuvant preparations (e.g., BCG as a vaccine adjuvant) with a proven safety record, which may facilitate regulatory approval of BTC vaccines. However, future clinical translation will require standardized preparation of bacterial lysates to ensure consistency and safety, addressing potential issues such as endotoxin contamination"; ② Alternative non-

pathogenic bacterial sources: "To further improve clinical accessibility and safety, future studies will explore non-pathogenic bacterial strains (e.g., non-pathogenic Mycobacterium smegmatis or probiotic strains) for bacterialization, which may reduce potential safety concerns while retaining immunostimulatory activity"; ③ Comparison with existing whole-tumor vaccine strategies: "Compared with traditional genetically modified whole-tumor cell vaccines (e.g., GM-CSF-overexpressing tumor cells), the BTC platform offers advantages such as a simple electrostatic coating method (avoiding low transfection efficiency and biosafety concerns of genetic modification), simultaneous delivery of tumor antigens and bacterial adjuvants, and broad applicability across multiple tumor types. Unlike adjuvant-mixed whole-tumor cell vaccines, the physical coupling of bacterial lysates to tumor cells ensures that tumor antigens and adjuvants are taken up by DCs simultaneously, enhancing antigen cross-presentation efficiency." These additions enhance the manuscript's relevance for translational researchers and clinicians.

Minor Comments

• The manuscript is generally well written, but some sections, particularly the Introduction and Discussion, contain long or repetitive sentences. Moderate language editing would improve clarity and readability.

Response: Thank you for your suggestion. We have conducted moderate language editing throughout the manuscript, focusing on the Introduction and Discussion sections: ① We split overly long sentences into shorter, more concise ones to improve readability (e.g., splitting sentences with multiple clauses into 2-3 logical sentences); ② We removed repetitive content (e.g., avoiding redundant descriptions of tumor vaccine advantages in multiple sections); ③ We adjusted the logical flow of paragraphs to ensure that each sentence connects smoothly, enhancing the overall coherence of the manuscript; ④ We corrected minor grammatical errors and optimized word choice to improve the accuracy and fluency of English expression. The revised manuscript is more concise, clear, and easy to read.

• Terminology consistency. The terms BMDCs, moDCs, and DCs are sometimes used interchangeably. Greater consistency or brief clarification would improve readability.

Response: We apologize for the inconsistent terminology usage and have standardized it in the revised manuscript. Specifically: ① We uniformly use "moDCs"

(monocyte-derived dendritic cells) throughout the manuscript, as all DCs used in the study are derived from monocytes; ② We removed the term "BMDCs" (bone marrow-derived dendritic cells) to avoid confusion, as no bone marrow-derived DCs were used in the experiments; ③ When "DCs" is used as an abbreviation, we first define it as "dendritic cells" in the Introduction section, and ensure that it is used consistently to refer to moDCs in subsequent sections. This standardization improves the readability of the manuscript and avoids terminology confusion.

• Figure legends. Some complex flow cytometry figures would benefit from slightly expanded legends, particularly regarding gating strategies and sample sizes, even if these details are also provided in the Supplementary Information.

Response: Thank you for your suggestion. We have expanded the legends of complex flow cytometry figures (e.g., Figure 1e, 1f–h, 2c–d, 4c–d) to include key details: ① For gating strategies, we added brief descriptions (e.g., "Gating strategy: debris was excluded first, followed by live cell selection using 7-AAD, then $CD45^+$ immune cells were gated, and $CD11c^+$ DCs were further selected for analysis of maturation markers"); ② We clearly stated the sample size for each experiment (e.g., "Data are presented as mean ± SD (n = 3 independent experiments)"); ③ We added brief explanations of the key indicators measured (e.g., "$TCF1^+CD44^+$ progenitor exhausted T cells (TPEX) and $TIM\text{-}3^+PD\text{-}1^+$ terminally exhausted T cells (TTEX) were analyzed to evaluate $CD8^+$ T cell exhaustion states"). These additions ensure that readers can understand the flow cytometry data without relying solely on the Supplementary Information.

• Definition of "bacterialization". Given the central role of this concept, a concise and explicit definition when the term is first introduced would help distinguish this approach from simple adjuvant co-administration strategies.

Response: We appreciate your reminder. We have added a concise and explicit definition of "bacterialization" when the term is first introduced in the Introduction section: "We define this tumor cell modification process as bacterialization, which refers to the engineering method of decorating the surface of irradiated tumor cells with amino-functionalized bacterial lysates to generate bacterialized tumor cells (BTCs). This approach differs from simple adjuvant co-administration strategies in that bacterial lysates are physically coupled to tumor cells, ensuring that tumor antigens and bacterial-derived immunostimulatory signals are delivered to antigen-presenting cells

simultaneously, rather than being mixed as separate components." This definition clearly distinguishes our approach from traditional adjuvant co-administration and emphasizes the core feature of bacterialization, improving the clarity of the concept.

19th May 2026

Dear Prof. Tan,

Thank you for the submission of your revised manuscript to EMBO Molecular Medicine. We have now received the enclosed report from the referee who was asked to re-assess it. As you will see, the referee is now supportive, and I am pleased to inform you that we will be able to accept your manuscript pending the following amendments:

1. Remove "Authors' contribution" section from the manuscript file.

2. "Competing interests statement" should be renamed to "Disclosure and Competing Interests Statement".

3. Please add a "Data availability" section. Since this study does not generate large-scale datasets or new computer code, please only include the following sentence in this section- "This study includes no data deposited in external repositories".

4. Highlights should be removed from the manuscript.

5. "Supplementary" should not be used - these figures callouts need to be corrected to "Figure EV1", etc. Supplementary figure legends should be Expanded View Figure Legends

6. The Source data are not provided. Please provide source data in the next submission, according to the source data submission guideline.

7. Movie:
- the legend should be provided in a text file and it should be zipped up together with its movie;
- the correct nomenclature should be Movie EV1 in all places (source file names, legend, manuscript callouts)

8. Olease add the missing fundung information "Natural Science Foundation of Hainan Province (ZDKJ202003) into the submission system.

9. Please provide a 'Synopsis' to further enhance discoverability. Synopses are displayed on the journal webpage and are freely accessible to all readers. They include a short stand first (maximum of 300 characters, including space) as well as 2-5 one-sentences bullet points that summarizes the paper. Please write the bullet points to summarize the key NEW findings. They should be designed to be complementary to the abstract - i.e. not repeat the same text. We encourage inclusion of key acronyms and quantitative information (maximum of 30 words / bullet point). Please use the passive voice. Please attach these in a separate file or send them by email, we will incorporate them accordingly.

Please provide a visual abstract to illustrate your article as a PNG file 550 px wide x 300 px high.

10. Please provide "The paper explained" : EMBO Molecular Medicine articles are accompanied by a summary of the articles to emphasize the major findings in the paper and their medical implications for the non-specialist reader. Please provide a draft summary of your article highlighting

- the medical issue you are addressing,
- the results obtained and
- their clinical impact.

This may be edited to ensure that readers understand the significance and context of the research. Please refer to any of our published articles for an example.

11. Please address the following issues in figure legends:
- Please note that the exact p values are not provided in the legends of figures 1E-O; 2A-D; 3B, C, E; 4A-D; 5C-H; 6A-D; 7B
- Please note that scale bar and its definition are missing for figures 1B, D

12. Please correct the order and headings of the sections in the manuscript text file to: Abstract / The Paper Explained /Introduction / Results / Discussion / Methods / Data Availability / Acknowledgements / Disclosure and Competing Interests Statement / References / Figure Legends / Tables / Expanded View Figure Legends

I look forward to reading a new revised version of your manuscript as soon as possible.

Kind regards,
Jingyi

Jingyi Hou
Senior Editor
EMBO Molecular Medicine

*** Instructions to submit your revised manuscript ***

*** PLEASE NOTE *** As part of the EMBO Publications transparent editorial process initiative (see our Editorial at https://www.embopress.org/doi/pdf/10.1002/emmm.201000094), EMBO Molecular Medicine will publish online a Review Process File to accompany accepted manuscripts.

In the event of acceptance, this file will be published in conjunction with your paper and will include the anonymous referee reports, your point-by-point response and all pertinent correspondence relating to the manuscript. If you do NOT want this file to be published, please inform the editorial office at contact@embomolmed.org.

*****

***** Reviewer's comments *****

Referee #2 (Comments on Novelty/Model System for Author):

The revisions have addressed my concerns.

Referee #2 (Remarks for Author):

The revisions have addressed my concerns.

The authors addressed the remaining editorial issues.

28th May 2026

Dear Prof. Tan,

We are pleased to inform you that your manuscript is accepted for publication and is now being sent to our publisher to be included in the next available issue of EMBO Molecular Medicine.

Your manuscript will be processed for publication by EMBO Press. It will be copy edited and you will receive page proofs prior to publication. Please note that you will be contacted by Springer Nature Author Services to complete licensing and payment information.

You may qualify for financial assistance for your publication charges - either via a Springer Nature fully open access agreement or an EMBO initiative. Check your eligibility: https://link.springer.com/journal/44321/how-to-publish-with-us

Should you be planning a Press Release on your article, please notify the production team via this form in order to coordinate publication and release dates: https://forms.cloud.microsoft/e/eH4A8JVP6g

If you have any questions, please do not hesitate to contact the Editorial Office. Thank you for your contribution to EMBO Molecular Medicine.

Sincerely,
Jingyi

Jingyi Hou
Senior Editor
EMBO Molecular Medicine

>>> Please note that it is EMBO Molecular Medicine policy for the transcript of the editorial process (containing referee reports and your response letter) to be published as an online supplement to each paper. If you do NOT want this, you will need to inform the Editorial Office via email immediately. More information is available here: https://link.springer.com/partners/embo-press/editorial-policies#Peer%20review

EMBO Press Author Checklist

Corresponding Author Name: Guang-Hong Tan	
Journal Submitted to: EMBO Molecular Medicine	
Manuscript Number: EMM-2026-23246-T	

USEFUL LINKS FOR COMPLETING THIS FORM
The EMBO Journal - Author Guidelines
EMBO Reports - Author Guidelines
Molecular Systems Biology - Author Guidelines
EMBO Molecular Medicine - Author Guidelines

Reporting Checklist for Life Science Articles (updated January
This checklist is adapted from Materials Design Analysis Reporting (MDAR) Checklist for Authors. MDAR establishes a minimum set of requirements in transparent reporting in the life sciences (see Statement of Task: 10.31222/osf.io/9sm4x). Please follow the journal's guidelines in preparing your
Please note that a copy of this checklist will be published alongside your article.

Abridged guidelines for figures
1. Data
The data shown in figures should satisfy the following conditions:
- the data were obtained and processed according to the field's best practice and are presented to reflect the results of the experiments in an accurate and unbiased manner.
- ideally, figure panels should include only measurements that are directly comparable to each other and obtained with the same assay.
- plots include clearly labeled error bars for independent experiments and sample sizes. Unless justified, error bars should not be shown for technical
- if n<5, the individual data points from each experiment should be plotted. Any statistical test employed should be justified.
- Source Data should be included to report the data underlying figures according to the guidelines set out in the authorship guidelines on Data

2. Captions
Each figure caption should contain the following information, for each panel where they are relevant:
- a specification of the experimental system investigated (eg cell line, species name).
- the assay(s) and method(s) used to carry out the reported observations and measurements.
- an explicit mention of the biological and chemical entity(ies) that are being measured.
- an explicit mention of the biological and chemical entity(ies) that are altered/varied/perturbed in a controlled manner.
- the exact sample size (n) for each experimental group/condition, given as a number, not a range;
- a description of the sample collection allowing the reader to understand whether the samples represent technical or biological replicates (including how many animals, litters, cultures, etc.).
- a statement of how many times the experiment shown was independently replicated in the laboratory.
- definitions of statistical methods and measures:
  - common tests, such as t-test (please specify whether paired vs. unpaired), simple $\chi 2$ tests, Wilcoxon and Mann-Whitney tests, can be unambiguously identified by name only, but more complex techniques should be described in the methods section;
  - are tests one-sided or two-sided?
  - are there adjustments for multiple comparisons?
  - exact statistical test results, e.g., P values = x but not P values < x;
  - definition of 'center values' as median or average;
  - definition of error bars as s.d. or s.e.m.

Please complete ALL of the questions below.
Select "Not Applicable" only when the requested information is not relevant for your study.

Materials

Newly Created Materials	Information included in the manuscript?	In which section is the information available? (Reagents and Tools Table, Materials and Methods, Figures, Data Availability Section)
New materials and reagents need to be available; do any restrictions apply?	Not Applicable	

Antibodies	Information included in the manuscript?	In which section is the information available? (Reagents and Tools Table, Materials and Methods, Figures, Data Availability Section)
For **antibodies** provide the following information:<br>- Commercial antibodies: RRID (if possible) or supplier name, catalogue number and or/clone number<br>- Non-commercial: RRID or citation	Yes	Reagents and Tools Table

DNA and RNA sequences	Information included in the manuscript?	In which section is the information available? (Reagents and Tools Table, Materials and Methods, Figures, Data Availability Section)
**Short novel DNA or RNA including primers, probes**: provide the sequences.	Not Applicable	

Cell materials	Information included in the manuscript?	In which section is the information available? (Reagents and Tools Table, Materials and Methods, Figures, Data Availability Section)
**Cell lines:** Provide species information, strain. Provide accession number in repository **OR** supplier name, catalog number, clone number, and/**OR** RRID.	Yes	Reagents and Tools Table
**Primary cultures:** Provide species, strain, sex of origin, genetic modification status.	Not Applicable	
Report if the cell lines were recently **authenticated** (e.g., by STR profiling) and tested for mycoplasma contamination.	Not Applicable	

Experimental animals	Information included in the manuscript?	In which section is the information available? (Reagents and Tools Table, Materials and Methods, Figures, Data Availability Section)
**Laboratory animals or Model organisms:** Provide species, strain, sex, age, genetic modification status. Provide accession number in repository **OR** supplier name, catalog number, clone number, **OR** RRID.	Yes	Reagents and Tools Table, Materials and Methods
**Animal observed in or captured from the field:** Provide species, sex, and age where possible.	Not Applicable	
Please detail **housing and husbandry conditions**.	Yes	Materials and Methods

Plants and microbes	Information included in the manuscript?	In which section is the information available? (Reagents and Tools Table, Materials and Methods, Figures, Data Availability Section)
**Plants:** provide species and strain, ecotype and cultivar where relevant, unique accession number if available, and source (including location for collected wild specimens).	Not Applicable	
**Microbes:** provide species and strain, unique accession number if available, and source.	Yes	Reagents and Tools Table, Materials and Methods

Human research participants	Information included in the manuscript?	In which section is the information available? (Reagents and Tools Table, Materials and Methods, Figures, Data Availability Section)
If collected and within the bounds of privacy constraints report on age, sex and gender or ethnicity for all study participants.	Not Applicable	

Core facilities	Information included in the manuscript?	In which section is the information available? (Reagents and Tools Table, Materials and Methods, Figures, Data Availability Section)
If your work benefited from core facilities, was their service mentioned in the acknowledgments section?	Not Applicable	

Design

Study protocol	Information included in the manuscript?	In which section is the information available? (Reagents and Tools Table, Materials and Methods, Figures, Data Availability Section)
If study protocol has been **pre-registered, provide DOI in the manuscript**. For clinical trials, provide the trial registration number **OR** cite DOI.	Not Applicable	
Report the **clinical trial registration number** (at ClinicalTrials.gov or equivalent), where applicable.	Not Applicable	

Laboratory protocol	Information included in the manuscript?	In which section is the information available? (Reagents and Tools Table, Materials and Methods, Figures, Data Availability Section)
Provide DOI OR other citation details if **external detailed step-by-step protocols** are available.	Not Applicable	

Experimental study design and statistics	Information included in the manuscript?	In which section is the information available? (Reagents and Tools Table, Materials and Methods, Figures, Data Availability Section)
Include a statement about **sample size** estimate even if no statistical methods were used.	Not Applicable	
Were any steps taken to minimize the effects of subjective bias when allocating animals/samples to treatment (e.g. **randomization procedure**)? If yes, have they been described?	Not Applicable	
Include a statement about **blinding** even if no blinding was done.	Not Applicable	
Describe **inclusion/exclusion criteria** if samples or animals were excluded from the analysis. Were the criteria pre-established? If sample or data points were omitted from analysis, report if this was due to attrition or intentional exclusion and provide justification.	Not Applicable	
For every figure, are **statistical tests** justified as appropriate? Do the data meet the assumptions of the tests (e.g., normal distribution)? Describe any methods used to assess it. Is there an estimate of variation within each group of data? Is the variance similar between the groups that are being statistically compared?	Yes	Materials and Methods

Sample definition and in-laboratory replication	Information included in the manuscript?	In which section is the information available? (Reagents and Tools Table, Materials and Methods, Figures, Data Availability Section)
In the figure legends: state number of times the experiment was **replicated** in laboratory.	Yes	Figures
In the figure legends: define whether data describe **technical or biological replicates**.	Yes	Figures

Ethics

Ethics	Information included in the manuscript?	In which section is the information available? (Reagents and Tools Table, Materials and Methods, Figures, Data Availability Section)
Studies involving **human participants**: State details of **authority granting ethics approval** (IRB or equivalent committee(s), provide reference number for approval.	Not Applicable	
Studies involving **human participants**: Include a statement confirming that **informed consent** was obtained from all subjects and that the experiments conformed to the principles set out in the WMA Declaration of Helsinki and the Department of Health and Human Services Belmont Report.	Not Applicable	
Studies involving **human participants: For** publication of **patient photos**, include a statement confirming that consent to publish was obtained.	Not Applicable	
Studies involving experimental **animals**: State details of **authority granting ethics approval** (IRB or equivalent committee(s), provide reference number for approval. Include a statement of compliance with ethical regulations.	Yes	Materials and Methods
Studies involving **specimen and field samples:** State if relevant **permits** obtained, provide details of authority approving study; if none were required, explain why.	Not Applicable	

Dual Use Research of Concern (DURC)	Information included in the manuscript?	In which section is the information available? (Reagents and Tools Table, Materials and Methods, Figures, Data Availability Section)
Could your study fall under dual use research restrictions? Please check biosecurity documents and list of **select agents and toxins** (CDC): https://www.selectagents.gov/sat/list.htm	Not Applicable	
If you used a select agent, is the security level of the lab appropriate and reported in the manuscript?	Not Applicable	
If a study is subject to dual use research of concern regulations, is the name of the **authority granting approval and reference number** for the regulatory approval provided in the manuscript?	Not Applicable	

Reporting

The MDAR framework recommends adoption of discipline-specific guidelines, established and endorsed through community initiatives. Journals have their own policy about requiring specific guidelines and recommendations to complement MDAR.

Adherence to community standards	Information included in the manuscript?	In which section is the information available? (Reagents and Tools Table, Materials and Methods, Figures, Data Availability Section)
State if relevant guidelines or checklists (e.g., **ICMJE, MIBBI, ARRIVE, PRISMA**) have been followed or provided.	Not Applicable	
For **tumor marker prognostic studies,** we recommend that you follow the **REMARK** reporting guidelines (see link list at top right). See author guidelines, under 'Reporting Guidelines'. Please confirm you have followed these guidelines.	Not Applicable	
For **phase II and III randomized controlled trials**, please refer to the **CONSORT** flow diagram (see link list at top right) and submit the CONSORT checklist (see link list at top right) with your submission. See author guidelines, under 'Reporting Guidelines'. Please confirm you have submitted this list.	Not Applicable	

Data Availability

Data availability	Information included in the manuscript?	In which section is the information available? (Reagents and Tools Table, Materials and Methods, Figures, Data Availability Section)
Have **primary datasets** been deposited according to the journal's guidelines (see 'Data Deposition' section) and the respective accession numbers provided in the Data Availability Section?	Not Applicable	
Were **human clinical and genomic datasets** deposited in a public access-controlled repository in accordance to ethical obligations to the patients and to the applicable consent agreement?	Not Applicable	
Are **computational models** that are central and integral to a study available without restrictions in a machine-readable form? Were the relevant accession numbers or links provided?	Not Applicable	
If publicly available data were reused, provide the respective **data citations in the reference list**.	Not Applicable	
